# Complex Exon-Intron Marking by Histone Modifications Is Not Determined Solely by Nucleosome Distribution

**DOI:** 10.1371/journal.pone.0012339

**Published:** 2010-08-23

**Authors:** Pawandeep Dhami, Peter Saffrey, Alexander W. Bruce, Shane C. Dillon, Kelly Chiang, Nicolas Bonhoure, Christoph M. Koch, Jackie Bye, Keith James, Nicola S. Foad, Peter Ellis, Nicholas A. Watkins, Willem H. Ouwehand, Cordelia Langford, Robert M. Andrews, Ian Dunham, David Vetrie

**Affiliations:** 1 The Wellcome Trust Sanger Institute, Wellcome Trust Genome Campus, Hinxton, United Kingdom; 2 Institute of Cancer Sciences, University of Glasgow, United Kingdom; 3 Department of Haematology, University of Cambridge and NHS Blood and Transplant Cambridge, Cambridge, United Kingdom; Wellcome Trust Centre for Stem Cell Research, United Kingdom

## Abstract

It has recently been shown that nucleosome distribution, histone modifications and RNA polymerase II (Pol II) occupancy show preferential association with exons (“exon-intron marking”), linking chromatin structure and function to co-transcriptional splicing in a variety of eukaryotes. Previous ChIP-sequencing studies suggested that these marking patterns reflect the nucleosomal landscape. By analyzing ChIP-chip datasets across the human genome in three cell types, we have found that this marking system is far more complex than previously observed. We show here that a range of histone modifications and Pol II are preferentially associated with exons. However, there is noticeable cell-type specificity in the degree of exon marking by histone modifications and, surprisingly, this is also reflected in some histone modifications patterns showing biases towards introns. Exon-intron marking is laid down in the absence of transcription on silent genes, with some marking biases changing or becoming reversed for genes expressed at different levels. Furthermore, the relationship of this marking system with splicing is not simple, with only some histone modifications reflecting exon usage/inclusion, while others mirror patterns of exon exclusion. By examining nucleosomal distributions in all three cell types, we demonstrate that these histone modification patterns cannot solely be accounted for by differences in nucleosome levels between exons and introns. In addition, because of inherent differences between ChIP-chip array and ChIP-sequencing approaches, these platforms report different nucleosome distribution patterns across the human genome. Our findings confound existing views and point to active cellular mechanisms which dynamically regulate histone modification levels and account for exon-intron marking. We believe that these histone modification patterns provide links between chromatin accessibility, Pol II movement and co-transcriptional splicing.

## Introduction

It is a widely held view that combinations of post-translational modifications on the N-terminal tails of histones are likely to function as an epigenetic code [1] to regulate aspects of gene expression, including the activity of cis-regulatory elements, and the three phases of transcription (initiation, elongation and termination). In support of this code, systematic studies of histone acetylation and methylation patterns across the human genome have revealed signatures for transcriptionally active and inactive promoters [2], [3], [4], [5], [6], distal elements/enhancers [2], [4], [6], and insulators [2], [7]. A number of these histone modifications have also been shown to co-localize with gene bodies of transcribed genes [2], [5], [8]. Until recently, the extent to which modifications in gene bodies contribute to the functional complexity of chromatin is not clear. Evidence pointed to H3K9ac, H3K9me2, H3K27me3, and H3K36me3, having roles in closing chromatin to prevent spurious initiation of transcription within gene bodies [9], [10], [11], and/or facilitating splicing [12], [13], [14]. What is clear is that expressed genes require a dynamic equilibrium between the relaxation and compaction of chromatin [15], and the displacement/replacement of nucleosomes [16], [17] as the RNA polymerase II (Pol II) complex moves through the gene during transcription [18].

The recent discovery that H3K36me3 marks exons within transcribed gene bodies provided the first genome-wide evidence that coding features of all expressed genes may also have specific epigenetic signatures related to co-transcriptional splicing [12]. Subsequently, a number of studies provided clues as to the extent of this marking; a large number of histone modifications showed higher levels across exons which, for the most part, could be accounted for by nucleosome distribution, with well-positioned nucleosomes on exons accounting for these patterns [19], [20], [21], [22], [23]. Higher levels of Pol II occupancy were also associated with exons when compared to introns [19], suggesting that Pol II movement is affected by nucleosome positioning. However, the exact relationships between histone modifications, nucleosome distribution, Pol II movement and splicing across transcribed genes are not yet clear, although recent evidence points to a role for H3K36me3 in regulating the splicing machinery [24].

In this context, we sought to more accurately define the distribution of a variety of histone modifications within gene bodies across the human genome across several cell types, and relate these patterns to hallmarks of transcriptional activity and chromatin structure. Our data further supports the existence of a complex chromatin-based marking system for exon-intron structures across the human genome. Histone modifications are primarily associated with exons, but some also show higher levels in introns. Surprisingly, this exon-intron marking is intrinsic to most genes, irrespective of their transcriptional status, although the type of marks found on transcriptionally active or inactive genes do differ. We provide evidence that this marking is not accounted for by nucleosome distribution and points to active mechanisms which lay down these marks across exons or introns of both expressed and non-expressed genes. Our data supports the hypotheses that histone modifications may regulate chromatin accessibility and Pol II movement during transcription and co-transcriptional splicing, and may also “prime” exon-intron structures prior to transcription.

## Results

### Histone Modifications Mark Exon-Intron Structures of Expressed and Non-expressed Genes in a Cell-type Specific Manner

We performed chromatin immunoprecipitation in combination with microarrays (ChIP-chip) for 19 histone modifications, Pol II, histone density, and chromatin accessibility (FAIRE) in two hematopoietic cell lines (erythroid K562 and monocytic U937) and primary CD14^+^ monocytes. FAIRE (formaldehyde-assisted isolation of regulatory elements) assays allow DNA segments which are less readily cross-linked with proteins after formaldehyde treatment (i.e., regions of accessibility or DNase I hypersensitivity) to be physically separated from bulk cross-linked chromatin using phenol-chloroform fractionation [25], [26]. We analyzed the output of these assays, in the first instance, on our bespoke (custom-made) tiling path microarray covering 30 Mb of the human genome constituting the pilot regions of the ENCODE project [4]. The ENCODE pilot regions have been shown to be strong indicators of trends across the whole genome [3]. In parallel, we determined expression profiles for our three cell types (see Materials and Methods). To account for non-specific enrichments from ChIP and differences in nucleosome density, we normalized our datasets with the non-specific antisera ChIP profiles [27] and for H3/H2B histone density. This normalization step would effectively remove any biases in histone modification profiles which could be attributed to differences in nucleosome distribution. We determined the distribution of histone modifications with respect to the “ON/OFF” expression status of genes, their overall chromatin landscape, and exon/intron structures. We subsequently profiled four of these histone modifications across the whole human genome (see below).

Consistent with our previous observations and with those of others [2], [4], [5], we found four generalized trends for histone modifications across consensus plots for expressed genes in all three cell types (Supplementary Figures S1, S2, S3, S4). These included histone modifications with a substantial promoter/transcriptional start site (TSS) bias (e.g. H3K4me2 and H3K4me3), those extending into gene bodies with either a 5′ or 3′ enrichment biases relative to gene structures (e.g. H3K79me3 and H3K36me3 respectively), and those depleted across gene bodies (e.g. H3K36me1). Non-expressed genes showed hallmark patterns of enrichment for the repressive modifications (e.g. H3K9me2 and H3K27me3). Patterns for histone density and FAIRE were consistent with open, nucleosome-poor and closed, nucleosome-rich chromatin states for expressed and non-expressed genes respectively (Supplementary Figure S5).

**Figure 1:**
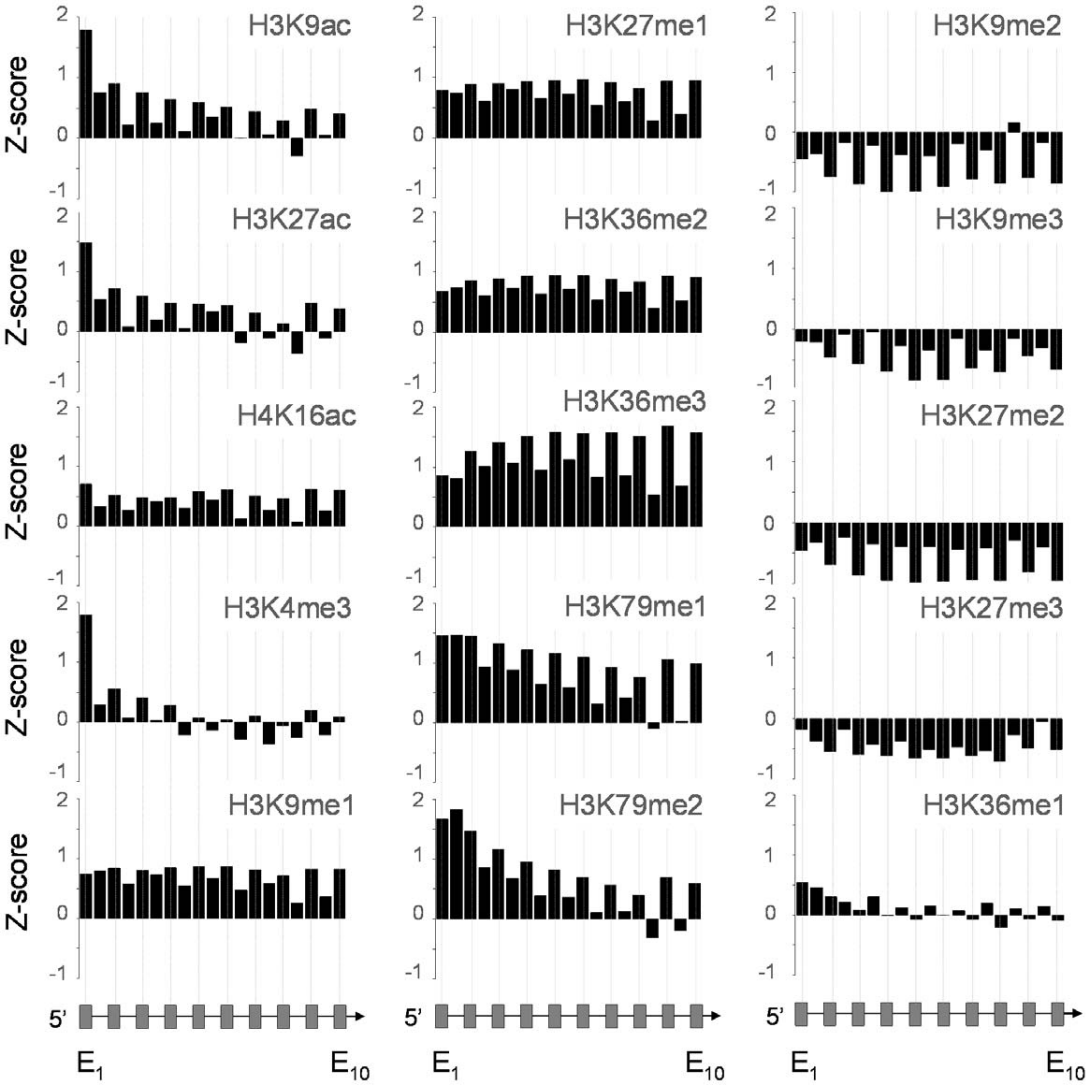
Histone modification patterns track exons and introns across gene bodies which is not accounted for by nucleosome distribution. Histograms show the mean levels of ChIP-chip enrichments (Z-scores) for 15 histone modifications spanning the first ten exons and nine introns of expressed consensus genes (n = 268, exons∶introns  = 1466∶551). Data is derived from ENCODE regions in the K562 and U937 cell lines and CD14+ primary monocytes. Datasets were normalized with the combined histone distribution profiles obtained for H2B and H3 in each cell line. Some of these modifications showed the most obvious exon marking over the first two 5′ exons (eg., H3K9ac and H3K4me3), while others showed differential enrichments across the majority of the first ten exons (eg., H3K27me1 and H3K36me3), apart from the first two. Repressive modifications H3K9me2/3 and H3K27me2/3 showed preferential depletion of exons, while H3K36me1 showed preferential enrichment of introns. Hypothetical gene structures are shown at the bottom of the figure. Median *P*-value obtained from bootstrapping for exons and introns across all 19 histone modifications tested in this study was <1.0×10^−15^. Median *P*-value obtained for pair-wise t-tests between adjacent exon-intron pairs (exon_2_ → exon_10_) for the data shown in the figure was 3.54×10^−5^.

We analyzed further the co-localization of these histone modifications with respect to features within gene bodies. Initially, we combined data from all three cell types, in order to observe generalized trends of histone modification patterns which may reflect fundamental features of eukaryotic cells. We constructed consensus exon-intron plots across the ten most 5′ exons (to represent 5′ ends and gene bodies) and across the five most 3′ exons (to represent 3′ ends) of genes. Thus, unlike previous studies, we examined histone modification levels across the entire lengths of exons and introns rather than exclusively around the exons and their most immediate 5′ and 3′ intronic sequences. This allowed us to assess whether, on average, differences in histone modification levels could discriminate exons from introns across their entire lengths. For transcriptionally active (i.e., expressed) genes, we found patterns of differential “marking” of exons when compared to introns for 15 of the 19 histone modifications (Figure 1; Supplementary Figure S6). The majority of these biases (seen for 10 out of 15 modifications) were evident as enrichments favoring exons. These included H3K36me3 which had previously been reported as preferentially marking exons [12], [19], [20], [21], [22], [23]. Others, such as H3K9me2, H3K9me3, H3K27me2 and H3K27me3 were evident as selective depletions across exons. One modification, H3K36me1, showed an enrichment bias favoring introns. Surprisingly, non-expressed genes also showed consistent patterns of exon-intron marking for five modifications with some modifications having enrichment biases favoring exons (e.g. H3K27me3) while others showed biases favoring introns (e.g. H3K9me2) (Supplementary Figures S7). At 3′ ends of expressed genes, the differential marking of exons and introns was maintained up to the penultimate exon, after which the differential was less obvious or absent (Supplementary Figure S7). For virtually all histone modifications, the marking biases we observed were evident both with and without nucleosome normalization (Figure 1; Supplementary Figures S6 and S7), demonstrating that underlying nucleosome distributions could not account for the differential marking levels we observed. Furthermore, in several instances, marking biases were accentuated only through nucleosome normalization (Figure 1 and Supplementary Figure S6, eg. H3K36me1; Supplementary Figure S7a and c, eg. H3K27ac), providing evidence that nucleosome distributions could mask underlying differences in histone modification levels if not taken into account.

Overall, these marking patterns (both normalized and unnormalized with respect to nucleosome distributions) showed high levels of statistical significance (see Figures 1, Supplementary Figures S6, S7) when compared with randomized datasets of histone modification enrichments and when comparing enrichments found on exons with those of introns - demonstrating that the marking patterns and biases were directed specifically at exons or introns within the genome. These distributions could not be accounted for by variations in GC-content [12] which may affect hybridization kinetics in ChIP-chip assays, since we observed histone modification biases both for exons and for introns (although exons generally have a higher GC-content than introns). Furthermore, nucleosome normalization would take into account any GC-content related effects. Thus, we attributed our results as indicative of a complex *bona fide* marking system of exon-intron structures defined by histone modification levels and not by nucleosome distributions, as reported previously [19], [20], [21], [22], [23].

The complexity of this marking system was further elaborated when we examined histone modification exon-intron marking biases for each of the three cell types independently (Supplementary Figure S8). We identified a high degree of variability in the marking repertoire of each cell type – with one or two cell types showing exon bias for a given histone modification, while the third cell type showing the opposite (i.e., intronic) or no exon-intron bias at all. Only eight modifications showed consistent biases across all three cell types and, even then, only showed these biases in the context of expression (i.e., either for expressed and/or non-expressed genes). For expressed genes in all three cell types, H3K36me3 was the only mark showing strong enrichment bias for exons, with H3K9me2, H3K9me3, H3K27me2 and H3K27me3 showing consistent exonic depletion. For non-expressed genes, H3K9me2 and H3K9me3 showed intronic enrichment, H3K27me3 showed exonic enrichment, while H3K36me1, H3K9ac and H3K18ac all showed intronic depletion. Cell-type specific differences in marking biases were not due to inherent differences between the epigenetic states of cell lines and primary cells, since K562 showed as many concordant marking biases with CD14+, as it shared with U937 (Supplementary Table S1).

### Variations in Nucleosomal Architecture Between Cell Types

**Figure 2:**
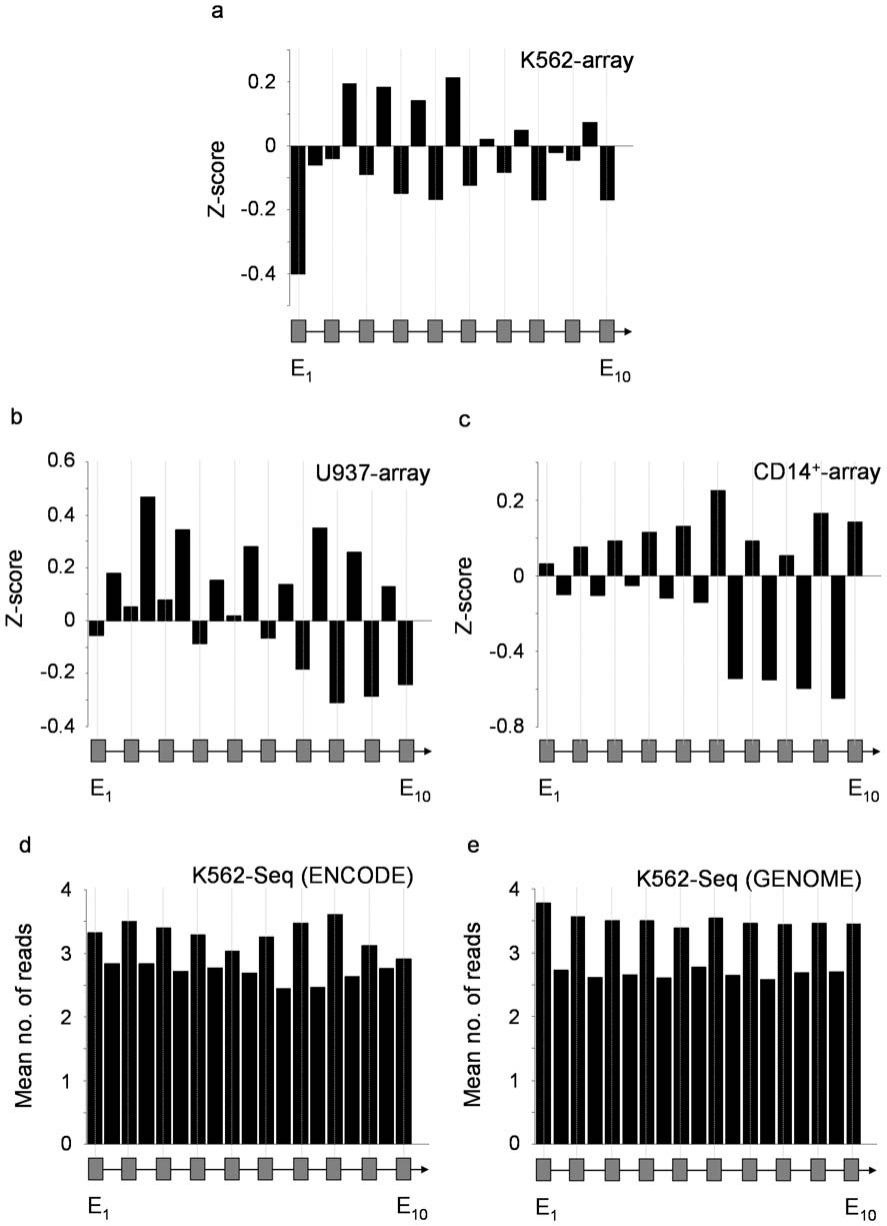
Nucleosome distribution patterns in three cell types display different biases with respect to exon-intron structures in gene bodies of expressed genes. Histograms show the mean levels of ChIP-chip enrichments (Z-scores) or mean number of reads (ChIP-seq) for histones spanning the first ten exons and nine introns of consensus expressed genes. **a.** K562 cell line using ChIP-chip (n = 76, exons∶introns  = 477∶187). **b.** U937 cell line using ChIP-chip (n = 88, exons∶introns  = 558∶219). **c.** CD14+ primary monocytes using ChIP-chip (n = 80, exons∶introns  = 493∶181). **d.** K562 cell line using ChIP-seq (n = 68, exons∶introns  = 465∶418). **e.** K562 cell line using ChIP-seq (n = 1184, exons∶introns  = 8095∶7500). Data was derived as the combined dataset for H2B and H3 across the ENCODE regions (panels a → d) or across the whole genome (panel e). Hypothetical gene structures are shown at the bottom of each panel of the figure. Median *P*-values obtained from bootstrapping for exons and introns were 1.77×10^−13^ (panel a), <1.0×10^−15^ (panel b), 5.93×10^−11^ (panel c), <1.0×10^−15^ (panel d) and <1.0×10^−15^ (panel e). Median *P*-values obtained for pair-wise t-tests between exons and introns (exon_2_ → exon_10_) were 4.14×10^−4^ (panel a), 1.87×10^−13^ (panel b), 2.33×10^−16^ (panel c), 3.21×10^−10^ (panel d) and <1.0×10^−15^ (panel e).

We further explored the underlying nucleosomal landscape across the three cell types to determine why our ChIP-chip histone modification patterns were not accounted for by nucleosome levels as shown in previous studies using ChIP-sequencing [19], [20], [22], [28]. For this analysis, we also performed ChIP-seq in the K562 cell line to determine whether we observed the same nucleosomal patterns with both ChIP-chip and ChIP-seq platforms. By examining nucleosomal levels across exons and introns of expressed genes, we observed striking variations in the three cell types analyzed by ChIP-chip (Figure 2). Both K562 and U937 displayed higher levels of nucleosomes in introns, while CD14+ cells showed higher levels across exons. This not only highlighted that different cell types may have different nucleosomal architectures, but reinforced that nucleosome distributions did not account for, and were often opposing, exon-intron marking by histone modifications (Supplementary Figure S8). Remarkably, in our analysis of nucleosome density in K562 using ChIP-seq, we saw a distinct bias in nucleosome distribution favoring exons which was in direct contrast to the patterns observed with ChIP-chi. This nucleosomal exon bias was seen for the set of expressed genes in ENCODE regions which we had analyzed by ChIP-chip, and also genome-wide for all expressed genes in K562. These results not only provide compelling evidence that ChIP-chip and ChIP-seq reveal different nucleosomal architectures, but also helped reconcile the differences in marking patterns which we observed with ChIP-chip from that which others observed with ChIP-seq [19], [20], [22], [28]. While ChIP-chip is likely to capture the entire chromatin *milieu* of cells, size-selection of ChIP-seq material may only capture a proportion of the information which is obtained from ChIP assays. This interpretation is supported by studies which have shown than sonication of cross-linked chromatin followed by massively-parallel sequencing of size-selected material, enriches for regions of high chromatin accessibility [29]. Such enrichment would also apply to sequencing of ChIP samples derived by either cross-linking followed by sonication, or by native ChIP using micrococcal nuclease digestion, as both procedures are unlikely to fragment the genome randomly prior to chromatin immunoprecipitation. Therefore, we believe that our ChIP-chip datasets accurately reflect marking of exons and introns (by both nucleosomes and histone modifications) in the cell types we analyzed.

### Evidence for the Combinatorial Nature of Exon-Intron Marking

It is thought that the co-occurrence of histone modifications on the same histone tails could function combinatorially as a “histone code” to regulate biological outcomes [1]. To provide evidence for the combinatorial nature of exon-intron marking, we addressed whether histone modifications could exist together on the same histone tails by performing sequential-ChIP on ENCODE microarrays (seq-ChIP-chip). We used permutations of histone modifications which showed concordant marking biases in K562 cells in either expressed or non-expressed genes (H3K36me3 followed by H3K27me1 - both favoring exons of expressed genes; H3K27me3 followed by H3K36me1 - both favoring exons of non-expressed genes). These combinations showed improved resolution of exon-intron marking after seq-ChIP-chip for either expressed or non-expressed genes which could not be accounted for by nucleosome distribution (Supplementary Figure S9 and S10). To provide evidence that our seq-ChIP-chip procedure resulted in enrichments coming from both ChIPs when assayed sequentially, we also performed two control seq-ChIP-chip experiments. The first (H3K27me1 followed by H3K36me3) showed that our seq-ChIP-chip could detect enrichments irrespective of the order that the ChIP assays were performed. The second was used to show that when two histone modifications with opposing types of tracking (one favoring exons and one favoring introns across expressed genes) were used in seq-ChIP-chip, the resultant sequential profiles showed an overall loss or reversal of tracking which was no longer statistically significant (Supplementary Figures S9 and S10). Our data suggests that there is combinatorial marking of exons with H3K27me1/H3K36me3 or H3K27me3/H3K36me1 within gene bodies of expressed or non-expressed genes respectively. However, whether these signatures occur together on the same histone H3 N-terminal tail, or on the same or closely spaced nucleosomes cannot be determined from our assays. Previous sequential-ChIP analysis had shown that a H3K27me3/H3K4me3 “bivalent” signature is likely to exist on the same histone tails at the 5′ ends of a subset of developmentally regulated genes which are either silent or have low levels of transcriptional activity [2], [30]. The exon marking signatures we describe here span entire gene bodies of either expressed or non-expressed genes, and may thus be typical signatures of all genes. Furthermore, these combinations provide a tantalizing clue that there may be a “combinatorial switch” - (H3K27me3 → H3K27me1 and H3K36me1 → H3K36me3), when a gene goes from being repressed to being active.

### Exon-Intron Marking Reflects Exon Usage

**Figure 3:**
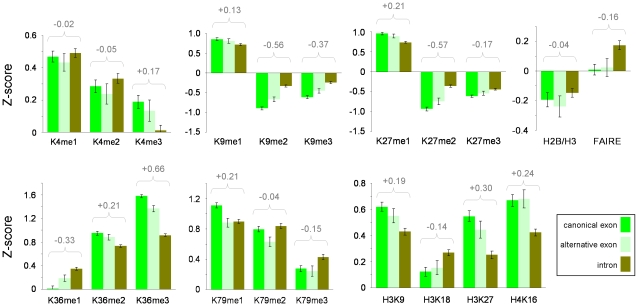
Histone modifications differentially mark canonical and alternatively-spliced exons and introns across bodies of expressed genes. Histograms show the mean levels (Z-scores) for histone modifications and histones (ChIP-chip enrichments) or chromatin accessibility (FAIRE) spanning typical canonical/alternatively-spliced exons and introns. Data was derived from gene bodies of expressed genes (n = 268, canonical exons:alternatively-spliced exons∶introns  = 2463∶523∶3036) in the K562 and U937 cell lines and CD14+ primary monocytes across the ENCODE regions. Histone distribution was based on the combined data for H2B and H3 in each cell type. Biases favoring either canonical exon or intron are summarized by the difference in Z-scores shown above each assay in grey. Positive (+) differences in Z-scores reflect exon biases, while negative (−) differences reflect intron biases. Error bars are 95% confidence intervals.

We next examined the possible functions of this histone modification marking system. H3K36me3 marking, among others, had previously been implicated in co-transcriptional splicing with enrichment biases showing a relationship with exon usage (i.e., favoring canonical exons rather than alternatively-spliced exons across expressed genes) [12], splice-site strength [19], [20], [23] or splice-site switching [24]. For all expressed and non-expressed genes identified in K562, U937 and CD14+ cells, we examined the mean enrichment levels of each of the 19 histone modifications across a total of 3761 canonical exons and compared them with the levels attributed to alternatively-spliced exons and introns (totals of 1064 and 4626 respectively). We considered that any biases at 5′ ends of coding sequences may be distinct from those across gene bodies, as they lie within close proximity to promoters which have unique histone modification signatures. Thus, we partitioned our datasets accordingly with the 5′-most 25% of gene lengths being considered separately from the remainder of gene bodies. We observed extensive exon usage biases within gene bodies (Figure 3, Supplementary Figure S11). In gene bodies of expressed genes, 13 histone modifications mirrored exon usage as either enrichment or depletion biases, depending on whether the marking favored exons or introns. In these cases, alternatively-spliced exons showed mean values which lay between those found for canonical exons and those for introns – as would be expected, given that only a proportion of alternatively-spliced exons would be used in the cell types we examined. Exon usage biases were further accentuated in sequential-ChIP-chip assays for combinations of H3K36me3 and H3K27me1 in the K562 cell line (Supplementary Figure S10). This data supports previous findings linking exon-intron marking by histone modifications with co-transcriptional splicing, although our evidence would suggest that at least 13 histone modifications are involved in this process (at least two of which may act in specific combinations on expressed genes). However, six modifications did not show alternatively-spliced exons having mean enrichments between those of canonical exons and introns for expressed genes (e.g. H3K79me1→3, H4K16ac). This, in addition to the data which showed exon or intron biases for histone modifications across non-expressed genes (Figure 1 and Figure 3) suggests that other features, apart from exon usage, are being marked across gene structures. Furthermore, given that cell-type specific exon-intron marking also exists (Supplementary Figure S8), the relationship between histone modifications and splicing may also have cell-type specific components.

### RNA Polymerase II Occupancy is Linked to Chromatin Accessibility and Histone Modification Patterns

Previous studies have linked Pol II occupancy with nucleosome positioning, suggesting that the nucleosome *per se*, may provide a barrier or “speed bump” which impedes Pol II movement across exons which have well-positioned nucleosomes [19], [31]. However, given that these studies were conducted using ChIP-seq, we considered that they may not provide a completely unbiased view of the role of chromatin architecture in regulating Pol II movement. We adopted a view that exon-intron marking by histone modifications, at least for expressed genes, may also be involved in regulating chromatin accessibility to facilitate the movement of Pol II across genes during transcription. Therefore, we examined whether other features of chromatin structure across expressed genes also displayed exon-intron marking which were concordant with histone modification patterns. We had already demonstrated that the densities of histones H2B and H3 (i.e., nucleosomes) were lower across exons than introns of expressed genes in both the K562 and U937 cell lines, but the patterns were reversed in CD14+ monocytes (Figure 2). FAIRE accessibility assays showed the highest levels of accessibility across introns of both expressed and non-expressed genes in all three cell types (Supplementary Figure S12). Therefore, the different nucleosome distributions in our three cell types could not explain the FAIRE patterns, confounding views that nucleosome patterns *per se* determine accessibility. However, eight histone modifications (H3K9me2, H3K9me3, H3K9ac, H3K27ac, H3K27me2, H3K27me3, H3K36me1, and H3K36me3) consistently showed differential exon-intron marking across all three cell types (see above and Supplementary Figure S8) which were concordant with the FAIRE profiles for either expressed or non-expressed genes. This pointed to these histone modifications being directly related to accessibility across gene bodies.

**Figure 4:**
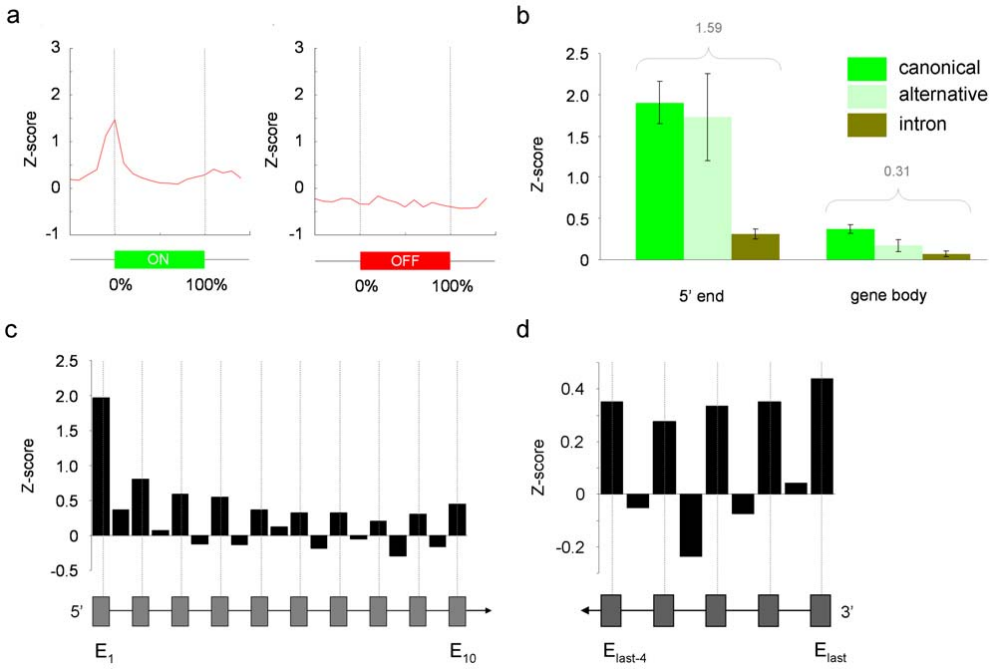
RNA polymerase II (Pol II) occupancy levels are increased at transcribed exons. **a.** Pol II levels across consensus expressed (“ON”) (n = 245) and non-expressed (“OFF”) genes (n = 115). **b.** Histograms show levels of Pol II at 5′ ends and across gene bodies with respect to canonical/alternatively-spliced exons and introns of expressed genes [n = 181, canonical exon:alternatively-spliced exon∶intron  = 330∶151∶496 (5′ ends) or 1705/371/2110 (gene bodies)]. Biases favoring either canonical exon or intron are summarized by the difference in Z-scores shown above each assay in grey. Positive (+) differences in Z-scores reflect exon biases, while negative (−) differences reflect intron biases. Error bars are 95% confidence intervals. **c.** Exon-intron tracking of Pol II across the first ten exons and nine introns of consensus expressed genes (n = 181, exon∶introns  = 980∶376) (hypothetical gene structure shown below panel). **d**. Exon-intron tracking of Pol II across last 5 exons and 4 introns of consensus expressed genes (n = 181, exon∶introns  = 563∶148) (hypothetical gene structure shown below panel). Median *P*-values obtained from bootstrapping for exons and introns in **c** and **d** were both <1.0×10^−15^. Median *P-*values obtained for pair-wise t-tests between adjacent exon-intron pairs in data from **c** (exon_2_ → exon_10_) and in **d** (exons_last-4_ → exon_last_) were 5.06×10^−6^ and 6.30×10^−4^ respectively. In all panels, Pol II ChIP-chip enrichments across ENCODE genes in the K562 and U937 cell lines are expressed as mean Z-scores.

This suggested to us that the combinatorial effect of differential histone modification load across gene bodies may result in an overall more compact chromatin configuration across exons of expressed genes. From this, we predicted that although K562 and U937 both have higher nucleosome densities in introns than in exons, Pol II occupancy across exons would be higher than across introns. Our prediction was substantiated by observing significant exon bias in Pol II occupancy across expressed genes in K562 and U937 when we performed ChIP-chip assays with an antibody which recognized both initiating and elongating forms of Pol II (Figure 4 and Supplementary Figure S13). This bias reflected exon usage and showed highest levels of occupancy at both 5′ and 3′ ends of genes. Unlike histone modification profiles which differentially marked exon-intron structure across genes up to the penultimate exon (Supplementary Figure S6), marking by Pol II appeared to continue right up to the last exon.

Taken together, our data points towards a close relationship between chromatin accessibility/compaction, histone modifications and the movement or “pausing” [32] of Pol II across exon-intron structures during transcriptional elongation. In turn, Pol II exon “pausing” may affect exon usage during co-transcriptional splicing. In support of our interpretations, data from a variety of sources suggests that histone modifications [12], [24], [33], histone deacetylase and chromatin modelling activity [34], [35], [36] and the speed of Pol II movement or pausing [37], [38], [39] may facilitate splice-site selection in alternatively-spliced mRNAs. This would imply that the role of histone modifications in co-transcriptional splicing is not direct, as suggested previously [12], [24]. While it is possible that well-positioned nucleosomes across exons are also related to Pol II movement [19], cues received by Pol II from histone modifications on these nucleosomes may be the critical factor in controlling both Pol II movement and the recruitment of splicing factors. Our data is consistent with histone modifications having both of these roles, as has been proposed by other investigators [31]. Furthermore, differences in marking between histone modifications and Pol II at the 3′ ends of expressed genes suggest that there are other features involved in pausing during polyadenylation [40], [41] that we have not yet examined.

### Exon-Intron Marking is Dependent on Rates of Transcription

**Figure 5:**
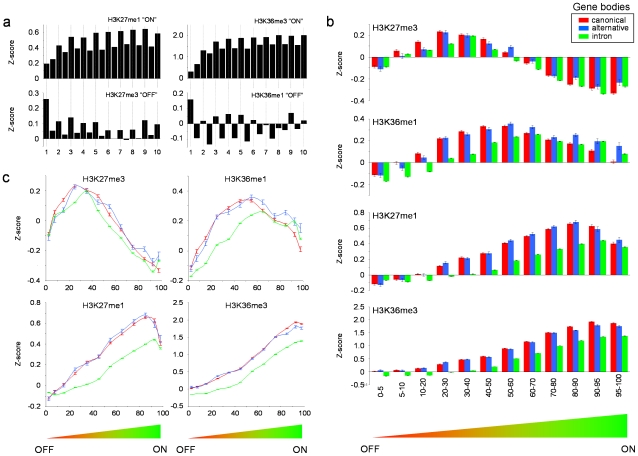
Genome-wide histone modifications patterns track exon-intron structures in gene bodies according to levels of gene expression or repression. **a.** Histograms show the level of four histone modifications across the first ten exons and nine introns of consensus genes expressed (“ON”) (n = 1845, exons∶introns  = 12763∶10853) or non-expressed (“OFF”) (n = 1657, exon∶introns  = 10911∶ 9194). Exon numbering is at the bottom of the panel. Median *P*-value obtained from bootstrapping for exons and introns for all four modifications were <1.0×10^−15^ (“ON”) and <1.0×10^−15^ (“OFF”). Median *P*-values obtained for pair-wise t-tests between adjacent exon-intron pairs (exon_2_ → exon_10_) for the data shown in the figure were 1.01×10^−49^ (“ON”) and 6.48×10^−09^ (“OFF”). **b.** Histograms show relationships between four histone modifications and canonical/alternatively-spliced exons, and introns across gene bodies as a function of expression levels (percentile rankings on the x axes). Genes (n = 9921, canonical exons:alternatively-spliced exons∶intron  = 70470∶20733∶91613) were ranked into 12 bins according to expression level (percentile rankings on the x axes). Error bars are 95% confidence intervals. **c.** Line graphs of the levels of the four histone modifications from **b** as a function of expression level (percentile rankings on the x axes) and exon/intron structure (red  =  canonical exons, blue  =  alternative exons and green  =  introns). Error bars are 95% confidence levels. In all panels, ChIP-chip enrichments obtained from genome-wide analysis of the K562 cell line are expressed as mean Z-scores.

We were able to determine that the exon-intron marking patterns we had observed across the ENCODE regions were fundamental features of all human genes by performing ChIP-chip using Affymetrix GeneChIP® whole genome tiling arrays. These studies were performed in the K562 cell line for four modifications (H3K27me1, H3K27me3, H3K36me1 and H3K36me3) which typified the kind of exon or intron marking biases we had observed across the ENCODE regions, and which appeared to be distributed as combinatorial signatures in either expressed or non-expressed genes (see Results above and Supplementary Figures S9 and S10). We examined histone modification patterns across the gene structures of 9921 genes for which we had consistent expression data, and gene/transcript information available from ENSEMBL (see Materials and Methods). For both expressed and non-expressed genes, we observed genome-wide patterns of exon-intron marking that were in agreement with those we obtained across the ENCODE regions (Figure 5A, Supplementary Figures S14, S15). We did, however, observe that similarities between the ENCODE and whole genome datasets were most evident when we explored different binning strategies for expressed and non-expressed genes. For example, for H3K27me1, the top 25% of expressed genes in genome-wide analysis showed patterns similar to the ENCODE regions (Figure 1 and Figure 5a), whilst only the bottom 10% of non-expressed genes genome-wide showed the same patterns obtained for ENCODE. This suggested that different rates of transcription reflected in steady-state expression levels may have a bearing on the marking system. By constructing 12 intervals of modification data based on the Robust Multichip Average (RMA) values of all 9921 genes, we uncovered striking differential exon/intron marking as a function of expression level (Figure 5b, c) displaying a number of prominent features.

Firstly, the most repressed/silent genes (the first decile in Figure 5a) were not appreciably enriched for these four histone marks. Secondly, substantial enrichments for the repressive H3K27me3 were the first to appear in the second and third deciles, followed thereafter by the other three modifications as “waves” of increasing and then decreasing enrichment levels, in the order H3K36me1, H3K27me1 and H3K36me3. Genes within deciles 1 to 4, however, were still transcriptionally inactive, as we could not detect appreciable levels of Pol II binding for 24 genes assayed by ChIP-chip which were partitioned into these deciles (data not shown). Thirdly, exons were preferentially loaded with modifications at lower levels of expression, with introns only showing similar levels of enrichments at higher levels of expression (shown as an intron “lag” in Figure 5c). The marking biases at canonical or alternatively-spliced exons differed as a function of expression level, and in some instances, showing higher enrichments for alternatively-spliced exons than for canonical ones (e.g. H3K36me1 and H3K27me1), again suggesting that exon usage *per se* was not the only factor that distinguished them. Fourthly, at the very highest levels of expression (the top 5-10% of RMA values, decile 10), the marking biases and enrichment levels of all four modifications showed distinct shifts away from those seen in the previous deciles. More specifically, (i) marking biases for both H3K36me1 and H3K27me3 switched from favoring exons, to favoring introns, and (ii) the differentials between canonical exons, alternative exons and introns for all four modifications changed. We further refined the nature of some of these shifts by examining the predicted level of inclusion of alternatively-spliced exons in gene transcripts expressed at high levels. H3K36me3 levels showed enrichments which reflected exon usage, with frequently included alternatively-spliced exons having higher modification levels than infrequently included ones (Supplementary Figure S16). Surprisingly, H3K36me1, H3K27me1 and H3K27me3 all showed significantly higher levels of enrichments for less frequently included exons, supporting the idea that exon exclusion, rather than exon inclusion or usage, was also a feature of the marking system. This last feature points to a specialized need to further regulate chromatin structure and Pol II movement at very high rates of transcription – this may be required to maintain the fidelity of transcriptional elongation or the splicing process.

### Exon-Intron “Priming” in the Absence of Transcription

**Figure 6:**
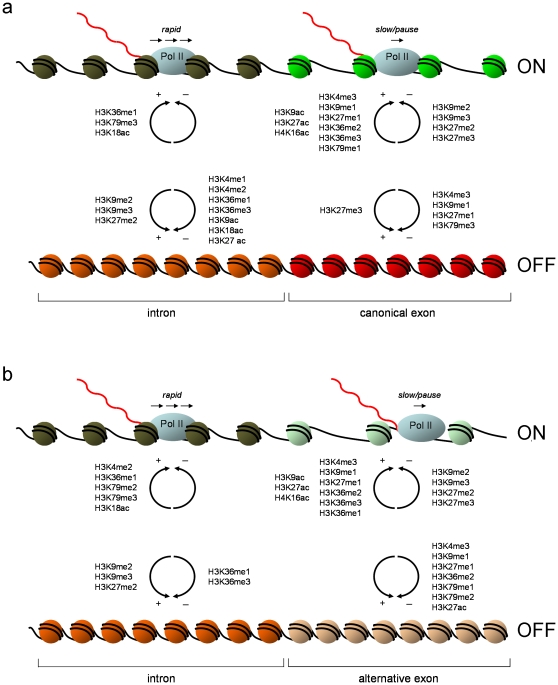
Schematic model of the relationships between histone modifications and exon-intron structures across expressed and non-expressed/silent genes. Model is based on relationships observed for both ENCODE and whole genome datasets described in the text. Circular arrows reflect statistically significant increases (+) or decreases (−) in histone modification levels (shown either side of the arrows) observed when comparing a typical intron and a typical exon (either canonical or alternative) in either the expressed (“ON”) or non-expressed (“OFF”) state. Relative distances between nucleosomes are based on histone density data. Predicted Pol II movement is also shown. Transcribed mRNA is shown in red. **a.** Canonical exon versus intron. **b.** Alternatively-spliced exon versus intron.

We propose that the “waves” of differential exon-intron marking with histone modifications described above could model the temporal events that occur when genes go from being transcriptionally repressed/silent to being transcriptionally active. For individual genes which were differentially expressed between the three cell types we examined, we confirmed that changes in expression from non-expressed to expressed (“OFF” to “ON”) or from low expression to high expression were accompanied by the changes predicted for these four histone modifications (Supplementary Figure S17). All of our data, taken together, suggests that such a model has both transcriptionally-independent (“priming”) and transcriptionally-dependent phases (Figure 6). Furthermore, in both of these phases, histone modification marking distinguishes canonical exons from alternatively-spliced exons, both of which are seen as distinct from introns. Based on our whole genome analysis, “priming” of exons and introns is likely to involve H3K27me3 and H3K36me1, both of which appear loaded onto histones when genes are silent or at low levels of gene expression. These modifications are removed in favor of H3K27me1 and H3K36me3 as expression levels increase. This would indicate that the switch from the tri- to mono-methyl state for H3K27 and the switch from the mono- to tri-methyl state for H3K36 may be involved in the transition from “priming” to transcription, or from low-level to high-level transcription. This idea is also supported by our sequential-ChIP-chip which showed that the combination H3K27me3/H3K36me1 is found on non-expressed genes, whereas expressed genes have H3K36me3/H3K27me1 (Supplementary Figures S9 and S10). The presence of exon-intron marking for H3K9me2 or 3 and H3K27me2 across non-expressed genes (Figures 1 and 3) argues that a variety of repressive marks are also involved in the “priming” process. Noticeably, these latter marks show biases favoring introns, which is in contrast to the exon enrichment bias seen with H3K27me3. Therefore, the “priming” process may be capable of distinguishing the full complement of coding features (both exons and introns) in the absence of transcription. We speculate that “priming” may provide a transcription-ready template of exon-intron structures, and that this template serves to facilitate subsequent phases of marking and chromatin re-organization during transcription. This model, which is consistent with data from one other study [23], confounds previous views that histone modification patterns across gene bodies are found only on transcribed genes [12], [19], [20] and points to exon-intron marking as a constitutive feature of eukaryotic genes irrespective of transcriptional activity.

## Discussion

There is a growing body of evidence linking chromatin structure and function, exon-intron organization and co-transcriptional splicing/mRNA processing (reviewed in [31], [42]). Our study provides several lines of evidence pointing to histone modifications as having roles in determining chromatin accessibility, Pol II movement and co-transcriptional mRNA processing at a global level through exon-intron marking. Our results are in striking contrast to previous reports which showed that histone modification exon-intron marking patterns were merely a reflection of nucleosomal distribution, with well-positioned nucleosomes on exons accounting for apparent histone modification biases [19], [20], [21], [22]. Having accounted for differences in nucleosome distribution, the patterns of histone modifications we observed can only be attributable to active cellular mechanisms (i.e., the action of histone modifiers) which overlay the nucleosomal architecture with modifications on specific nucleosomes located within exons or within introns. Furthermore, while other studies had observed exon marking across expressed genes by analyzing a single cell type, our data points to aspects of this marking as being cell-type specific, combinatorial, and related to levels of transcription – with even untranscribed genes having exon-intron marking biases. We also provide evidence that this marking system is not simply mirroring exon usage, as some modifications track infrequently used alternative exons. These observations have not been fully described previously.

What remains to be determined are how each histone modification contributes to chromatin configurations and the control of Pol II movement, which trans-acting factors lay down the marks, and whether the marks facilitate recruitment of additional factors during the three phases of transcription, and during co-transcriptional splicing. Whether aspects of this marking system function in a truly combinatorial fashion must be explored further. Given our evidence which shows exons are “primed” with histone marks prior to their transcription, how this “priming” is laid down in the absence of Pol II, and whether it is developmentally regulated are particularly interesting areas to explore. The findings of the present study confound previous views on chromatin and splicing and provide the starting point for several new avenues of investigation.

## Materials and Methods

### ENCODE Tiling Array

The construction of the PCR product Sanger Institute ENCODE array is described in detail elsewhere [4]. This array was further supplemented with a 256 kb tiling path of the human SCL locus for which a detailed analysis of histone modifications had previously been determined [43]. The preparation of amplicons and arrays are also described at www.sanger.ac.uk/Projects/Microarrays/arraylab/methods.shtml.

### Cell Sources

Human cell lines K562 [44] and U937 [45] were cultured in DMEM, 9% fetal calf serum, 1% penicillin-streptomycin and 2 mM L-glutamine and in RPMI 1640, 18% fetal calf serum, 1% penicillin-streptomycin and 2 mM L-glutamine respectively. Human CD14+ monocytes were isolated from peripheral blood obtained from a subject of the Cambridge BioResource, a collection of 4000 pseudo-anonymized healthy blood donors that has been established by the Cambridge Biomedical Research Centre in collaboration with NHS Blood and Transplant, for use in genotype-phenotype association studies. The study was approved by the Cambridgeshire 1 Research Ethics Committee. CD14+ monocytes were purified using the RoboSep fully automated cell separator (Stem Cell Technologies Inc) according to manufacturer's instructions. Purity of CD14+ cells was determined to be greater than 98% by FACS [46].

### Antibody Specificity Determination

ChIP antibodies and pre-immune antisera controls used in this study are described in Supplementary Table S2. Dot blots for dilution series of histone peptides (5–100 ng/µl in 20 µM Tris-HCl, pH 7.5) containing modified histone residues or unmodified histone control peptides (Supplementary Table S2) were prepared by vacuum-blotting (Bio-Dot, BIO-RAD) using the Immobilon-P^SQ^ or Immobilon-P membranes (Millipore). Dot blots were hybridized using standard procedures detailed elsewhere [47]. Signal intensities of hybridization signals on ECL Hyperfilm™ (GE Healthcare) were obtained and the data summarized in Supplementary Figure S18.

### Chromatin Immunoprecipitation (ChIP) and Sequential ChIP

ChIP was performed as described elsewhere [4], [48] using varying cross-linking conditions depending on the assay (conditions available upon request). 8–10 µg of each antibody (Supplementary Table S2) were used in ChIP assays. Sequential ChIP (Seq-ChIP) was performed according to the protocol found in Supplementary Protocol S.1. Mock IP controls using the relevant pre-immune antisera were performed for ChIP and for both rounds of Seq-ChIP. Three bioreplicates were performed for each ChIP assay.

### ChIP-chip Labelling, Microarray Hybridization and Quantitation

Fluorescently-labelled DNA samples were prepared from unamplified input/ChIP/FAIRE DNAs and hybridized onto the ENCODE PCR product tiling array for 45 hours using an automated hybridization station (HS 4800™, TECAN) as described elsewhere [4]. Microarrays were scanned using a ScanArray 4000 XL (Perkin Elmer). Mean spot intensities from images were quantified using ProScanArray® Express (Perkin-Elmer) with background subtraction. Spots affected by dust were manually flagged as “not found” and excluded from subsequent analysis. These data were submitted to ArrayExpress (accession no. E-MTAB-334).

### Whole Genome ChIP-chip Analysis

Chromatin immunoprecipitated DNA samples and the input (control) sample (see above) were amplified with a version of the REPLI-g FFPE Kit (Qiagen) according to the manufacturer's protocol. 20 µg of amplified DNA of each sample was fragmented in 1×One-Phor-All Buffer plus (GE Healthcare) using 1∶50 dilution of DNAse I (Epicentre) for 9 minutes at 37°C followed by heat inactivation at 100°C for 10 minutes and snap cooling on ice for 2 minutes. The fragmented DNA was checked on an agarose gel to ensure that the main DNA band was below 100 bp. The fragmented DNA was end-labeled using the GeneChip WT Double-Stranded DNA Terminal Labeling Kit (Affymetrix) according to the manufacturer's protocol. 18 µg of amplified samples were hybridized to the GeneChip® Human Tiling 2.0R Array Set (Affymetrix) and washed, stained and scanned following the manufacturer's protocols. The scanned output files were analyzed with Tiling Analysis Software version1.1 (Affymetrix, Santa Clara, CA, USA). Probes were mapped to human chromosomes according to NCBIv35 (hg17) genome assembly. The samples (ChIP and genomic input samples) were normalized together by quartile normalization using a linear intensity scale. Two-sample analysis using only data from the perfect matches with bandwidth 40 was applied to the sample to determine the ChIP enrichment at each probe position. These data were submitted to ArrayExpress (accession no. E-MTAB-336).

### ChIP-sequencing

ChIP assays for histones H2B and H3 were performed in the K562 cell line as described above. Samples were prepared for next-generation sequencing using the Illumina ChIP-seq kit (IP-102-1001) according to manufacturer's instructions. Samples were sequenced on the Genome Analyzer IIa (Illumina).

### Gene Expression Analysis

We prepared total RNA from three bioreplicates of K562, U937 and CD14^+^ cells using TRIZOL reagent as described at http://www.sanger.ac.uk/Projects/Microarrays/arraylab/protocol1.pdf. RNA isolated from CD14+ monocytes was purified further using the RNeasy MinElute Cleanup Kit following the manufacturers' instructions (Qiagen). Each purified RNA sample was assessed for quality and integrity using the 2100 Bioanalzyer (Agilent) following the manufacturers' instructions. Transcriptional activity in K562 and U937 was determined by hybridizing samples to the Affymetrix U133 plus 2.0 gene expression microarray and also to the Sanger ENCODE array. For Affymetrix GeneChip analysis, samples were prepared according to the Affymetrix GeneChip Expression Analysis Manual (Affymetrix) using 5 µg of total RNA. For Sanger ENCODE array analysis, labelled samples were prepared by oligo-dT priming or random priming as described at http://www.sanger.ac.uk/Projects/Microarrays/arraylab/protocol5.pdf. RNA samples (labelled with Cy3) and genomic DNA (labelled with Cy5) from the same cell source were hybridized as for ChIP-chip analysis. Expression analysis of CD14+ monocytes was performed using Illumina Human-6 v2 BeadChips (Illumina Inc. San Diego, USA) [46]. Total RNA (500 ng) from each sample was amplified and labelled by *in vitro* transcription according to the manufacturer's instructions (Illumina TotalPrep RNA amplification kit, Ambion, Texas, USA). 1500 ng of biotinylated aRNA were hybridized, and the BeadChips washed, stained and scanned following the manufacturer's instructions. For each cell type analyzed, three biological replicates were performed across the relevant microarray platforms. Affymetrix GeneChip and Illumina BeadChIP data were submitted to ArrayExpress [accession nos. E-MTAB-335 (K562 and U937) and E-TABM-633 (CD14+ monocytes)].

For Sanger ENCODE arrays, overall gene expression levels across all three bioreplicates were computed as the average (mean) of the random-primed and oligodT Z-scored data mapping to the last 2000 bases average for oligo dT-primed and random primed normalized Cy3/Cy5 ratios for all tiles encompassing a gene. Whole Affymetrix expression data were analyzed with Bioconductor (http://bioconductor.org/), normalized and transformed using the MAS5 and RMA methods in the affy package (http://bioconductor.org/packages/bioc/html/affy.html). Similarly, Illumina data was analyzed by RMA using the lumi (http://bioconductor.org/packages/bioc/html/lumi.html) package. To determine the overall expression status of genes in ENCODE regions based on Affymetrix, Sanger ENCODE array and Illumina analysis, expression levels of 618 annotated genes were binned into quartiles based on RMA (Affymetrix) or Z-scores (Sanger ENCODE arrays). Genes considered expressed (“ON”) and non-expressed (“OFF”) were those that were found in the top 2 quartiles of ranked genes and the bottom quartile of ranked genes respectively. Genes expressed in K562 and U937 which were found in the intersection of quartile bins for both Affymetrix and Sanger ENCODE array analysis (Supplementary Table S3, S4, S5, S6) were used further. CD14+ monocyte gene ranking was performed using RMA of Illumina data only and “ON/OFF” states determined using the same quartile strategy as above (Supplementary Table S7–S8). For interpretation of whole genome ChIP-chip datasets in the K562 cell line in the context of gene expression, data was considered for only those genes consistently called either absent or present by MAS5 on Affymetrix Expression GeneChips and which also had ENSEMBL identifiers (Supplementary Table S9). These genes were then ranked by RMA values. Expressed and non-expressed genes were considered to be those in the top 25% and bottom 25% of RMA values respectively, unless specified in the text or figures.

### Computational Analyses of ChIP-chip Data

#### (i) Pre-processing and Normalization

Pre-processed data were created from raw enrichment data from three bioreplicate hybridizations. The ratios of the background corrected ChIP signal divided by the background corrected input signal, both globally normalized to the median ratio, were used for all ChIP-chip analyses. Ratios of duplicated spots were averaged. Ratios of spots defined as “not found” and ratios with a value below zero were excluded from the analysis and also excluded from the final composite median data. In addition, histone modification ChIP-chip datasets were normalized with respect to histone density (based on averaged H3 and H2B levels) and with rabbit IgG control datasets by dividing the final composite median data for each ChIP-chip assay with histone density or IgG data on a tile by tile basis. Pol II ChIP-chip datasets were normalized with mouse IgG control datasets. Final normalized datasets were used to create 3 datasets used in the analyses: (i) centred enrichment data, (ii) log2 centred enrichment data, and (iii) log2 centred Z-scored enrichment data (log2 centred data were divided by the standard deviation of the entire dataset). For Affymetrix GeneChip® tiling array experiments, the scanned output files were analyzed with Tiling Analysis Software version1.1 (Affymetrix, Santa Clara, CA, USA). The samples (ChIP and genomic input samples) were normalized together by quartile normalization using a linear intensity scale. Two-sample analysis using only data from the perfect matches with bandwidth 40 was applied to the sample to determine the ChIP enrichment at each probe position. All data were stored and analyzed on NCBI human genome build 35 (hg17).

#### (ii) Sequential ChIP-chip (Seq-ChIP-chip)

For sequential-ChIP (ChIP for histone modifications in both rounds) and sequential-ChIP controls (ChIP for histone modification in first round followed by ChIP for rabbit IgG in the second round), signal intensities in both channels were normalized independently and averaged for bioreplicates (based the median input signals), and then normalized between experimental and control datasets. The signals from each array element attributed to the sequential-ChIP experimental datasets were obtained by subtracting signal for each sequential ChIP control from the sequential-ChIP values in the ChIP channel. The enrichment ratio for sequential-ChIP was then determined relative to the normalized input channel. The ratios were then median centred, normalized with respect to histone density and rabbit antisera control datasets, and transformed to log2 centred Z-scored enrichment data as described above.

#### (iii) ChIP-sequencing

36 bp reads were aligned to the NCBI human genome build 36 (hg18) using the Burrows-Wheeler alignment algorithm Bowtie [49]. 14 040 928 (H2B) and 14 896 571 (H3) unique sequence reads were aligned unambiguously to the human genome. Aligned sequencing reads were filed into 200 bp “bins”.

#### (iv) Analysis of Histone Modification Enrichments and Gene Features

Normalized ChIP-chip and Seq-ChIP-chip data described above was viewed within the UCSC genome browser as formatted wiggle tracks (http://genome.ucsc.edu/goldenPath/help/wiggle.html) permitting the visualization of continuous-valued data in the context of annotated genome features. Modified histone behaviours in the context of composite genes were plotted using the R (http://www.r-project.org/) or Python programming language. Consensus histone modification profiles for subsets of “ON/OFF” genes were visualized based on expression levels as described above.

#### (v) ChIP-chip and ChIP-seq Levels Across Exons and Introns

The log2 centred Z-scored ChIP-chip and Seq-ChIP-chip enrichment data for 19 histone modifications, five Seq-chip combinations, histone density, FAIRE and Pol II were determined across exon-intron gene structures for the ENCODE datasets. For whole genome analysis, the log2 centred Z-scored enrichment data for H3K27me1, H3K27me3, H3K36me1, H3K36me3 were determined. For the ENCODE datasets, genes that encompassed at least 6 kb of genomic sequence and containing three or more exons were analyzed (see below). For whole genome analysis, in certain situations, only the three exon constraint was used. Exon (both canonical and alternative) and intron coordinates for genes with Ensembl IDs were downloaded from the Ensembl database (http://www.ensembl.org/) and associated with experimental datasets. Canonical exons were defined as those exons present in every annotated ENSEMBL mRNA transcript for a given ENSEMBL gene. Alternative exons were defined as those exons which were found in some, but not all, mRNA transcripts for a given ENSEMBL gene. Exons (both canonical and alternative) and introns were binned according to their position within the transcript. Histograms of histone modification and Pol II behaviours across consensus “ON” and “OFF” gene structures (first ten exons and nine introns) were derived using mean ChIP-chip or Seq-ChIP-chip enrichment/depletion values for canonical exons and introns. The gene structure at 3′ ends (last five exons and four introns) were analyzed in a similar fashion. The mean ChIP-chip enrichment/depletion values were also determined for canonical and alternative exons, and for introns (with 95% confidence intervals). For this analysis, 5′ ends of genes were considered to be the 5′-most 25% of the gene, whilst gene bodies were considered to be the remaining 75% of the gene. Sequences containing overlap between canonical and alternative exons were excluded from analysis. Genomic “bins” containing sequence reads from ChIP-seq datasets for H2B and H3 in K562 cells, were assigned to exons and introns in a similar fashion.

A randomization strategy (bootstrapping) was used to determine the statistical significance of ChIP-chip and ChIP-seq distributions across exons and introns. The genomic co-ordinates of microarray tiles were randomized within any single dataset 100 times to generate 100 random datasets across the ENCODE regions (or whole genome where appropriate). This effectively assigned datapoints, normally assigned to exons and introns, to random genomic co-ordinates. Mean ratios were calculated for exons and introns of consensus “ON” and “OFF” gene structures for the experimental and random datasets based on the correct annotated co-ordinates of exons and introns. Mean ratios from the experimental dataset were also compared to the population of randomized values to determine whether the mean ratios obtained in the experimental dataset could have occurred by chance in the randomized datasets and significance levels (*P*-values) were assigned. For ChIP-seq, 100 randomized datasets were generated for equivalent numbers of reads for both H2B and H3 (see above). In other words, 36 bp “read” co-ordinates were assigned randomly to each randomized dataset. Randomized datasets were filed into 200 bp “bins” as described for the experimental datasets (see above). Mean read levels for both exons and introns of expressed “ON” genes were determined for both the experimental and randomized datasets and *P*-values were assigned. Two-tailed t-tests were also performed for pairwise comparisons of histone modification or Pol II levels across exons (canonical) and introns in consensus gene structures (for gene structures from exons 2 → 10). Similarly, bootstrapping and t-tests were performed at the 3′ end of genes (for the last 5 exons and 4 introns) for ChIP-chip datasets.

## Supporting Information

Protocol S1 Protocol for Sequential-ChIP (Seq-ChIP) used in this study. All reagents (including suppliers and catalogue numbers) used for Seq-Chip assays are shown at the top of the protocol. For details of hybridization of these samples to Sanger Institute tiling microarrays, refer to our previous publications^1,2^ 1. Koch, C.M. *et al*. The landscape of histone modifications across 1% of the human genome in five human cell lines. *Genome Res* **17**, 691-707 (2007). 2. Bruce, A.W., Lopez-Contreras, A., Flicek, P., Down, T.A., Dhami, P., Dillon, S.C., Koch, C.M., Langford, C.F., Dunham, I., Andrews, R.M. and Vetrie, D. Functional diversity for REST (NRSF) is defined by in vivo binding affinity hierachies at the DNA sequence level. *Genome Res* **19**, 994-1005 (2009).
(2.28 MB DOC)

Figure S1 Histone modification patterns for expressed and non-expressed genes across the ENCODE regions in the K562 and U937 cell lines and CD14+ monocytes. a. Consensus gene plots for 19 histone modifications across expressed (ON) genes (n = 366). b. Consensus gene plots for 19 histone modifications across non-expressed (OFF) genes (n = 167). ChIP-chip enrichment levels in both panels are expressed as mean Z-scores. Proportional gene length and flanking regions are shown on the x axis as percentages (%). Color key to modifications depicted in each panel are shown to the right of the figure. Some modifications showed strong association with 5′ ends (i.e., promoters) or with gene bodies of actively transcribed genes (with either a 5′ or 3′ bias). Other modifications showed depletions across gene bodies of expressed genes. Consensus plots for non-expressed genes exhibited the typical hallmarks of H3K27me3 and H3K9me2 enrichments.
(1.14 MB TIF)

Figure S2 Histone modification patterns for expressed and non-expressed genes across the ENCODE regions in the K562 cell line. a. Consensus gene plots for 19 histone modifications across expressed (ON) genes (n = 111). b. Consensus gene plots for 19 histone modifications across non-expressed (OFF) genes (n = 53). ChIP-chip enrichment levels in both panels are expressed as mean Z-scores. Proportional gene length and flanking regions are shown on the x axis as percentages (%). Color key to modifications depicted in each panel are shown to the right of the figure. Trends were as described in Supplementary Figure S1.
(1.16 MB TIF)

Figure S3 Histone modification patterns for expressed and non-expressed genes across the ENCODE regions in the U937 cell line. a. Consensus gene plots for 19 histone modifications across expressed (ON) genes (n = 134). b. Consensus gene plots for 19 histone modifications across non-expressed (OFF) genes (n = 62). ChIP-chip enrichment levels in both panels are expressed as mean Z-scores. Proportional gene length and flanking regions are shown on the x axis as percentages (%). Color key to modifications depicted in each panel are shown to the right of the figure. Trends were as described in Supplementary Figure S1.
(1.15 MB TIF)

Figure S4 Histone modification patterns for expressed and non-expressed genes across the ENCODE regions in CD14+ monocytes. a. Consensus gene plots for 19 histone modifications across expressed (ON) genes (n = 121). b. Consensus gene plots for 19 histone modifications across non-expressed (OFF) genes (n = 52). ChIP-chip enrichment levels in both panels are expressed as mean Z-scores. Proportional gene length and flanking regions are shown on the x axis as percentages (%). Color key to modifications depicted in each panel are shown to the right of the figure. Trends were as described in Supplementary Figure S1.
(1.16 MB TIF)

Figure S5 Chromatin accessibility (FAIRE) and histone density patterns (H2B/H3) for expressed and non-expressed genes across the ENCODE regions in the K562 and U937 cell lines and CD14+ monocytes. a. Consensus gene plots across expressed (ON) genes in all three cell types [ALL (n = 366), K562 (n = 111), U937 (n = 134), and CD14+ monocytes (n = 121)]. b. Consensus gene plots across non-expressed (OFF) genes in all three cell types [ALL (n = 167), K562 (n = 53), U937 (n = 62), and CD14+ monocytes (n = 52)]. ChIP-chip enrichment levels in both panels are expressed as mean Z-scores. Proportional gene length and flanking regions are shown on the x axis as percentages (%). Color key to FAIRE and histone density assays in each panel are shown to the right of the figure.
(0.88 MB TIF)

Figure S6 Histone modification patterns track exons and introns across gene bodies without accounting for nucleosome distribution. Histograms show the mean levels of ChIP-chip enrichments (Z-scores) for 15 histone modifications spanning the first ten exons and nine introns of expressed consensus genes (n = 268, exons∶introns  = 1466∶551). Data is derived from ENCODE regions in the K562 and U937 cell lines and CD14+ primary monocytes. Datasets are not normalized with the combined histone distribution profiles obtained for H2B and H3 in each cell line. Hypothetical gene structures are shown at the bottom of the figure. Median P-value obtained from bootstrapping for exons and introns across all 19 histone modifications tested in this study was <1.0×10^−15^. Median P-value obtained for pair-wise t-tests between adjacent exon-intron pairs (exon_2_ → exon_10_) for the data shown in the figure was 1.13×10^−6^.
(0.94 MB TIF)

Figure S7 Histone modification and chromatin accessibility (FAIRE) patterns track exons and introns across gene bodies of non-expressed genes and at 3′ ends of expressed genes. Histograms show the mean levels of ChIP-chip enrichments for histone modifications or FAIRE values (Z-scores) spanning the first ten exons and nine introns or last five exons and four introns of consensus genes (hypothetical gene structures are shown at the bottom of each panel of the figure). Data is derived from ENCODE regions in the K562 and U937 cell lines and CD14+ primary monocytes. a. Five histone modifications across first 10 exons and 9 introns of non-expressed genes with histone normalization (n = 92, exons∶introns  = 393∶136). b. 14 histone modifications and FAIRE levels across last five exons and four introns of expressed genes with histone normalization (n = 268, exons∶introns  = 848∶226). c. Five histone modifications across first ten exons and nine introns of non-expressed genes without histone normalization (n = 92, exons∶introns  = 393∶136). d. 14 histone modifications and FAIRE levels across last five exons and four introns of expressed genes without histone normalization (n = 268, exons∶introns  = 848∶226). Median P-values obtained from bootstrapping for exons and introns across all patterns shown were <1.0×10^−15^ (panel a), <1.0×10^−15^ (panel b), <1.0×10^−15^ (panel c), and <1.0×10^−15^ (panel d). Median P-values obtained for pair-wise t-tests between adjacent exon-intron pairs were 3.15×10^−2^ (panel a), 4.40×10^−4^ (panel b), 1.35×10^−2^ (panel c), and 1.49×10^−7^ (panel d).
(1.27 MB TIF)

Figure S8 Cell type specificity of exon-intron marking by histone modifications cannot be accounted for by nucleosome distributions. Histograms show the mean levels of ChIP-chip enrichments (Z-scores) for histone modifications across exons and introns of consensus expressed (ON) (green) or non-expressed (OFF) genes (red). Data is derived from ENCODE regions in the K562 and U937 cell lines and CD14+ primary monocytes and each cell line is shown in separate panels. Histone modifications assayed are shown along the top of the figure. Levels of histones are also shown for each cell line at the right of the figure. Data for each cell line is derived as follows. K562 expressed genes (n = 76, exons/introns  = 713/290), non-expressed genes (n = 25, exons/introns  = 134/76); U937 - expressed genes (n = 88, exons/introns  = 801/327), non-expressed genes (n = 20, exons/introns  = 128/75); CD14+ monocytes - expressed genes (n = 80, exons/introns  = 681/285), non-expressed genes (n = 27, exons/introns  = 228/98). All exonic levels were determined for canonical exons only. Error bars are 95% confidence intervals.
(1.02 MB TIF)

Figure S9 Sequential ChIP-chip enhances histone modification tracking of exon-intron structures in the K562 cell line. Sequential-ChIP-chip was performed using two combinations of primary (1°, blue) and secondary (2°, red) ChIP assays which showed exon-intron tracking across gene bodies (panels a and b). Two control sequential ChIP-chip experiments were also performed (panels c and d). In all cases, data was analyzed to take into account nucleosome distribution (i.e., normalized with respect to histone H2B and H3 density). a. Consensus gene plot showing mean enrichment levels (Z-scores) across expressed (ON) (n = 111) or non-expressed (OFF) genes (n = 53) in the K562 cell line from ENCODE regions: 1° with anti-H3K36me3 and sequential 2° with anti-H3K27me1 (top panel); 1° with anti-H3K27me3 and sequential 2° with anti-H3K36me1 (bottom panel). Proportional gene length and flanking regions are shown on the x axis as percentages (%). b. Histograms show the levels of combinations of histone modifications across the first ten exons and nine introns of consensus expressed (ON) genes (n = 85, exons∶introns  = 499∶185) or non-expressed (OFF) genes (n = 26, exons∶introns  = 132∶54) from panel a. Hypothetical gene structures are shown below the panel. Median P-values obtained from bootstrapping for exons and introns were <1.0×10^−15^ (expressed genes) and <1.0×10^−15^ (non-expressed genes). Median P-values obtained for pair-wise t-tests between adjacent exon-intron pairs (exon_2_ → exon_10_) were 3.63×10^−4^ (expressed genes) and 1.24×10^−3^ (non-expressed genes). **c.** Consensus gene plot showing mean enrichment levels (Z-scores) across expressed (ON) (n = 111) genes in the K562 cell line from ENCODE regions: 1° with anti-H3K27me1 and sequential 2° with anti-H3K36me3 (top panel); 1° with anti-H3K4me3 and sequential 2° with anti-H3K9me3 (bottom panel). d. Histograms show the levels of combinations of histone modifications across the first ten exons and nine introns of consensus expressed genes (ON) (n = 85, exons∶introns  = 499∶185) from panel c. Hypothetical gene structures are shown below the panel. The first control sequential ChIP-chip (H3K27me1 → H3K36me3) was used to demonstrate that sequential ChIP-chip gave the same result irrespective of which antibody was used first (see panels a and b for reversed combination). The second control sequential ChIP-chip (H3K4me3 → H3K9me3) was used to show a decrease in exon-intron marking for two modifications which showed opposing exon-intron biases (H3K4me3  =  exon enrichment bias; H3K9me3  =  exon depletion bias). Median P-values obtained from bootstrapping for exons and introns were <1.0×10^−15^ (H3K27me1 → H3K36me3 combination) and <1.0×10^−15^ (H3K4me3 → H3K9me3 combination). Median P-values obtained for pair-wise t-tests between adjacent exon-intron pairs (exon_2_ → exon_10_) were 6.79×10^−5^ (H3K27me1 → H3K36me3 combination) and 0.16 (H3K4me3 → H3K9me3 combination). Thus, exon-intron tracking for this latter combination was no longer statistically significant.
(0.85 MB TIF)

Figure S10 Histograms show the levels of sequential-ChIP-chip enrichments for combinations of histone modifications (those described in Figure S9) spanning typical canonical/alternatively-spliced exons (CE and AE respectively) and introns (I) of expressed genes [n = 85, canonical exons:alternatively-spliced exons∶introns  = 145∶68∶221 (5′ ends) or 796∶166∶976 (gene bodies)]. Blue bars show ChIP-chip enrichments after 1° antibody and red bars show ChIP-chip enrichment after the 2° antibody. Error bars are 95% confidence intervals. In all panels, histone modification ChIP-chip enrichment levels are expressed as mean Z-scores.
(0.84 MB TIF)

Figure S11 Histone modifications differentially mark canonical and alternatively-spliced exons and introns across non-expressed genes. Histograms show the mean levels (Z-scores) for histone modifications and histones (ChIP-chip enrichments) or chromatin accessibility (FAIRE) spanning typical canonical/alternatively-spliced exons and introns. Data was derived from gene bodies of non-expressed genes (n = 92, canonical exons:alternatively-spliced exons:introns  = 631∶184∶826) in the K562 and U937 cell lines and CD14+ primary monocytes across the ENCODE regions. Histone distribution was based on the combined data for H2B and H3 in each cell type. Biases favoring either canonical exon or intron are summarized by the difference in Z-scores shown above each assay in grey. Positive (+) differences in Z-scores reflect exon biases, while negative (−) differences reflect intron biases. Error bars are 95% confidence intervals.
(0.76 MB TIF)

Figure S12 FAIRE accessibility assays show introns are preferentially accessible across three cell types. Histograms show the mean levels of FAIRE enrichments (Z-scores) across exons and introns of consensus expressed (ON) (green) or non-expressed (OFF) genes (red). Data is derived from ENCODE regions in the K562 and U937 cell lines and CD14+ primary monocytes and each cell line is shown separately. Datapoints for each cell line were derived as follows. K562 expressed genes (n = 76, exons/introns  = 713/290), non-expressed genes (n = 25, exons/introns  = 134/76); U937 - expressed genes (n = 88, exons/introns  = 801/327), non-expressed genes (n = 20, exons/introns  = 128/75); CD14+ monocytes - expressed genes (n = 80, exons/introns  = 681/285), non-expressed genes (n = 27, exons/introns  = 228/98). All exonic levels were determined for canonical exons only. Error bars are 95% confidence intervals.
(0.50 MB TIF)

Figure S13 RNA polymerase II (Pol II) occupancy levels are not accounted for by nucleosome distributions. Histograms show the mean levels of ChIP-chip enrichments (Z-scores) for Pol II and histones across exons and introns of consensus expressed (ON) (green) or non-expressed (OFF) genes (red). a. Data derived from ENCODE regions in the K562 cell line: expressed genes (n = 76, exons/introns  = 707/287), non expressed genes (n = 25, exons/introns  = 133/76). b. Data derived from U937 cell line: expressed genes (n = 88, exons/introns  = 797/325), non expressed genes (n = 20, exons/introns  = 123/73). All exonic levels were determined for canonical exons only. Error bars are 95% confidence intervals.
(0.52 MB TIF)

Figure S14 Genome-wide patterns of H3K27me1, H3K27me3, H3K36me1 and H3K36me3 for expressed and non-expressed genes in the K562 cell line. a. Consensus gene plots for four histone modifications across expressed (ON) genes (n = 2066). b. Consensus gene plots for four histone modifications across non-expressed (OFF) genes (n = 1973). ChIP-chip enrichment levels in both panels are expressed as mean Z-scores. Proportional gene length and flanking regions are shown on the x axis as percentages (%). Color key to modifications depicted in each panel are shown to the right of the figure.
(0.60 MB TIF)

Figure S15 Histone modifications patterns mark exon-intron structures across the whole human genome. a. Histograms show the level of H3K27me1 (bin 0%-10%, n = 662, exon∶introns  = 4378∶3697) and H3K36me3 (bin 0%–25%, n = 1657, exon∶introns  = 10911∶9194) across the first ten exons and nine introns of consensus non-expressed (OFF) genes. Exon numbering is at the bottom of panel. b. Histograms show the levels of ChIP-chip enrichments for H3K27me3 [bin 95%–100%, n = 332, canonical exons:alternatively-spliced exons∶introns  = 642∶165∶811 (5′ ends) or 2415∶402∶2817 (gene bodies)] and H3K36me1 [bin 90%–100%, n = 700, canonical exons:alternatively-spliced exons∶introns  = 1385∶400∶1803 (5′ ends) or 5750∶882∶6649 (gene bodies)] spanning typical canonical (dark green)/alternatively-spliced (light green) exons and introns (olive green) of expressed (ON) genes. Error bars are 95% confidence intervals. In both panels, ChIP-chip enrichments obtained from genome-wide analysis of the K562 cell line are expressed as mean Z-scores.
(0.51 MB TIF)

Figure S16 Histone modification patterns show relationships with either exon inclusion or with exon exclusion within gene bodies of highly expressed genes. Histograms show the levels of four histone modifications for the top ten percent of expressed genes in the K562 cell line. Genes (991) were placed into two bins: 90–95% and 95%–100% based on ranked expression level. Analysis shown was based on canonical exons  = 6967, introns  = 7714, and alternatively-spliced exons 221: 1040 (0–50% inclusion: 50–100% inclusion). Error bars are 95% confidence intervals. In all panels, ChIP-chip enrichments obtained from genome-wide analysis of the K562 cell line are expressed as mean Z-scores.
(0.93 MB TIF)

Figure S17 Changes in gene expression levels are accompanied by changes in histone modification levels across gene bodies. Levels of H3K36me3, H3K27me1, H3K36me1, and H3K27me3 for genes which show differential expression between cell types (K562, U937 and CD14+) are shown. Histone modification ChIP-chip enrichment levels (Z-scores) for canonical exons (red) and introns (green) are shown on the y axis. Gene names and their expression levels in two different cell types (level of expression - low → high or off → on - denoted by the black triangle) are shown below the x axis. H3K36me3 and H3K27me1 both show exon enrichment biases for all differentially-expressed gene pairs shown. However, both H3K36me1 and H3K27me3 show either exon or intron enrichment biases depending on the level of gene expression, which are consistent with the whole genome datasets shown in Figure 5. Error bars are 95% confidence intervals.
(1.13 MB TIF)

Figure S18 Determination of specificity of antibodies used in this study by dot blot analysis. Antibodies raised against histone modifications were each hybridized to a panel of relevant methyl-modified or acetyl modified peptides of histone H3 and histone H4 and their unmodified forms (see also Materials and Methods). Antibodies (Ab) and peptides (Pep) used are shown on the left and top of each panel respectively. Images in each panel are composites of different hybridizations denoted by the black lines dividing the sections of the panels. a. H3K9 methyl modifications. b. H3K36 methyl modifications. c. H3K27 methyl modifications. d. H3K79 methyl modifications. e. H3K9, 18, 27 acetyl modifications. f. H3K4 methyl modifications. g. H4K16 acetyl modification. In all panels, the results are shown for peptides spotted onto the immunoblot at a concentration of 25 ng/µl.
(1.44 MB TIF)

Table S1 Concordance of histone modification exon-intron marking biases between cell types. Table shows level of agreement in exon-intron marking biases for K562, U937 cell lines and CD14+ primary monocytes. Histone modifications are shown in columns along the top. Pairwise comparisons between cell lines are shown along the left-hand side. Each black box shows when the exon-intron marking bias for a histone modification (either an exon or intron bias) is in agreement between two cell types for either expressed (ON) (green boxes) or non-expressed (OFF) (red boxes) genes. White boxes represent discordance. Concordance was scored based on the data presented in Supplementary Figure S8. Overall concordance between all three cell types is shown in the bottom row of the table.
(0.02 MB DOC)

Table S2 Antibodies and peptides used in this ChIP-chip study. a. The name of the epitopes to which each antibody used in ChIP-chip is given in the first column. The supplier and the catalogue number of each antibody are given in the second and third columns respectively. Lot numbers of each antibody appears in the last column. b. The names of peptides used in dot blot analysis are shown in the first column. The supplier and the catalogue number of each peptide are given in the second and third columns respectively.
(0.08 MB DOC)

Table S3 Expressed genes in the K562 cell line across the ENCODE regions. Expressed genes were determined as described in Materials and Methods and this list reflects the intersecting top two quartiles of expression values obtained from Affymetrix GeneChip® and Sanger Institute microarray expression studies. Gene ID/name is shown in the first column. The ENCODE region, chromosome co-ordinates [(NCBI human genome build 35 (hg17)] and direction of transcript/strand are also shown in the additional columns.
(0.50 MB DOC)

Table S4 Non-expressed genes in the K562 cell line across the ENCODE regions. Non-expressed genes were determined as described in Materials and Methods and this list reflects the intersecting bottom quartile of expression values obtained from Affymetrix GeneChip® and Sanger Institute microarray expression studies. Gene ID/name is shown in the first column. The ENCODE region, chromosome co-ordinates [(NCBI human genome build 35 (hg17)] and direction of transcript/strand are also shown in the additional columns.
(0.24 MB DOC)

Table S5 Expressed genes in the U937 cell line across the ENCODE regions. Expressed genes were determined as described in Materials and Methods and this list reflects the intersecting top two quartiles of expression values obtained from Affymetrix GeneChip® and Sanger Institute microarray expression studies. Gene ID/name is shown in the first column. The ENCODE region, chromosome co-ordinates [(NCBI human genome build 35 (hg17)] and direction of transcript/strand are also shown in the additional columns.
(0.57 MB DOC)

Table S6 Non-expressed genes in the U937 cell line across the ENCODE regions. Non-expressed genes were determined as described in Materials and Methods and this list reflects the intersecting bottom quartile of expression values obtained from Affymetrix GeneChip® and Sanger Institute microarray expression studies. Gene ID/name is shown in the first column. The ENCODE region, chromosome co-ordinates [(NCBI human genome build 35 (hg17)] and direction of transcript/strand are also shown in the additional columns.
(0.29 MB DOC)

Table S7 Expressed genes in CD14+ monocytes across the ENCODE regions. Expressed genes were determined as described in Materials and Methods and this list reflects the top two quartiles of expression values obtained from Illumina BeadChip® expression studies. Gene ID/name is shown in the first column. The ENCODE region, chromosome co-ordinates [(NCBI human genome build 35 (hg17)] and direction of transcript/strand are also shown in the additional columns.
(0.51 MB DOC)

Table S8 Non-expressed genes in CD14+ monocytes across the ENCODE regions. Non-expressed genes were determined as described in Materials and Methods and this list reflects the bottom quartile of expression values obtained from Illumina BeadChip® expression studies. Gene ID/name is shown in the first column. The ENCODE region, chromosome co-ordinates [(NCBI human genome build 35 (hg17)] and direction of transcript/strand are also shown in the additional columns.
(0.27 MB DOC)

Table S9 Expression levels of 9922 genes across the human genome in the K562 cell line. These genes gave consistent MAS5 values (all A or all P) in four bioreplicate Affymetrix GeneChIP® expression experiments and all had been assigned ENSEMBL identifiers. List shows their ENSEMBL IDs, MAS5 call and ranking based on RMA values.
(0.36 MB TXT)
