# Supplementary material for: Complex Exon-Intron Marking by Histone Modifications Is Not Determined Solely by Nucleosome Distribution
Source: PLoS One. 2010 Aug 23;5(8):e12339. doi: 10.1371/journal.pone.0012339 (PMC2925886; doi:10.1371/journal.pone.0012339)
Supplement: Protocol S1 — Protocol for Sequential-ChIP (Seq-ChIP) used in this study. All reagents (including suppliers and catalogue numbers) used for Seq-Chip assays are shown at the top of the protocol. For details of hybridization of these samples to Sanger Institute tiling microarrays, refer to our previous publications1,2 1. Koch, C.M. et al. The landscape of histone modifications across 1% of the human genome in five human cell lines. Genome Res 17, 691-707 (2007). 2. Bruce, A.W., Lopez-Contreras, A., Flicek, P., Down, T.A., Dhami, P., Dillon, S.C., Koch, C.M., Langford, C.F., Dunham, I., Andrews, R.M. and Vetrie, D. Functional diversity for REST (NRSF) is defined by in vivo binding affinity hierachies at the DNA sequence level. Genome Res 19, 994-1005 (2009). (2.28 MB DOC) [file pone.0012339.s001.doc]

**PROTOCOL S.1: Sequential Chromatin Immunoprecipitation (Seq-ChIP) of DNA Sequences that Interact with Two Methylated Histone H3 species.**

***Reagents***

- cell lysis buffer (10 mM Tris-HCl pH 8.0, 10 mM NaCl, 0.2% NP40, 10mM sodium butyrate, 50 ųg/ml PMSF, 1 ųg/ml leupeptin)
- formaldehyde solution (0.37% final concentration, BDH AnalaR, 101135 5B)
- 2 M glycine (BDH AnalaR, 10119 4M)
- 50 ml Falcon tubes
- 5 ml Falcon tubes
- phosphate-buffered saline (PBS)
- nuclear lysis buffer (50 mM Tris-HCl pH 8.1, 10 mM EDTA, 1% SDS, 10mM sodium butyrate, 50 ųg/ml PMSF, 1 ųg/ml leupeptin)
- IP dilution buffer (IPDB) (20mM Tris-HCl pH 8.1, 150 mM NaCl, 2mM EDTA, 1% Triton X-100, 0.01% SDS, 10mM sodium butyrate, 50 ųg/ml PMSF, 1 ųg/ml leupeptin)
- Sanyo/MES Soniprep sonicator
- 2 ml microcentrifuge tubes
- 15 ml Falcon tubes
- normal rabbit IgG (Upstate Biotechnology, 12-370)
- protein G-agarose (Roche, I719 416)
- 1^st^ anti-histone methylation IgG
- 2^nd^ anti-histone methylation IgG
- IP wash buffer 1 (IPWB1) (20 mM Tris-HCl pH 8.1, 50 mM NaCl, 2 mM EDTA, 1% Triton X-100, 0.1% SDS)
- IP wash buffer 2 (IPWB2) (10 mM Tris-HCL pH 8.1, 250 mM LiCl, 1 mM EDTA, 1% NP-40, 1% deoxycholic acid)
- TE pH8.0
- IP elution buffer (IPEB) (100 mM NaHC0_3_, 1% SDS)
- 0.1M DTT
- RNase A (10mg/ml stock) (ICN Biochemicals, 101076)
- 5M NaCl
- proteinase K (10mg/ml stock) (Gibco-BRL 25530-031)
- tRNA (5mg/ml stock) (Gibco-BRL 15401-029)
- phenol/chloroform (Rathburn, RP3024 and RH1009, purchased separately)
- chloroform (Rathburn RH1009)
- glycogen (Roche 901 393)
- 3M sodium acetate pH5.2
- 100% ethanol
- 70% ethanol
- 1% agarose 1XTBE gel

***Method***

**Day 1**

**A. Preparation of Chromatin:**

1. Grow and harvest 1 x 10^8^ cells. Collect the cells by centrifuging at 1200 rpm for 5-8 minutes.
2. Resuspend the cells in 50 ml serum-free media in glass flask. Add 1.36 ml of formaldehyde solution (37%; final concentration 1.0%) to cross-link DNA-protein interactions. Incubate at room temperature with gentle agitation for 10 minutes.
3. Add 3.43 ml of 2M glycine (final concentration of 0.125M) and incubate for 5 minutes at room temperature with gentle shaking to stop the cross-linking reaction.
4. Transfer the cells to a 50 ml Falcon tube on ice and centrifuge the cells at 1200 rpm for 6 minutes at 4ºC.
5. Resuspend the pellet with 1.5 ml ice-cold PBS and keep on ice. Centrifuge the cells at 2000 rpm for 5 minutes at 4ºC.
6. Gently resuspend the cell pellet in 1.5X pellet volume of cell lysis buffer (CLB). Resuspend cells by pipetting up and down and incubate for 10 minutes on ice. Centrifuge at 2500 rpm for 5 minutes at 4ºC to collect nuclei.
7. Remove the supernatant and resuspend nuclei in 1.2 ml of nuclei lysis buffer (NLB) and incubate on ice for 10 minutes. Add 0.72 ml of IP dilution buffer (IPDB) and transfer to 5 ml Falcon tube.
8. Sonicate the sample using a microtip. Keep the tip of the probe just below surface and keep the sample on ice at all times. Using the Sanyo/MES Soniprep sonicator, the settings are the following (*these settings may vary from instrument to instrument):
   1. Amplitude (microns): 14
   2. No. of bursts : 8
   3. Length of bursts: 30 seconds
   4. Allow the samples to cool for 1 min in an ice/water bath between each pulse.
9. Using these conditions, the DNA is sheared to approximately 300-1000 bp fragments. Transfer the sheared chromatin to 2 ml microcentrifuge tubes and centrifuge at 13000 rpm for 5 minutes at 4ºC.
10. Transfer the supernatant to a 15 ml Falcon and add 4.1 ml of IPDB to each tube to bring the ratio of NLB:IPDB to 1:4.
11. Pre-clear chromatin by adding 100 µl of normal rabbit IgG and incubate for 1 hour at 4ºC on a rotating wheel. Add 200 µl of the homogeneous protein G-agarose suspension (100 µl of the bed volume) and incubate for 3 hours (to overnight) at 4ºC on a rotating wheel.

**B. First Round ChIP:**

1. Centrifuge the beads at 3000 rpm for 2 minutes at 4ºC. Use the supernatant to set up the following conditions in 2 ml tubes: N.B. The matrix below shows the conditions for the first and second IP reactions using 1x10^7^ cells equivalent of cross-linked chromatin. All experimental IPs are done in duplicate. All controls are shown.

| Sample # | First IP | Second IP | Vol. Chromatin (mL) | Vol. IPDB^mod^ (mL) |
| --- | --- | --- | --- | --- |
| 1 | 1^st^ anti-me IgG (8μg) | rabbit IgG (8μg) | 0.6 | 0.75 |
| 2 | 1^st^ anti-me IgG (8μg) | 2^nd^ anti-me IgG (8μg) | 0.6 | 0.75 |
| 3 | 1^st^ anti-me IgG (8μg) | 2^nd^ anti-me IgG (8μg) | 0.6 | 0.75 |
| 4 | 1^st^ anti-me IgG (8μg) | **ELUTE & STOP** | 0.6 | 0.75 |
| 5 | 2^nd^ anti-me IgG (8μg) | rabbit IgG (8μg) | 0.6 | 0.75 |
| 6 | 2^nd^ anti-me IgG (8μg) | 1^st^ anti-me IgG (8μg) | 0.6 | 0.75 |
| 7 | 2^nd^ anti-me IgG (8μg) | 1^st^ anti-me IgG (8μg) | 0.6 | 0.75 |
| 8 | 2^nd^ anti-me IgG (8μg) | **ELUTE & STOP** | 0.6 | 0.75 |
| 9 | Rabbit IgG (8μg) | **ELUTE & STOP** | 0.6 | 0.75 |
| 10 | Rabbit IgG (8μg) | Rabbit IgG (8μg) | 0.6 | 0.75 |

*Use 270µl of the chromatin to set up an INPUT control and store at -20ºC. The final concentration of the anti-serum in the immuno-precipitation conditions is usually between 0.8 to 1.6%. Incubate overnight at 4ºC with rotation.

** IPDB^mod^ is made by mixing 1 part NLB to 4 parts IPDB.

**Day 2**

1. Centrifuge the samples at 13000 rpm for 5 minutes at 4ºC. Transfer the samples to new 1.5 ml microcentrifuge tubes and add 50 µl of the homogenous protein G-agarose suspension (25µl of the bed volume). Incubate for at least 3 hours at 4ºC with rotation.
2. Centrifuge the protein G-agarose beads at 13000 rpm for 20 seconds at 4ºC. Remove the supernatant and wash the pellet twice with 750 µl of IP wash buffer 1 (IPWB1). For each wash, vortex briefly and centrifuge at 7500 rpm for 2 minutes at 4ºC. Leave the tubes undisturbed for a minute before removing the supernatant.
3. Similarly, wash the bead pellet, once with 750 µl of IP wash buffer 2 (IPWB2) and twice with 750 µl of TE pH8.0.
4. Elute the immune complexes (DNA-protein-antibody) from the beads by adding 200µl of IP elution buffer (IPEB). Vortex and place on multi-tube shaker for 10 minutes. Centrifuge at 7500 rpm for 2 minutes and transfer supernatant to a new tube. The supernatant contains the immunoprecipitated material.

**C. Second Round ChIP:**

1. For sequential ChIP samples, add 50µl of 0.1M DTT (final conc. 20mM) to the material derived from the first IP, vortex, pulse spin and incubate at 37^o^C for 30 minutes (mix regularly).
2. Transfer to a 14mL Falcon tube and add 10mL (approximately 50-fold dilution) of IPDB (not IPDB^mod^) and rotate on the blood wheel for 10 minutes.
3. Add the second antibody for the second IP condition (refer to the matrix) and place on the blood wheel at 4^o^C for at least 24 hours.

**Day 3**

1. Add 100µl of protein G bead slurry and incubate for a further 24 hours.
2. Spin the tubes at 2000 rpm to pellet the protein G beads and resuspend in 750µl of IPWB1. Transfer to fresh 2mL tubes and wash as for first IP.
3. Elute the samples from the beads by adding 225µl of IPEB. Vortex briefly and centrifuge at 7500 rpm for 2 minutes. Repeat this step and combine the elutions in the same tube.

**D. Extraction of ChIP DNAs from 1^st^ and 2^nd^ Round ChIP:**

1. Add 0.2µl of RNase A (10mg/ml stock) and 27 µl of 5M NaCl (final concentration of 0.3M) to each sample. Also add 0.1 µl of RNase A (10mg/ml stock) and 16.2 µl of 5M NaCl to the input sample. Incubate the samples at 65ºC for 6 hours.
2. Add 9µl of proteinase K (10mg/ml stock) and incubate overnight at 45ºC

**Day 4**

1. Add 2 µl tRNA (5mg/ml stock) immediately before adding 500 µl of phenol/chloroform. Vortex well, centrifuge at 13200 rpm for 5 minutes at room temperature. Transfer the aqueous layer to new 2 ml microcentrifuge tubes and repeat this step once with 500 µl of chloroform.
2. Add 5 ųg of glycogen, 1µl of tRNA (5mg/ml stock) and 50µl of 3M sodium acetate pH5.2 to each sample. Vortex well and add 1.25 ml of 100% ethanol. Precipitate at -70ºC for 30 minutes.
3. Centrifuge at 13200 rpm for 20 minutes at 4ºC. Wash the pellet with 500 µl of ice-cold 70% ethanol.
4. Remove the supernatant and air dry the pellets for 10 minutes. Resuspend the pellets in 100 µl of water for the input and 50µl of water for the other samples.
5. Electrophorese 5 µl of each sample on a 1% agarose 1XTBE gel to check DNA size. Quantitate DNA yield using a fluorometer/bioanalyzer/nanodrop. Store samples at -20ºC.

Supplementary Table S.1

| **ANTIBODY EPITOPE** | **SUPPLIER** | **CATALOGUE NUMBER** |
| --- | --- | --- |
| Rabbit IgG | Millipore (Upstate) | 12-370 |
| Mouse IgG | Millipore (Upstate) | 12-371 |
| RNA polymerase II | Abcam | ab5408 |
| histone H3 acetyl K9 | Millipore (Upstate) | 07-352 |
| histone H3 acetyl K18 | Millipore (Upstate) | 07-354 |
| histone H3 acetyl K27 | Millipore (Upstate) | 07-360 |
| histone H4 acetyl K16 | Abcam | ab1762 |
| histone H3 monomethyl K4 | Abcam | ab8895 |
| histone H3 dimethyl K4 | Abcam | ab7766 |
| histone H3 trimethyl K4 | Abcam | ab8580 |
| histone H3 monomethyl K9 | Abcam | ab9045 |
| histone H3 dimethyl K9 | Millipore (Upstate) | 07-212 |
| histone H3 trimethyl K9 | Millipore (Upstate) | 07-523 |
| histone H3 monomethyl K27 | Millipore (Upstate) | 07-448 |
| histone H3 dimethyl K27 | Abcam | ab1781 |
| histone H3 trimethyl K27 | Millipore (Upstate) | 07-449 |
| histone H3 monomethyl K36 | Abcam | ab9048 |
| histone H3 dimethyl K36 | Millipore (Upstate) | 07-274 |
| histone H3 trimethyl K36 | Abcam | ab9050 |
| histone H3 monomethyl K79 | Abcam | ab2886 |
| histone H3 dimethyl K79 | Abcam | ab3594 |
| histone H3 trimethyl K79 | Abcam | ab2621 |
| histone H3 | Abcam | ab1791 |
| histone H2B | Abcam | ab1790 |

Supplementary Table S.2

| **Gene ID** | **Region** | **Chr** | **Start** | **End** | **Strand** |
| --- | --- | --- | --- | --- | --- |
| AC000059.1 | ENm013 | 7 | 89905872 | 89906534 | 1 |
| AC000110.1 | ENm014 | 7 | 126529626 | 126530209 | 1 |
| AC000123.4 | ENm014 | 7 | 126584134 | 126585529 | 1 |
| AC000362.1 | ENm014 | 7 | 126214973 | 126215784 | 1 |
| AC002465.2 | ENm001 | 7 | 116534867 | 116556242 | 1 |
| AC004079.2 | ENm010 | 7 | 26861029 | 26861488 | 1 |
| AC004079.4 | ENm010 | 7 | 26836598 | 26837769 | 1 |
| AC004079.7 | ENm010 | 7 | 26908984 | 26913118 | 1 |
| AC004080.15 | ENm010 | 7 | 26981759 | 26984775 | 1 |
| AC004775.1 | ENm002 | 5 | 132111037 | 132117756 | 1 |
| AC006153.3 | ENm013 | 7 | 89614337 | 89659127 | 1 |
| AC006326.2 | ENm001 | 7 | 116497868 | 116498400 | 1 |
| AC006326.4 | ENm001 | 7 | 116497531 | 116497848 | 1 |
| AC007568.1 | ENm001 | 7 | 117116865 | 117117681 | 1 |
| AC008746.5 | ENm007 | 19 | 59649821 | 59650038 | 1 |
| AC009404.2 | ENr121 | 2 | 118307744 | 118315465 | 1 |
| AC009404.7 | ENr121 | 2 | 118304691 | 118313637 | 1 |
| AC009502.2 | ENr331 | 2 | 220388027 | 220388442 | 1 |
| AC009802.2 | ENr122 | 18 | 59767516 | 59799989 | 1 |
| AC009955.4 | ENr331 | 2 | 220233891 | 220240823 | 1 |
| AC010492.2 | ENm007 | 19 | 59424724 | 59425901 | 1 |
| AC010492.5 | ENm007 | 19 | 59463672 | 59464712 | 1 |
| AC021607.1 | ENr122 | 18 | 59890372 | 59890867 | 1 |
| AC023356.3 | ENr233 | 15 | 41955340 | 41955735 | -1 |
| AC034228.4 | ENm002 | 5 | 131308001 | 131331787 | 1 |
| AC051649.10 | ENm011 | 11 | 1868985 | 1870069 | 1 |
| AC053503.7 | ENr331 | 2 | 220125074 | 220183860 | 1 |
| AC053503.8 | ENr331 | 2 | 220186616 | 220187322 | 1 |
| AC063976.4 | ENm002 | 5 | 131548469 | 131556401 | 1 |
| AC063976.7 | ENm002 | 5 | 131563049 | 131567080 | 1 |
| AC068580.6 | ENm011 | 11 | 1738155 | 1740293 | 1 |
| AC073472.1 | ENm010 | 7 | 26734970 | 26735772 | 1 |
| AC080091.1 | ENr112 | 2 | 51648932 | 51650273 | 1 |
| AC084290.2 | ENr123 | 12 | 38966359 | 38966732 | 1 |
| AC098789.1 | ENm007 | 19 | 59463671 | 59464709 | 1 |
| AC104389.19 | ENm009 | 11 | 5219927 | 5221344 | -1 |
| AC104389.32 | ENm009 | 11 | 5340422 | 5340656 | 1 |
| AC116366.4 | ENm002 | 5 | 131774228 | 131839636 | 1 |
| AC139143.1 | ENm011 | 11 | 1770360 | 1770677 | 1 |
| ACCN4 | ENr331 | 2 | 220204398 | 220229000 | 1 |
| ADTB1L1 | ENm004 | 22 | 30842519 | 30854011 | 1 |
| ADTB1L2 | ENm004 | 22 | 30854570 | 30854675 | 1 |
| AF064859.2 | ENr133 | 21 | 39421365 | 39421837 | 1 |
| AF121781.17 | ENr133 | 21 | 39704666 | 39722325 | 1 |
| AF129408.14 | ENr133 | 21 | 39510421 | 39511303 | 1 |
| AF277315.13 | ENm006 | X | 153446525 | 153447304 | 1 |
| AL162151.1 | ENr322 | 14 | 98508970 | 98509392 | -1 |
| AL162151.3 | ENr322 | 14 | 98509405 | 98509562 | 1 |
| AP000271.1 | ENm005 | 21 | 32790994 | 32791493 | 1 |
| AP000279.69 | ENm005 | 21 | 33022298 | 33037305 | 1 |
| AP000297.1 | ENm005 | 21 | 33628748 | 33629273 | 1 |
| AP000569.8 | ENm005 | 21 | 34225335 | 34271863 | 1 |
| AP000936.1 | ENm003 | 11 | 116411724 | 116411978 | -1 |
| AP001187.10 | ENr332 | 11 | 64418595 | 64438549 | -1 |
| AP001187.11 | ENr332 | 11 | 64405584 | 64405994 | 1 |
| AP003476.1 | ENr321 | 8 | 119184251 | 119185263 | 1 |
| AP006216.10 | ENm003 | 11 | 116149316 | 116150132 | 1 |
| ARD1 | ENm006 | X | 152715543 | 152721524 | -1 |
| ARF5 | ENm014 | 7 | 126822351 | 126825711 | 1 |
| ATF4P | ENm006 | X | 153372894 | 153373966 | 1 |
| ATP11A | ENr132 | 13 | 112392645 | 112589484 | 1 |
| ATP6AP1 | ENm006 | X | 153177832 | 153183640 | 1 |
| AVPR2 | ENm006 | X | 152688833 | 152693468 | 1 |
| AXIN1 | ENm008 | 16 | 277442 | 342675 | -1 |
| BIRC4 | ENr324 | X | 122719110 | 122773365 | 1 |
| BRCC3 | ENm006 | X | 153863400 | 153915054 | 1 |
| C16orf33 | ENm008 | 16 | 43011 | 47670 | 1 |
| C16orf35 | ENm008 | 16 | 74274 | 128860 | -1 |
| C20orf52 | ENr333 | 20 | 33750609 | 33752321 | 1 |
| C21orf119 | ENm005 | 21 | 32687311 | 32688141 | 1 |
| C21orf59 | ENm005 | 21 | 32886261 | 32907048 | -1 |
| C5orf35 | ENr221 | 5 | 56240845 | 56248933 | 1 |
| C6orf49 | ENr334 | 6 | 41856066 | 41865858 | 1 |
| CAPZA2 | ENm001 | 7 | 116045076 | 116153267 | 1 |
| CAV1 | ENm001 | 7 | 115758791 | 115795182 | 1 |
| CAV2 | ENm001 | 7 | 115522079 | 115742547 | 1 |
| CEP250 | ENr333 | 20 | 33506400 | 33563219 | 1 |
| CLDN12 | ENm013 | 7 | 89657687 | 89787368 | 1 |
| CMPK | SCL | 1 | 47511502 | 47556532 | 1 |
| CNOT3 | ENm007 | 19 | 59333257 | 59351259 | 1 |
| CPNE1 | ENr333 | 20 | 33677368 | 33725881 | -1 |
| CTA-342B11.1 | ENm004 | 22 | 30683899 | 30690942 | 1 |
| CTA-415G2.2 | ENm004 | 22 | 31735520 | 31736001 | 1 |
| CTAG1B | ENm006 | X | 153409570 | 153411238 | -1 |
| CTAG2 | ENm006 | X | 153443951 | 153445558 | -1 |
| CTSD | ENm011 | 11 | 1725474 | 1741799 | -1 |
| CXorf12 | ENm006 | X | 152758626 | 152769494 | 1 |
| DDX18 | ENr121 | 2 | 118288457 | 118306186 | 1 |
| DECR2 | ENm008 | 16 | 391828 | 402489 | 1 |
| DEPDC5 | ENm004 | 22 | 30474499 | 30627556 | 1 |
| DES | ENr331 | 2 | 220108605 | 220116967 | 1 |
| DKC1 | ENm006 | X | 153554740 | 153569669 | 1 |
| DOLPP1 | ENr232 | 9 | 128922934 | 128932272 | 1 |
| DONSON | ENm005 | 21 | 33869654 | 34206506 | -1 |
| DRG1 | ENm004 | 22 | 30131553 | 30154993 | 1 |
| DSCR2 | ENr133 | 21 | 39468566 | 39477648 | -1 |
| EEF1A1 | ENr223 | 6 | 74282195 | 74288345 | -1 |
| EHD1 | ENr332 | 11 | 64375691 | 64412345 | -1 |
| EIF4ENIF1 | ENm004 | 22 | 30159904 | 30216649 | -1 |
| EMD | ENm006 | X | 153128405 | 153130731 | 1 |
| ERGIC3 | ENr333 | 20 | 33593185 | 33608820 | 1 |
| EVX1 | ENm010 | 7 | 27055525 | 27060692 | 1 |
| F10 | ENr132 | 13 | 112825130 | 112832622 | 1 |
| FAM3A | ENm006 | X | 153298195 | 153308271 | -1 |
| FAM50A | ENm006 | X | 153236209 | 153242707 | 1 |
| FAM73B | ENr232 | 9 | 128878455 | 128913916 | 1 |
| FBXO7 | ENm004 | 22 | 31195218 | 31219371 | 1 |
| FER1L4 | ENr333 | 20 | 33609922 | 33658899 | -1 |
| FLNA | ENm006 | X | 153097742 | 153123842 | -1 |
| FOXP4 | ENr334 | 6 | 41622143 | 41678101 | 1 |
| FRS3 | ENr334 | 6 | 41845893 | 41856183 | -1 |
| FUNDC2 | ENm006 | X | 153817960 | 153852283 | 1 |
| FZD1 | ENm013 | 7 | 90538331 | 90542764 | 1 |
| GART | ENm005 | 21 | 33798109 | 33837668 | -1 |
| GDF5 | ENr333 | 20 | 33484560 | 33505983 | -1 |
| GMPPA | ENr331 | 2 | 220189095 | 220197216 | 1 |
| GTPBP10 | ENm013 | 7 | 89620631 | 89665421 | 1 |
| HBA2 | ENm008 | 16 | 162847 | 163710 | 1 |
| HBB | ENm009 | 11 | 5203271 | 5207202 | -1 |
| HBE1 | ENm009 | 11 | 5246159 | 5483424 | -1 |
| HBG1 | ENm009 | 11 | 5225890 | 5227699 | -1 |
| HBG2 | ENm009 | 11 | 5230997 | 5623596 | -1 |
| HBQ1 | ENm008 | 16 | 170453 | 171181 | 1 |
| HBZ | ENm008 | 16 | 142687 | 144503 | 1 |
| HCFC1 | ENm006 | X | 152733852 | 152758106 | -1 |
| HMGN1 | ENr133 | 21 | 39636112 | 39643444 | -1 |
| HOXA10 | ENm010 | 7 | 26983451 | 26993121 | -1 |
| HYPK | ENr233 | 15 | 41875633 | 41882534 | 1 |
| IFNAR1 | ENm005 | 21 | 33618653 | 33654039 | 1 |
| IFNAR2 | ENm005 | 21 | 33524077 | 33559840 | 1 |
| IFNGR2 | ENm005 | 21 | 33679170 | 33773526 | 1 |
| IKBKG | ENm006 | X | 153333119 | 153359509 | 1 |
| IL10RB | ENm005 | 21 | 33542891 | 33591410 | 1 |
| IL3 | ENm002 | 5 | 131424122 | 131426797 | 1 |
| IL4 | ENm002 | 5 | 132037578 | 132046268 | 1 |
| INHA | ENr331 | 2 | 220259390 | 220265941 | 1 |
| IRF1 | ENm002 | 5 | 131845201 | 131854390 | -1 |
| ITFG3 | ENm008 | 16 | 224547 | 258972 | 1 |
| ITGB4BP | ENr333 | 20 | 33330129 | 33336203 | -1 |
| KCNQ5 | ENr223 | 6 | 73808381 | 73965296 | 1 |
| LACE1 | ENr323 | 6 | 108722792 | 108829952 | 1 |
| LAGE3 | ENm006 | X | 153269813 | 153271301 | -1 |
| LAIR1 | ENm007 | 19 | 59557075 | 59573978 | -1 |
| LENG4 | ENm007 | 19 | 59368922 | 59385479 | -1 |
| LENG8 | ENm007 | 19 | 59651878 | 59665030 | 1 |
| LL22NC01-116C6.1 | ENm004 | 22 | 31503718 | 31505523 | 1 |
| LSP1 | ENm011 | 11 | 1830777 | 1870074 | 1 |
| LUC7L | ENm008 | 16 | 178970 | 219464 | -1 |
| MAP1A | ENr233 | 15 | 41590449 | 41611111 | 1 |
| MAP3K1 | ENr221 | 5 | 56147159 | 56227737 | 1 |
| MDFIC | ENm012 | 7 | 114156161 | 114253208 | 1 |
| MEN1 | ENr332 | 11 | 64327565 | 64335343 | -1 |
| MFAP1 | ENr233 | 15 | 41883983 | 41904293 | -1 |
| MMP24 | ENr333 | 20 | 33305670 | 33328216 | 1 |
| MPG | ENm008 | 16 | 67007 | 75853 | 1 |
| MPP1 | ENm006 | X | 153570664 | 153612987 | -1 |
| MRPL23 | ENm011 | 11 | 1925085 | 1962329 | 1 |
| MRPL28 | ENm008 | 16 | 356929 | 360570 | -1 |
| MTCP1 | ENm006 | X | 153853602 | 153939917 | -1 |
| MYADM | ENm007 | 19 | 59061290 | 59071504 | 1 |
| NDUFA3 | ENm007 | 19 | 59297849 | 59306711 | 1 |
| NFS1 | ENr333 | 20 | 33720025 | 33750696 | -1 |
| NME4 | ENm008 | 16 | 386727 | 402489 | 1 |
| NUP188 | ENr232 | 9 | 128783312 | 128848930 | 1 |
| OR51B6 | ENm009 | 11 | 5329315 | 5330253 | 1 |
| OR51J1 | ENm009 | 11 | 5380404 | 5381354 | 1 |
| OR52D1 | ENm009 | 11 | 5466514 | 5467470 | 1 |
| OR7E23P | ENm005 | 21 | 32915231 | 32916278 | 1 |
| PDIA2 | ENm008 | 16 | 273154 | 277217 | 1 |
| PDIA3 | ENr233 | 15 | 41825883 | 41852770 | 1 |
| PDLIM4 | ENm002 | 5 | 131621264 | 131637047 | 1 |
| PFTK1 | ENm013 | 7 | 89740390 | 90484557 | 1 |
| PIK4CB | ENr231 | 1 | 148077487 | 148113265 | -1 |
| PIP5K1A | ENr231 | 1 | 147983499 | 148035086 | 1 |
| PISD | ENm004 | 22 | 30339032 | 30382973 | -1 |
| PLXNA3 | ENm006 | X | 153250326 | 153265694 | 1 |
| POGZ | ENr231 | 1 | 148188274 | 148245015 | -1 |
| POLR3K | ENm008 | 16 | 36408 | 43629 | -1 |
| PPP2R4 | ENr232 | 9 | 128952784 | 128990780 | 1 |
| PRPF31 | ENm007 | 19 | 59310650 | 59326956 | 1 |
| PSMB4 | ENr231 | 1 | 148185084 | 148187494 | 1 |
| PSMD4 | ENr231 | 1 | 148040253 | 148053029 | 1 |
| RAB11FIP3 | ENm008 | 16 | 415621 | 495629 | 1 |
| RAD50 | ENm002 | 5 | 131919611 | 132007652 | 1 |
| RASGRP2 | ENr332 | 11 | 64250960 | 64269505 | -1 |
| RBM12 | ENr333 | 20 | 33700262 | 33716253 | -1 |
| RENBP | ENm006 | X | 152721564 | 152730991 | -1 |
| RFX5 | ENr231 | 1 | 148126190 | 148132907 | -1 |
| RP11-115M6.4 | ENm006 | X | 153608306 | 153609501 | -1 |
| RP1-111B22.2 | ENr323 | 6 | 108432255 | 108433466 | -1 |
| RP11-126K1.8 | ENr231 | 1 | 148126198 | 148127516 | 1 |
| RP11-143H17.1 | ENm006 | X | 153929091 | 153948483 | 1 |
| RP11-223E19.1 | ENr111 | 13 | 29900129 | 29900593 | 1 |
| RP11-247A12.6 | ENr232 | 9 | 128936628 | 128952564 | -1 |
| RP11-247I13.3 | ENm004 | 22 | 30280024 | 30280480 | 1 |
| RP1-127L4.7 | ENm004 | 22 | 30861809 | 30863814 | 1 |
| RP1-128O3.5 | ENr323 | 6 | 108680112 | 108680298 | 1 |
| RP1-128O3.6 | ENr323 | 6 | 108745977 | 108746302 | 1 |
| RP11-298J23.7 | ENr334 | 6 | 41863379 | 41865615 | 1 |
| RP11-374F3.2 | ENr111 | 13 | 29768285 | 29768873 | 1 |
| RP11-380M3.3 | ENr223 | 6 | 73821542 | 73822302 | 1 |
| RP11-398K22.10 | ENr223 | 6 | 74089282 | 74089895 | 1 |
| RP11-398K22.12 | ENr223 | 6 | 74029660 | 74067846 | 1 |
| RP11-398K22.13 | ENr223 | 6 | 74056848 | 74057707 | 1 |
| RP11-398K22.6 | ENr223 | 6 | 74093969 | 74095462 | 1 |
| RP11-398K22.9 | ENr223 | 6 | 74058407 | 74059722 | 1 |
| RP1-149A16.16 | ENm004 | 22 | 31107210 | 31107410 | 1 |
| RP1-149A16.17 | ENm004 | 22 | 31104328 | 31106173 | 1 |
| RP11-545E17.13 | ENr232 | 9 | 129178358 | 129180129 | 1 |
| RP11-69I8.3 | ENr222 | 6 | 132313780 | 132440227 | 1 |
| RP11-74C1.2 | ENr231 | 1 | 148342996 | 148343490 | 1 |
| RP1-18D14.4 | SCL | 1 | 47356943 | 47357850 | 1 |
| RP1-18D14.7 | SCL | 1 | 47403490 | 47408443 | 1 |
| RP11-98F14.4 | ENr132 | 13 | 112832732 | 112834370 | 1 |
| RP1-248E1.2 | ENr222 | 6 | 132634815 | 132636165 | 1 |
| RP1-90G24.10 | ENm004 | 22 | 30925657 | 30990208 | 1 |
| RP1-90G24.5 | ENm004 | 22 | 30989923 | 30993652 | 1 |
| RP1-90G24.6 | ENm004 | 22 | 30993962 | 30997728 | 1 |
| RP3-429G5.3 | ENr323 | 6 | 108551413 | 108587290 | 1 |
| RP3-477O4.13 | ENr333 | 20 | 33484242 | 33486663 | 1 |
| RP4-696P19.2 | ENr334 | 6 | 41742623 | 41743366 | 1 |
| RP5-931E15.2 | ENr324 | X | 122592560 | 122593680 | 1 |
| RP5-931E15.3 | ENr324 | X | 122624687 | 122625072 | 1 |
| RP5-931E15.4 | ENr324 | X | 122644395 | 122644837 | 1 |
| RPL10 | ENm006 | X | 153147247 | 153151528 | 1 |
| RPL37P1 | ENr333 | 20 | 33639661 | 33639944 | 1 |
| RPS17P4 | ENm004 | 22 | 30760032 | 30760438 | 1 |
| SELENBP1 | ENr231 | 1 | 148149852 | 148158283 | -1 |
| SERF2 | ENr233 | 15 | 41856578 | 41882080 | 1 |
| SERPINB13 | ENr122 | 18 | 59412570 | 59422854 | 1 |
| SERPINB7 | ENr122 | 18 | 59571150 | 59623585 | 1 |
| SERPINB8 | ENr122 | 18 | 59788140 | 59823259 | 1 |
| SF1 | ENr332 | 11 | 64288655 | 64302835 | -1 |
| SFI1 | ENm004 | 22 | 30209229 | 30339092 | 1 |
| SIL | SCL | 1 | 47427870 | 47491840 | -1 |
| SLC10A3 | ENm006 | X | 153279350 | 153282707 | -1 |
| SLC22A4 | ENm002 | 5 | 131658036 | 131707799 | 1 |
| SLC22A5 | ENm002 | 5 | 131733344 | 131759206 | 1 |
| SLC4A3 | ENr331 | 2 | 220317793 | 220332208 | 1 |
| SNX27 | ENr231 | 1 | 148397615 | 148468995 | 1 |
| SNX3 | ENr323 | 6 | 108639120 | 108689158 | -1 |
| SON | ENm005 | 21 | 33836795 | 33871658 | 1 |
| SPAG4 | ENr333 | 20 | 33667229 | 33672386 | 1 |
| ST7 | ENm001 | 7 | 116187333 | 116464109 | 1 |
| ST7OT4 | ENm001 | 7 | 116187905 | 116202580 | 1 |
| STAG2 | ENr324 | X | 122819598 | 122962042 | 1 |
| STEAP1 | ENm013 | 7 | 89428341 | 89438795 | 1 |
| STK11IP | ENr331 | 2 | 220288088 | 220306679 | 1 |
| TAZ | ENm006 | X | 153160702 | 153170913 | 1 |
| TES | ENm001 | 7 | 115444499 | 115492789 | 1 |
| TIMP3 | ENm004 | 22 | 31522242 | 31583585 | 1 |
| TMEM15 | ENr232 | 9 | 128787364 | 128789453 | -1 |
| TMEM50B | ENm005 | 21 | 33726663 | 33775370 | -1 |
| TMEM8 | ENm008 | 16 | 360775 | 377115 | -1 |
| TNNI2 | ENm011 | 11 | 1816796 | 1819485 | 1 |
| TRIM22 | ENm009 | 11 | 5667496 | 5692850 | 1 |
| TRIM34 | ENm009 | 11 | 5597571 | 5622205 | 1 |
| TRIM6 | ENm009 | 11 | 5573916 | 5590765 | 1 |
| TRIM6-TRIM34 | ENm009 | 11 | 5574532 | 5622205 | 1 |
| TSEN34 | ENm007 | 19 | 59385602 | 59389334 | 1 |
| TSPAN32 | ENm011 | 11 | 2279804 | 2296007 | 1 |
| TUFT1 | ENr231 | 1 | 148325855 | 148369133 | 1 |
| U52112.12 | ENm006 | X | 152666975 | 152675292 | 1 |
| UQCRQ | ENm002 | 5 | 132230152 | 132231623 | 1 |
| WRB | ENr133 | 21 | 39674041 | 39691686 | 1 |
| XX-FW81657B9.5 | ENm006 | X | 153269077 | 153269823 | 1 |
| YWHAH | ENm004 | 22 | 30665002 | 30678145 | 1 |
| Z69890.1 | ENm008 | 16 | 221135 | 221473 | 1 |
| Z84721.2 | ENm008 | 16 | 153122 | 155156 | 1 |
| Z84721.4 | ENm008 | 16 | 158679 | 159334 | 1 |
| Z84723.1 | ENm008 | 16 | 12911 | 15124 | 1 |
| Z84812.1 | ENm008 | 16 | 283 | 4091 | 1 |
| Z84812.3 | ENm008 | 16 | 1692 | 3352 | 1 |
| Z97634.3 | ENm008 | 16 | 376765 | 377235 | 1 |
| Z97634.5 | ENm008 | 16 | 372099 | 382962 | 1 |
| ZNF259 | ENm003 | 11 | 116153647 | 116163977 | -1 |
| ZNF687 | ENr231 | 1 | 148067168 | 148077455 | 1 |

Supplementary Table S.3

| **Gene ID** | **Region** | **Chr** | **Start** | **End** | **Strand** |
| --- | --- | --- | --- | --- | --- |
| AC002064.6 | ENm013 | 7 | 89609189 | 89618060 | 1 |
| AC005592.1 | ENr212 | 5 | 142105350 | 142120748 | 1 |
| AC006293.1 | ENm007 | 19 | 59912199 | 59916547 | 1 |
| AC006293.3 | ENm007 | 19 | 59913804 | 59916768 | 1 |
| AC006985.5 | ENr131 | 2 | 234466371 | 234524070 | 1 |
| AC008440.10 | ENm007 | 19 | 59059830 | 59061207 | 1 |
| AC008746.10 | ENm007 | 19 | 59582906 | 59583377 | 1 |
| AC008746.9 | ENm007 | 19 | 59724719 | 59725027 | 1 |
| AC008940.1 | ENr221 | 5 | 56102384 | 56103881 | 1 |
| AC009892.2 | ENm007 | 19 | 59755066 | 59755352 | 1 |
| AC009892.5 | ENm007 | 19 | 59811150 | 59811656 | 1 |
| AC009892.8 | ENm007 | 19 | 59851206 | 59852115 | 1 |
| AC009892.9 | ENm007 | 19 | 59855348 | 59855493 | 1 |
| AC009955.5 | ENr331 | 2 | 220240957 | 220261749 | -1 |
| AC011330.12 | ENr233 | 15 | 41678883 | 41684392 | 1 |
| AC011501.2 | ENm007 | 19 | 59958288 | 59970636 | 1 |
| AC011501.4 | ENm007 | 19 | 59989728 | 59993583 | 1 |
| AC011515.2 | ENm007 | 19 | 59874495 | 59875855 | 1 |
| AC011515.3 | ENm007 | 19 | 59900196 | 59904496 | 1 |
| AC023590.1 | ENr321 | 8 | 119363663 | 119377115 | 1 |
| AC051649.12 | ENm011 | 11 | 1841984 | 1844474 | 1 |
| AC079630.2 | ENr123 | 12 | 38836309 | 38847777 | 1 |
| AC092402.4 | ENm006 | X | 152982660 | 153003603 | -1 |
| AC092402.5 | ENm006 | X | 153006120 | 153019603 | 1 |
| AC098784.1 | ENm007 | 19 | 59907543 | 59907826 | 1 |
| AC104389.16 | ENm009 | 11 | 5182848 | 5185115 | 1 |
| AC114812.10 | ENr131 | 2 | 234441264 | 234444303 | 1 |
| AC114812.9 | ENr131 | 2 | 234429599 | 234432604 | 1 |
| AF064861.94 | ENr133 | 21 | 39610956 | 39617015 | 1 |
| AFF4 | ENm002 | 5 | 132238971 | 132267996 | -1 |
| AL163953.1 | ENr311 | 14 | 53149193 | 53149874 | 1 |
| AP000269.3 | ENm005 | 21 | 32747874 | 32752640 | 1 |
| AP000282.3 | ENm005 | 21 | 33253067 | 33254745 | 1 |
| AP000288.2 | ENm005 | 21 | 33352006 | 33359160 | 1 |
| AP000569.2 | ENm005 | 21 | 34243101 | 34258131 | 1 |
| AP001092.5 | ENr332 | 11 | 64175344 | 64180360 | 1 |
| AP002856.7 | ENr312 | 11 | 130609644 | 130626922 | 1 |
| AP003774.5 | ENr332 | 11 | 63952210 | 63956719 | 1 |
| AP005273.1 | ENr332 | 11 | 64024902 | 64029435 | 1 |
| AP006288.1 | ENr332 | 11 | 64055093 | 64057019 | 1 |
| APOC3 | ENm003 | 11 | 116205633 | 116208999 | 1 |
| ARHGAP26 | ENr212 | 5 | 142130134 | 142374156 | 1 |
| C21orf55 | ENm005 | 21 | 33779708 | 33785898 | -1 |
| C21orf62 | ENm005 | 21 | 33087747 | 33107924 | -1 |
| C21orf63 | ENm005 | 21 | 32706186 | 32809571 | 1 |
| C9orf106 | ENr232 | 9 | 129162850 | 129166739 | 1 |
| CACNG8 | ENm007 | 19 | 59158107 | 59185282 | 1 |
| CKMT1 | ENr233 | 15 | 41774623 | 41775340 | 1 |
| CKMT1A | ENr233 | 15 | 41772377 | 41778713 | 1 |
| CKMT1B | ENr233 | 15 | 41672545 | 41678897 | 1 |
| CTAG1A | ENm006 | X | 153377112 | 153378780 | 1 |
| CTGF | ENr222 | 6 | 132311010 | 132314207 | -1 |
| CXorf2 | ENm006 | X | 153019778 | 153044644 | -1 |
| CXorf2B | ENm006 | X | 152945530 | 152966482 | -1 |
| CYP4A22 | SCL | 1 | 47315128 | 47327434 | 1 |
| CYP4Z1 | SCL | 1 | 47245181 | 47296012 | 1 |
| F7 | ENr132 | 13 | 112808107 | 112822997 | 1 |
| FSCN3 | ENm014 | 7 | 126825415 | 126835804 | 1 |
| GRM8 | ENm014 | 7 | 125672608 | 126487300 | -1 |
| H2AFB1 | ENm006 | X | 153676952 | 153677538 | 1 |
| HOXA1 | ENm010 | 7 | 26905853 | 26908834 | -1 |
| HOXA2 | ENm010 | 7 | 26913217 | 26915546 | -1 |
| HOXA5 | ENm010 | 7 | 26953912 | 26956704 | -1 |
| HOXA6 | ENm010 | 7 | 26958256 | 26965458 | -1 |
| HOXA7 | ENm010 | 7 | 26966576 | 26970796 | -1 |
| HOXA9 | ENm010 | 7 | 26975298 | 26988040 | -1 |
| HS3ST4 | ENr211 | 16 | 25805569 | 26056511 | 1 |
| IGF2 | ENm011 | 11 | 2106919 | 2138797 | -1 |
| IGF2AS | ENm011 | 11 | 2118308 | 2126471 | 1 |
| IGLCOR22-1 | ENm004 | 22 | 30920461 | 30920776 | 1 |
| IL5 | ENm002 | 5 | 131905036 | 131920430 | -1 |
| INS | ENm011 | 11 | 2137585 | 2139148 | -1 |
| ITSN1 | ENm005 | 21 | 33936577 | 34194036 | 1 |
| KIF3A | ENm002 | 5 | 132056268 | 132101230 | -1 |
| KIR2DL1 | ENm007 | 19 | 59973076 | 59987311 | 1 |
| KIR2DL3 | ENm007 | 19 | 59941793 | 59956317 | 1 |
| KIR2DL4 | ENm007 | 19 | 60006879 | 60017785 | 1 |
| KIR3DL1 | ENm007 | 19 | 60019736 | 60023280 | 1 |
| KIR3DL3 | ENm007 | 19 | 59927797 | 59939816 | 1 |
| LAIR2 | ENm007 | 19 | 59700913 | 59713710 | 1 |
| LILRA1 | ENm007 | 19 | 59796860 | 59805368 | 1 |
| LILRA2 | ENm007 | 19 | 59776200 | 59790840 | 1 |
| LILRA3 | ENm007 | 19 | 59491667 | 59501765 | -1 |
| LILRA5 | ENm007 | 19 | 59510166 | 59516222 | -1 |
| LILRB1 | ENm007 | 19 | 59820425 | 59840792 | 1 |
| LILRB4 | ENm007 | 19 | 59847153 | 59873623 | 1 |
| LILRB5 | ENm007 | 19 | 59446076 | 59452977 | -1 |
| NCR2 | ENr334 | 6 | 41411372 | 41426604 | 1 |
| NRXN2 | ENr332 | 11 | 64130223 | 64247237 | -1 |
| OLIG1 | ENm005 | 21 | 33364321 | 33366597 | 1 |
| OLIG2 | ENm005 | 21 | 33320024 | 33323375 | 1 |
| OPN1LW | ENm006 | X | 152930593 | 152945355 | 1 |
| OPN1MW | ENm006 | X | 152969002 | 152982481 | 1 |
| OR51A7 | ENm009 | 11 | 4885177 | 4886115 | 1 |
| OR51F2 | ENm009 | 11 | 4799193 | 4800221 | 1 |
| OR51H2P | ENm009 | 11 | 4854364 | 4855269 | 1 |
| OR51L1 | ENm009 | 11 | 4976790 | 4977737 | 1 |
| OR51N1P | ENm009 | 11 | 4764561 | 4765512 | 1 |
| OR51T1 | ENm009 | 11 | 4859707 | 4860690 | 1 |
| OR52A1 | ENm009 | 11 | 5128816 | 5164189 | -1 |
| OR52E1P | ENm009 | 11 | 5047379 | 5048304 | 1 |
| OR52E3P | ENm009 | 11 | 5070483 | 5071418 | 1 |
| OR52J1P | ENm009 | 11 | 5081960 | 5082891 | 1 |
| OR52J2P | ENm009 | 11 | 5014820 | 5015758 | 1 |
| OR52J3 | ENm009 | 11 | 5024333 | 5025268 | 1 |
| OR52U1P | ENm009 | 11 | 5697097 | 5698019 | 1 |
| OR56B1 | ENm009 | 11 | 5686015 | 5715298 | 1 |
| OSTM1 | ENr323 | 6 | 108469307 | 108502639 | -1 |
| RFPL2 | ENm004 | 22 | 30910980 | 30924019 | -1 |
| RFPL3 | ENm004 | 22 | 31075427 | 31081703 | 1 |
| RGS11 | ENm008 | 16 | 258302 | 265982 | -1 |
| RP11-257K9.7 | ENr223 | 6 | 73989989 | 73992215 | -1 |
| RP11-298J23.5 | ENr334 | 6 | 41796113 | 41809749 | 1 |
| RP11-328M4.3 | ENr334 | 6 | 41578161 | 41595569 | 1 |
| RP11-344B5.3 | ENr232 | 9 | 129100896 | 129103415 | 1 |
| RP11-344B5.4 | ENr232 | 9 | 129100188 | 129101680 | 1 |
| RP11-398K22.14 | ENr223 | 6 | 74136149 | 74137350 | 1 |
| RP11-398K22.4 | ENr223 | 6 | 74129122 | 74130616 | 1 |
| RP1-149A16.11 | ENm004 | 22 | 31033633 | 31036187 | 1 |
| RP11-69I8.2 | ENr222 | 6 | 132264797 | 132283399 | 1 |
| RP11-88E10.3 | ENr132 | 13 | 112349360 | 112386813 | 1 |
| RP3-523C21.1 | ENr222 | 6 | 132494749 | 132532208 | 1 |
| SLC5A1 | ENm004 | 22 | 30763574 | 30833571 | 1 |
| SLC5A4 | ENm004 | 22 | 30939020 | 30975883 | -1 |
| SPP2 | ENr131 | 2 | 234741324 | 234767779 | 1 |
| SYNJ1 | ENm005 | 21 | 32922945 | 33022184 | -1 |
| TH | ENm011 | 11 | 2141736 | 2149684 | -1 |
| UBQLN3 | ENm009 | 11 | 5485107 | 5487792 | -1 |
| UGT1A1 | ENr131 | 2 | 234450895 | 234463946 | 1 |
| UGT1A11P | ENr131 | 2 | 234294200 | 234295048 | 1 |
| UGT1A12P | ENr131 | 2 | 234276086 | 234276938 | 1 |
| UGT1A13P | ENr131 | 2 | 234338573 | 234339673 | 1 |
| UGT1A3 | ENr131 | 2 | 234419755 | 234463946 | 1 |
| UGT1A4 | ENr131 | 2 | 234409425 | 234463946 | 1 |
| UGT1A5 | ENr131 | 2 | 234403639 | 234463946 | 1 |
| UGT1A6 | ENr131 | 2 | 234382254 | 234463947 | 1 |
| UGT1A7 | ENr131 | 2 | 234372585 | 234463946 | 1 |
| UGT1A8 | ENr131 | 2 | 234308292 | 234463957 | 1 |
| UGT1A9 | ENr131 | 2 | 234362500 | 234463947 | 1 |

Supplementary Table S.4

| **Gene ID** | **Region** | **Chr** | **Start** | **End** | **Strand** |
| --- | --- | --- | --- | --- | --- |
| AC000059.1 | ENm013 | 7 | 89905872 | 89906534 | 1 |
| AC000110.1 | ENm014 | 7 | 126529626 | 126530209 | 1 |
| AC000111.4 | ENm001 | 7 | 116721743 | 116721935 | 1 |
| AC000123.4 | ENm014 | 7 | 126584134 | 126585529 | 1 |
| AC000362.1 | ENm014 | 7 | 126214973 | 126215784 | 1 |
| AC002465.2 | ENm001 | 7 | 116534867 | 116556242 | 1 |
| AC004079.2 | ENm010 | 7 | 26861029 | 26861488 | 1 |
| AC004079.4 | ENm010 | 7 | 26836598 | 26837769 | 1 |
| AC004079.7 | ENm010 | 7 | 26908984 | 26913118 | 1 |
| AC004080.15 | ENm010 | 7 | 26981759 | 26984775 | 1 |
| AC004080.17 | ENm010 | 7 | 27014890 | 27020119 | 1 |
| AC004237.1 | ENm002 | 5 | 132052273 | 132085175 | 1 |
| AC004500.5 | ENm002 | 5 | 132238987 | 132240561 | -1 |
| AC004775.1 | ENm002 | 5 | 132111037 | 132117756 | 1 |
| AC006153.3 | ENm013 | 7 | 89614337 | 89659127 | 1 |
| AC006293.1 | ENm007 | 19 | 59912199 | 59916547 | 1 |
| AC006293.3 | ENm007 | 19 | 59913804 | 59916768 | 1 |
| AC006326.2 | ENm001 | 7 | 116497868 | 116498400 | 1 |
| AC007568.1 | ENm001 | 7 | 117116865 | 117117681 | 1 |
| AC008746.5 | ENm007 | 19 | 59649821 | 59650038 | 1 |
| AC009303.2 | ENr121 | 2 | 118487981 | 118489480 | 1 |
| AC009404.2 | ENr121 | 2 | 118307744 | 118315465 | 1 |
| AC009404.7 | ENr121 | 2 | 118304691 | 118313637 | 1 |
| AC009502.2 | ENr331 | 2 | 220388027 | 220388442 | 1 |
| AC009802.2 | ENr122 | 18 | 59767516 | 59799989 | 1 |
| AC009892.2 | ENm007 | 19 | 59755066 | 59755352 | 1 |
| AC009892.5 | ENm007 | 19 | 59811150 | 59811656 | 1 |
| AC009892.9 | ENm007 | 19 | 59855348 | 59855493 | 1 |
| AC009955.4 | ENr331 | 2 | 220233891 | 220240823 | 1 |
| AC010492.2 | ENm007 | 19 | 59424724 | 59425901 | 1 |
| AC010492.5 | ENm007 | 19 | 59463672 | 59464712 | 1 |
| AC011330.13 | ENr233 | 15 | 41721880 | 41722514 | -1 |
| AC011330.6 | ENr233 | 15 | 41728614 | 41728771 | 1 |
| AC011515.2 | ENm007 | 19 | 59874495 | 59875855 | 1 |
| AC011515.3 | ENm007 | 19 | 59900196 | 59904496 | 1 |
| AC021607.1 | ENr122 | 18 | 59890372 | 59890867 | 1 |
| AC023356.3 | ENr233 | 15 | 41955340 | 41955735 | -1 |
| AC034228.2 | ENm002 | 5 | 131375041 | 131376792 | 1 |
| AC034228.4 | ENm002 | 5 | 131308001 | 131331787 | 1 |
| AC051649.10 | ENm011 | 11 | 1868985 | 1870069 | 1 |
| AC051649.12 | ENm011 | 11 | 1841984 | 1844474 | 1 |
| AC063976.7 | ENm002 | 5 | 131563049 | 131567080 | 1 |
| AC068580.6 | ENm011 | 11 | 1738155 | 1740293 | 1 |
| AC068610.3 | ENm012 | 7 | 114312964 | 114360320 | 1 |
| AC073472.1 | ENm010 | 7 | 26734970 | 26735772 | 1 |
| AC080091.1 | ENr112 | 2 | 51648932 | 51650273 | 1 |
| AC098789.1 | ENm007 | 19 | 59463671 | 59464709 | 1 |
| AC104389.32 | ENm009 | 11 | 5340422 | 5340656 | 1 |
| AC113617.1 | ENr113 | 4 | 118639385 | 118651612 | 1 |
| AC116366.4 | ENm002 | 5 | 131774228 | 131839636 | 1 |
| AC139143.1 | ENm011 | 11 | 1770360 | 1770677 | 1 |
| ADTB1L1 | ENm004 | 22 | 30842519 | 30854011 | 1 |
| ADTB1L2 | ENm004 | 22 | 30854570 | 30854675 | 1 |
| AF064859.2 | ENr133 | 21 | 39421365 | 39421837 | 1 |
| AF129408.14 | ENr133 | 21 | 39510421 | 39511303 | 1 |
| AF277315.13 | ENm006 | X | 153446525 | 153447304 | 1 |
| AF277315.17 | ENm006 | X | 153424441 | 153425153 | -1 |
| AL162151.1 | ENr322 | 14 | 98508970 | 98509392 | -1 |
| AL162151.3 | ENr322 | 14 | 98509405 | 98509562 | 1 |
| AP000271.1 | ENm005 | 21 | 32790994 | 32791493 | 1 |
| AP000279.69 | ENm005 | 21 | 33022298 | 33037305 | 1 |
| AP000295.7 | ENm005 | 21 | 33558049 | 33560436 | -1 |
| AP000297.1 | ENm005 | 21 | 33628748 | 33629273 | 1 |
| AP000569.8 | ENm005 | 21 | 34225335 | 34271863 | 1 |
| AP000936.1 | ENm003 | 11 | 116411724 | 116411978 | -1 |
| AP001187.10 | ENr332 | 11 | 64418595 | 64438549 | -1 |
| AP001187.11 | ENr332 | 11 | 64405584 | 64405994 | 1 |
| AP001462.7 | ENr332 | 11 | 64326241 | 64326612 | 1 |
| AP003774.4 | ENr332 | 11 | 63973123 | 63975703 | 1 |
| AP006216.10 | ENm003 | 11 | 116149316 | 116150132 | 1 |
| AP006216.9 | ENm003 | 11 | 116219329 | 116332991 | -1 |
| APOA4 | ENm003 | 11 | 116196630 | 116199233 | -1 |
| ARD1 | ENm006 | X | 152715543 | 152721524 | -1 |
| ARF5 | ENm014 | 7 | 126822351 | 126825711 | 1 |
| ARHGAP26 | ENr212 | 5 | 142130134 | 142374156 | 1 |
| ASCL2 | ENm011 | 11 | 2246302 | 2248759 | -1 |
| ATF4P | ENm006 | X | 153372894 | 153373966 | 1 |
| ATP11A | ENr132 | 13 | 112392645 | 112589484 | 1 |
| ATP6AP1 | ENm006 | X | 153177832 | 153183640 | 1 |
| AVPR2 | ENm006 | X | 152688833 | 152693468 | 1 |
| AXIN1 | ENm008 | 16 | 277442 | 342675 | -1 |
| BIRC4 | ENr324 | X | 122719110 | 122773365 | 1 |
| BRCC3 | ENm006 | X | 153863400 | 153915054 | 1 |
| BUD13 | ENm003 | 11 | 116124097 | 116148915 | -1 |
| BXDC1P | ENr333 | 20 | 33804151 | 33804929 | 1 |
| C16orf33 | ENm008 | 16 | 43011 | 47670 | 1 |
| C16orf35 | ENm008 | 16 | 74274 | 128860 | -1 |
| C20orf173 | ENr333 | 20 | 33578214 | 33580892 | -1 |
| C20orf52 | ENr333 | 20 | 33750609 | 33752321 | 1 |
| C21orf119 | ENm005 | 21 | 32687311 | 32688141 | 1 |
| C21orf120 | ENm005 | 21 | 33084856 | 33085687 | -1 |
| C21orf59 | ENm005 | 21 | 32886261 | 32907048 | -1 |
| C5orf35 | ENr221 | 5 | 56240845 | 56248933 | 1 |
| C6orf148 | ENr223 | 6 | 73975315 | 74076660 | -1 |
| C6orf150 | ENr223 | 6 | 74179960 | 74218721 | -1 |
| C6orf49 | ENr334 | 6 | 41856066 | 41865858 | 1 |
| CAPZA2 | ENm001 | 7 | 116045076 | 116153267 | 1 |
| CAV2 | ENm001 | 7 | 115522079 | 115742547 | 1 |
| CCDC93 | ENr121 | 2 | 118389285 | 118487940 | -1 |
| CEP250 | ENr333 | 20 | 33506400 | 33563219 | 1 |
| CGN | ENr231 | 1 | 148296060 | 148324242 | 1 |
| CMPK | SCL | 1 | 47511502 | 47556532 | 1 |
| CNOT3 | ENm007 | 19 | 59333257 | 59351259 | 1 |
| CPNE1 | ENr333 | 20 | 33677368 | 33725881 | -1 |
| CRYZL1 | ENm005 | 21 | 33883518 | 33938103 | -1 |
| CSF2 | ENm002 | 5 | 131437383 | 131439759 | 1 |
| CTA-415G2.2 | ENm004 | 22 | 31735520 | 31736001 | 1 |
| CTSD | ENm011 | 11 | 1725474 | 1741799 | -1 |
| CXorf12 | ENm006 | X | 152758626 | 152769494 | 1 |
| CXorf52 | ENm006 | X | 153363184 | 153363896 | 1 |
| DDX18 | ENr121 | 2 | 118288457 | 118306186 | 1 |
| DECR2 | ENm008 | 16 | 391828 | 402489 | 1 |
| DEPDC5 | ENm004 | 22 | 30474499 | 30627556 | 1 |
| DKC1 | ENm006 | X | 153554740 | 153569669 | 1 |
| DOLPP1 | ENr232 | 9 | 128922934 | 128932272 | 1 |
| DONSON | ENm005 | 21 | 33869654 | 34206506 | -1 |
| DRG1 | ENm004 | 22 | 30131553 | 30154993 | 1 |
| DSCR2 | ENr133 | 21 | 39468566 | 39477648 | -1 |
| EEF1A1 | ENr223 | 6 | 74282195 | 74288345 | -1 |
| EHD1 | ENr332 | 11 | 64375691 | 64412345 | -1 |
| EIF4ENIF1 | ENm004 | 22 | 30159904 | 30216649 | -1 |
| ELL3 | ENr233 | 15 | 41852091 | 41878624 | -1 |
| EMD | ENm006 | X | 153128405 | 153130731 | 1 |
| ERGIC3 | ENr333 | 20 | 33593185 | 33608820 | 1 |
| EVX1 | ENm010 | 7 | 27055525 | 27060692 | 1 |
| F10 | ENr132 | 13 | 112825130 | 112832622 | 1 |
| F8 | ENm006 | X | 153627768 | 153818920 | -1 |
| F8A1 | ENm006 | X | 153678329 | 153680041 | 1 |
| FAM3A | ENm006 | X | 153298195 | 153308271 | -1 |
| FAM50A | ENm006 | X | 153236209 | 153242707 | 1 |
| FAM73B | ENr232 | 9 | 128878455 | 128913916 | 1 |
| FAM83C | ENr333 | 20 | 33336949 | 33343640 | -1 |
| FBXO7 | ENm004 | 22 | 31195218 | 31219371 | 1 |
| FER1L4 | ENr333 | 20 | 33609922 | 33658899 | -1 |
| FLNA | ENm006 | X | 153097742 | 153123842 | -1 |
| FOXP4 | ENr334 | 6 | 41622143 | 41678101 | 1 |
| FRS3 | ENr334 | 6 | 41845893 | 41856183 | -1 |
| FUNDC2 | ENm006 | X | 153817960 | 153852283 | 1 |
| FZD1 | ENm013 | 7 | 90538331 | 90542764 | 1 |
| GAB3 | ENm006 | X | 153467234 | 153543563 | -1 |
| GART | ENm005 | 21 | 33798109 | 33837668 | -1 |
| GDF5 | ENr333 | 20 | 33484560 | 33505983 | -1 |
| GDF9 | ENm002 | 5 | 132224773 | 132230229 | -1 |
| GMPPA | ENr331 | 2 | 220189095 | 220197216 | 1 |
| GTPBP10 | ENm013 | 7 | 89620631 | 89665421 | 1 |
| HBG2 | ENm009 | 11 | 5230997 | 5623596 | -1 |
| HCFC1 | ENm006 | X | 152733852 | 152758106 | -1 |
| HMGN1 | ENr133 | 21 | 39636112 | 39643444 | -1 |
| HOXA1 | ENm010 | 7 | 26905853 | 26908834 | -1 |
| HOXA10 | ENm010 | 7 | 26983451 | 26993121 | -1 |
| HOXA11 | ENm010 | 7 | 26994017 | 26998083 | -1 |
| HOXA11S | ENm010 | 7 | 26998268 | 27002153 | 1 |
| HOXA13 | ENm010 | 7 | 27008263 | 27012966 | -1 |
| HOXA3 | ENm010 | 7 | 26919044 | 26953068 | -1 |
| HOXA4 | ENm010 | 7 | 26941367 | 26943659 | -1 |
| HOXA5 | ENm010 | 7 | 26953912 | 26956704 | -1 |
| HOXA9 | ENm010 | 7 | 26975298 | 26988040 | -1 |
| HYPK | ENr233 | 15 | 41875633 | 41882534 | 1 |
| IER5L | ENr232 | 9 | 129017386 | 129020096 | -1 |
| IFNAR1 | ENm005 | 21 | 33618653 | 33654039 | 1 |
| IFNAR2 | ENm005 | 21 | 33524077 | 33559840 | 1 |
| IFNGR2 | ENm005 | 21 | 33679170 | 33773526 | 1 |
| IGLCOR22-1 | ENm004 | 22 | 30920461 | 30920776 | 1 |
| IL10RB | ENm005 | 21 | 33542891 | 33591410 | 1 |
| IL13 | ENm002 | 5 | 132019855 | 132024702 | 1 |
| IL3 | ENm002 | 5 | 131424122 | 131426797 | 1 |
| INHA | ENr331 | 2 | 220259390 | 220265941 | 1 |
| IRF1 | ENm002 | 5 | 131845201 | 131854390 | -1 |
| ITFG3 | ENm008 | 16 | 224547 | 258972 | 1 |
| ITGB4BP | ENr333 | 20 | 33330129 | 33336203 | -1 |
| ITSN1 | ENm005 | 21 | 33936577 | 34194036 | 1 |
| KCNQ5 | ENr223 | 6 | 73808381 | 73965296 | 1 |
| LACE1 | ENr323 | 6 | 108722792 | 108829952 | 1 |
| LAGE3 | ENm006 | X | 153269813 | 153271301 | -1 |
| LAIR1 | ENm007 | 19 | 59557075 | 59573978 | -1 |
| LAIR2 | ENm007 | 19 | 59700913 | 59713710 | 1 |
| LEAP2 | ENm002 | 5 | 132235914 | 132238638 | 1 |
| LENG1 | ENm007 | 19 | 59350712 | 59355433 | -1 |
| LENG4 | ENm007 | 19 | 59368922 | 59385479 | -1 |
| LENG8 | ENm007 | 19 | 59651878 | 59665030 | 1 |
| LENG9 | ENm007 | 19 | 59664789 | 59666707 | -1 |
| LILRA1 | ENm007 | 19 | 59796860 | 59805368 | 1 |
| LILRA2 | ENm007 | 19 | 59776200 | 59790840 | 1 |
| LILRA3 | ENm007 | 19 | 59491667 | 59501765 | -1 |
| LILRA4 | ENm007 | 19 | 59536269 | 59542234 | -1 |
| LILRA6 | ENm007 | 19 | 59432281 | 59438943 | -1 |
| LILRB1 | ENm007 | 19 | 59820425 | 59840792 | 1 |
| LILRB2 | ENm007 | 19 | 59469488 | 59476852 | -1 |
| LSP1 | ENm011 | 11 | 1830777 | 1870074 | 1 |
| LUC7L | ENm008 | 16 | 178970 | 219464 | -1 |
| MAP3K1 | ENr221 | 5 | 56147159 | 56227737 | 1 |
| MAP4K2 | ENr332 | 11 | 64313186 | 64327290 | -1 |
| MEN1 | ENr332 | 11 | 64327565 | 64335343 | -1 |
| MFAP1 | ENr233 | 15 | 41883983 | 41904293 | -1 |
| MMP24 | ENr333 | 20 | 33305670 | 33328216 | 1 |
| MPG | ENm008 | 16 | 67007 | 75853 | 1 |
| MPP1 | ENm006 | X | 153570664 | 153612987 | -1 |
| MRPL23 | ENm011 | 11 | 1925085 | 1962329 | 1 |
| MRPL28 | ENm008 | 16 | 356929 | 360570 | -1 |
| MTCP1 | ENm006 | X | 153853602 | 153939917 | -1 |
| MYADM | ENm007 | 19 | 59061290 | 59071504 | 1 |
| NDUFA3 | ENm007 | 19 | 59297849 | 59306711 | 1 |
| NFS1 | ENr333 | 20 | 33720025 | 33750696 | -1 |
| NME4 | ENm008 | 16 | 386727 | 402489 | 1 |
| NUP188 | ENr232 | 9 | 128783312 | 128848930 | 1 |
| OLIG1 | ENm005 | 21 | 33364321 | 33366597 | 1 |
| OR51I1 | ENm009 | 11 | 5418377 | 5419321 | -1 |
| OR56B1 | ENm009 | 11 | 5686015 | 5715298 | 1 |
| OSCAR | ENm007 | 19 | 59289746 | 59297813 | -1 |
| OSTM1 | ENr323 | 6 | 108469307 | 108502639 | -1 |
| PDIA2 | ENm008 | 16 | 273154 | 277217 | 1 |
| PDIA3 | ENr233 | 15 | 41825883 | 41852770 | 1 |
| PDLIM4 | ENm002 | 5 | 131621264 | 131637047 | 1 |
| PFTK1 | ENm013 | 7 | 89740390 | 90484557 | 1 |
| PIK4CB | ENr231 | 1 | 148077487 | 148113265 | -1 |
| PIP5K1A | ENr231 | 1 | 147983499 | 148035086 | 1 |
| PISD | ENm004 | 22 | 30339032 | 30382973 | -1 |
| PLXNA3 | ENm006 | X | 153250326 | 153265694 | 1 |
| POGZ | ENr231 | 1 | 148188274 | 148245015 | -1 |
| POLR3K | ENm008 | 16 | 36408 | 43629 | -1 |
| PPP2R4 | ENr232 | 9 | 128952784 | 128990780 | 1 |
| PRPF31 | ENm007 | 19 | 59310650 | 59326956 | 1 |
| PSMB4 | ENr231 | 1 | 148185084 | 148187494 | 1 |
| PSMD4 | ENr231 | 1 | 148040253 | 148053029 | 1 |
| RAB11FIP3 | ENm008 | 16 | 415621 | 495629 | 1 |
| RASGRP2 | ENr332 | 11 | 64250960 | 64269505 | -1 |
| RBM12 | ENr333 | 20 | 33700262 | 33716253 | -1 |
| RENBP | ENm006 | X | 152721564 | 152730991 | -1 |
| RFPL3S | ENm004 | 22 | 31080448 | 31091618 | -1 |
| RFX5 | ENr231 | 1 | 148126190 | 148132907 | -1 |
| RP11-115M6.4 | ENm006 | X | 153608306 | 153609501 | -1 |
| RP1-111B22.2 | ENr323 | 6 | 108432255 | 108433466 | -1 |
| RP11-126K1.8 | ENr231 | 1 | 148126198 | 148127516 | 1 |
| RP11-144L2.1 | ENr132 | 13 | 112399147 | 112419991 | 1 |
| RP11-223E19.1 | ENr111 | 13 | 29900129 | 29900593 | 1 |
| RP11-247A12.1 | ENr232 | 9 | 128939688 | 128942100 | 1 |
| RP11-247A12.6 | ENr232 | 9 | 128936628 | 128952564 | -1 |
| RP11-247I13.3 | ENm004 | 22 | 30280024 | 30280480 | 1 |
| RP1-128O3.5 | ENr323 | 6 | 108680112 | 108680298 | 1 |
| RP1-128O3.6 | ENr323 | 6 | 108745977 | 108746302 | 1 |
| RP11-298J23.7 | ENr334 | 6 | 41863379 | 41865615 | 1 |
| RP11-328M4.2 | ENr334 | 6 | 41570570 | 41624338 | -1 |
| RP11-374F3.2 | ENr111 | 13 | 29768285 | 29768873 | 1 |
| RP11-380M3.3 | ENr223 | 6 | 73821542 | 73822302 | 1 |
| RP11-398K22.10 | ENr223 | 6 | 74089282 | 74089895 | 1 |
| RP11-398K22.11 | ENr223 | 6 | 74135000 | 74161578 | -1 |
| RP11-398K22.12 | ENr223 | 6 | 74029660 | 74067846 | 1 |
| RP11-398K22.13 | ENr223 | 6 | 74056848 | 74057707 | 1 |
| RP11-398K22.6 | ENr223 | 6 | 74093969 | 74095462 | 1 |
| RP11-398K22.9 | ENr223 | 6 | 74058407 | 74059722 | 1 |
| RP1-149A16.15 | ENm004 | 22 | 31097224 | 31097677 | 1 |
| RP1-149A16.16 | ENm004 | 22 | 31107210 | 31107410 | 1 |
| RP1-149A16.17 | ENm004 | 22 | 31104328 | 31106173 | 1 |
| RP1-149M18.3 | ENr334 | 6 | 41457109 | 41457549 | -1 |
| RP11-74C1.2 | ENr231 | 1 | 148342996 | 148343490 | 1 |
| RP1-196A12.1 | ENm008 | 16 | 441406 | 443463 | 1 |
| RP11-98F14.4 | ENr132 | 13 | 112832732 | 112834370 | 1 |
| RP1-248E1.2 | ENr222 | 6 | 132634815 | 132636165 | 1 |
| RP1-90G24.10 | ENm004 | 22 | 30925657 | 30990208 | 1 |
| RP1-90G24.5 | ENm004 | 22 | 30989923 | 30993652 | 1 |
| RP1-90G24.6 | ENm004 | 22 | 30993962 | 30997728 | 1 |
| RP3-466I7.1 | ENr323 | 6 | 108815410 | 108815978 | 1 |
| RP3-477O4.15 | ENr333 | 20 | 33564811 | 33568775 | 1 |
| RP4-614O4.5 | ENr333 | 20 | 33307257 | 33331261 | -1 |
| RP4-696P19.2 | ENr334 | 6 | 41742623 | 41743366 | 1 |
| RP5-931E15.2 | ENr324 | X | 122592560 | 122593680 | 1 |
| RP5-931E15.3 | ENr324 | X | 122624687 | 122625072 | 1 |
| RP5-931E15.4 | ENr324 | X | 122644395 | 122644837 | 1 |
| RPL10 | ENm006 | X | 153147247 | 153151528 | 1 |
| RPL37P1 | ENr333 | 20 | 33639661 | 33639944 | 1 |
| RPS17P4 | ENm004 | 22 | 30760032 | 30760438 | 1 |
| SERF2 | ENr233 | 15 | 41856578 | 41882080 | 1 |
| SERPINB10 | ENr122 | 18 | 59715389 | 59754326 | 1 |
| SERPINB2 | ENr122 | 18 | 59689907 | 59722105 | 1 |
| SERPINB8 | ENr122 | 18 | 59788140 | 59823259 | 1 |
| SF1 | ENr332 | 11 | 64288655 | 64302835 | -1 |
| SFI1 | ENm004 | 22 | 30209229 | 30339092 | 1 |
| SH3GLB2 | ENr232 | 9 | 128848870 | 128870137 | -1 |
| SIL | SCL | 1 | 47427870 | 47491840 | -1 |
| SLC10A3 | ENm006 | X | 153279350 | 153282707 | -1 |
| SLC22A4 | ENm002 | 5 | 131658036 | 131707799 | 1 |
| SLC22A5 | ENm002 | 5 | 131733344 | 131759206 | 1 |
| SLC4A3 | ENr331 | 2 | 220317793 | 220332208 | 1 |
| SNX27 | ENr231 | 1 | 148397615 | 148468995 | 1 |
| SNX3 | ENr323 | 6 | 108639120 | 108689158 | -1 |
| SON | ENm005 | 21 | 33836795 | 33871658 | 1 |
| SPAG4 | ENr333 | 20 | 33667229 | 33672386 | 1 |
| ST7 | ENm001 | 7 | 116187333 | 116464109 | 1 |
| ST7OT4 | ENm001 | 7 | 116187905 | 116202580 | 1 |
| STAG2 | ENr324 | X | 122819598 | 122962042 | 1 |
| STK11IP | ENr331 | 2 | 220288088 | 220306679 | 1 |
| TAL1 | SCL | 1 | 47393984 | 47409913 | -1 |
| TAZ | ENm006 | X | 153160702 | 153170913 | 1 |
| TES | ENm001 | 7 | 115444499 | 115492789 | 1 |
| TFPT | ENm007 | 19 | 59302133 | 59310868 | -1 |
| TMC4 | ENm007 | 19 | 59355659 | 59368757 | -1 |
| TMEM15 | ENr232 | 9 | 128787364 | 128789453 | -1 |
| TMEM50B | ENm005 | 21 | 33726663 | 33775370 | -1 |
| TMEM8 | ENm008 | 16 | 360775 | 377115 | -1 |
| TNNI2 | ENm011 | 11 | 1816796 | 1819485 | 1 |
| TP53BP1 | ENr233 | 15 | 41520750 | 41590219 | -1 |
| TRIM22 | ENm009 | 11 | 5667496 | 5692850 | 1 |
| TRIM34 | ENm009 | 11 | 5597571 | 5622205 | 1 |
| TRIM6-TRIM34 | ENm009 | 11 | 5574532 | 5622205 | 1 |
| TSEN34 | ENm007 | 19 | 59385602 | 59389334 | 1 |
| TSPAN32 | ENm011 | 11 | 2279804 | 2296007 | 1 |
| TUFT1 | ENr231 | 1 | 148325855 | 148369133 | 1 |
| U52112.12 | ENm006 | X | 152666975 | 152675292 | 1 |
| UQCRQ | ENm002 | 5 | 132230152 | 132231623 | 1 |
| WDR76 | ENr233 | 15 | 41906454 | 41947910 | 1 |
| WRB | ENr133 | 21 | 39674041 | 39691686 | 1 |
| XX-FW81657B9.5 | ENm006 | X | 153269077 | 153269823 | 1 |
| XX-FW83563B9.5 | ENm006 | X | 153146067 | 153148005 | -1 |
| YWHAH | ENm004 | 22 | 30665002 | 30678145 | 1 |
| Z69890.1 | ENm008 | 16 | 221135 | 221473 | 1 |
| Z84812.1 | ENm008 | 16 | 283 | 4091 | 1 |
| Z84812.2 | ENm008 | 16 | 4044 | 9453 | -1 |
| Z84812.3 | ENm008 | 16 | 1692 | 3352 | 1 |
| Z97634.3 | ENm008 | 16 | 376765 | 377235 | 1 |
| Z97634.5 | ENm008 | 16 | 372099 | 382962 | 1 |
| ZNF259 | ENm003 | 11 | 116153647 | 116163977 | -1 |
| ZNF687 | ENr231 | 1 | 148067168 | 148077455 | 1 |
| ZNF800 | ENm014 | 7 | 126580796 | 126665930 | -1 |

Supplementary Table S.5

| **Gene ID** | **Region** | **Chr** | **Start** | **End** | **Strand** |
| --- | --- | --- | --- | --- | --- |
| AC000123.2 | ENm014 | 7 | 126593791 | 126598765 | -1 |
| AC000123.3 | ENm014 | 7 | 126583839 | 126584299 | -1 |
| AC000124.1 | ENm014 | 7 | 126710889 | 126719810 | 1 |
| AC000374.1 | ENm014 | 7 | 125745141 | 125746885 | 1 |
| AC004009.1 | ENm010 | 7 | 27193422 | 27194066 | 1 |
| AC004009.2 | ENm010 | 7 | 27225024 | 27225751 | 1 |
| AC004009.3 | ENm010 | 7 | 27174703 | 27222798 | 1 |
| AC004041.2 | ENm002 | 5 | 131994181 | 132027864 | -1 |
| AC004240.2 | ENm001 | 7 | 116923719 | 116949977 | 1 |
| AC004500.4 | ENm002 | 5 | 132255409 | 132267996 | -1 |
| AC005592.2 | ENr212 | 5 | 141889745 | 142031751 | 1 |
| AC008599.2 | ENm002 | 5 | 131708274 | 131712333 | -1 |
| AC008940.1 | ENr221 | 5 | 56102384 | 56103881 | 1 |
| AC009158.1 | ENr211 | 16 | 26236969 | 26253251 | 1 |
| AC009404.5 | ENr121 | 2 | 118333234 | 118377145 | 1 |
| AC009502.3 | ENr331 | 2 | 220420338 | 220427970 | 1 |
| AC009892.8 | ENm007 | 19 | 59851206 | 59852115 | 1 |
| AC009955.5 | ENr331 | 2 | 220240957 | 220261749 | -1 |
| AC011330.12 | ENr233 | 15 | 41678883 | 41684392 | 1 |
| AC011501.2 | ENm007 | 19 | 59958288 | 59970636 | 1 |
| AC011501.4 | ENm007 | 19 | 59989728 | 59993583 | 1 |
| AC015933.2 | ENr213 | 18 | 23788482 | 23797650 | 1 |
| AC016644.1 | ENr221 | 5 | 56272892 | 56278737 | 1 |
| AC023590.1 | ENr321 | 8 | 119363663 | 119377115 | 1 |
| AC051649.13 | ENm011 | 11 | 1973004 | 1973369 | 1 |
| AC074021.1 | ENm001 | 7 | 115521386 | 115565782 | 1 |
| AC092402.5 | ENm006 | X | 153006120 | 153019603 | 1 |
| AC098784.1 | ENm007 | 19 | 59907543 | 59907826 | 1 |
| AC104389.16 | ENm009 | 11 | 5182848 | 5185115 | 1 |
| AC104389.19 | ENm009 | 11 | 5219927 | 5221344 | -1 |
| AC113188.2 | ENr321 | 8 | 119270876 | 119271391 | -1 |
| AC114812.10 | ENr131 | 2 | 234441264 | 234444303 | 1 |
| AC114812.5 | ENr131 | 2 | 234444952 | 234445992 | -1 |
| AC114812.6 | ENr131 | 2 | 234433397 | 234434662 | -1 |
| AC114812.9 | ENr131 | 2 | 234429599 | 234432604 | 1 |
| AC132217.4 | ENm011 | 11 | 2106927 | 2108043 | -1 |
| AFF4 | ENm002 | 5 | 132238971 | 132267996 | -1 |
| ANKRD43 | ENm002 | 5 | 132177179 | 132180389 | 1 |
| AP000269.3 | ENm005 | 21 | 32747874 | 32752640 | 1 |
| AP000282.3 | ENm005 | 21 | 33253067 | 33254745 | 1 |
| AP000288.2 | ENm005 | 21 | 33352006 | 33359160 | 1 |
| AP002856.5 | ENr312 | 11 | 130628528 | 130675877 | 1 |
| AP002856.7 | ENr312 | 11 | 130609644 | 130626922 | 1 |
| AP003774.5 | ENr332 | 11 | 63952210 | 63956719 | 1 |
| AP005273.1 | ENr332 | 11 | 64024902 | 64029435 | 1 |
| AP006288.1 | ENr332 | 11 | 64055093 | 64057019 | 1 |
| ASZ1 | ENm001 | 7 | 116597228 | 116662129 | -1 |
| BCL11B | ENr322 | 14 | 98705378 | 98807576 | -1 |
| C21orf55 | ENm005 | 21 | 33779708 | 33785898 | -1 |
| C21orf63 | ENm005 | 21 | 32706186 | 32809571 | 1 |
| C21orf87 | ENr133 | 21 | 39607758 | 39608757 | -1 |
| C9orf106 | ENr232 | 9 | 129162850 | 129166739 | 1 |
| CACNG6 | ENm007 | 19 | 59187355 | 59207736 | 1 |
| CACNG7 | ENm007 | 19 | 59104402 | 59139008 | 1 |
| CACNG8 | ENm007 | 19 | 59158107 | 59185282 | 1 |
| CDC42EP5 | ENm007 | 19 | 59668023 | 59676224 | -1 |
| CKMT1 | ENr233 | 15 | 41774623 | 41775340 | 1 |
| CKMT1A | ENr233 | 15 | 41772377 | 41778713 | 1 |
| CKMT1B | ENr233 | 15 | 41672545 | 41678897 | 1 |
| CTAG1A | ENm006 | X | 153377112 | 153378780 | 1 |
| CTAG1B | ENm006 | X | 153409570 | 153411238 | -1 |
| CTAG2 | ENm006 | X | 153443951 | 153445558 | -1 |
| CTGF | ENr222 | 6 | 132311010 | 132314207 | -1 |
| CYP4A22 | SCL | 1 | 47315128 | 47327434 | 1 |
| CYP4Z1 | SCL | 1 | 47245181 | 47296012 | 1 |
| DDX43 | ENr223 | 6 | 74161193 | 74184014 | 1 |
| F7 | ENr132 | 13 | 112808107 | 112822997 | 1 |
| FOXP2 | ENm012 | 7 | 113600512 | 113927779 | 1 |
| FSCN3 | ENm014 | 7 | 126825415 | 126835804 | 1 |
| GRM8 | ENm014 | 7 | 125672608 | 126487300 | -1 |
| H19 | ENm011 | 11 | 1972983 | 1979277 | -1 |
| HBA2 | ENm008 | 16 | 162847 | 163710 | 1 |
| HNT | ENr312 | 11 | 130745584 | 131036336 | 1 |
| HOXA2 | ENm010 | 7 | 26913217 | 26915546 | -1 |
| HS3ST4 | ENr211 | 16 | 25805569 | 26056511 | 1 |
| IGF2AS | ENm011 | 11 | 2118308 | 2126471 | 1 |
| IL4 | ENm002 | 5 | 132037578 | 132046268 | 1 |
| IL5 | ENm002 | 5 | 131905036 | 131920430 | -1 |
| KIF3A | ENm002 | 5 | 132056268 | 132101230 | -1 |
| KIR2DL1 | ENm007 | 19 | 59973076 | 59987311 | 1 |
| KIR2DL3 | ENm007 | 19 | 59941793 | 59956317 | 1 |
| KIR2DL4 | ENm007 | 19 | 60006879 | 60017785 | 1 |
| KIR3DL1 | ENm007 | 19 | 60019736 | 60023280 | 1 |
| KIR3DL3 | ENm007 | 19 | 59927797 | 59939816 | 1 |
| LILRA5 | ENm007 | 19 | 59510166 | 59516222 | -1 |
| LILRB5 | ENm007 | 19 | 59446076 | 59452977 | -1 |
| LRRK2 | ENr123 | 12 | 38876814 | 39049355 | 1 |
| MAP1A | ENr233 | 15 | 41590449 | 41611111 | 1 |
| MDFI | ENr334 | 6 | 41712599 | 41729963 | 1 |
| MIER3 | ENr221 | 5 | 56251187 | 56303260 | -1 |
| MOXD1 | ENr222 | 6 | 132658888 | 132691427 | -1 |
| NCR2 | ENr334 | 6 | 41411372 | 41426604 | 1 |
| OPN1LW | ENm006 | X | 152930593 | 152945355 | 1 |
| OPN1MW | ENm006 | X | 152969002 | 152982481 | 1 |
| OR51A10P | ENm009 | 11 | 5446293 | 5447229 | -1 |
| OR51B4 | ENm009 | 11 | 5278821 | 5279803 | -1 |
| OR51B5 | ENm009 | 11 | 5320393 | 5321331 | -1 |
| OR51F2 | ENm009 | 11 | 4799193 | 4800221 | 1 |
| OR51H2P | ENm009 | 11 | 4854364 | 4855269 | 1 |
| OR51I2 | ENm009 | 11 | 5431296 | 5432234 | 1 |
| OR51J1 | ENm009 | 11 | 5380404 | 5381354 | 1 |
| OR51L1 | ENm009 | 11 | 4976790 | 4977737 | 1 |
| OR51N1P | ENm009 | 11 | 4764561 | 4765512 | 1 |
| OR51Q1 | ENm009 | 11 | 5400008 | 5400961 | 1 |
| OR51T1 | ENm009 | 11 | 4859707 | 4860690 | 1 |
| OR52D1 | ENm009 | 11 | 5466514 | 5467470 | 1 |
| OR52E1P | ENm009 | 11 | 5047379 | 5048304 | 1 |
| OR52J2P | ENm009 | 11 | 5014820 | 5015758 | 1 |
| OR52J3 | ENm009 | 11 | 5024333 | 5025268 | 1 |
| OR52P1P | ENm009 | 11 | 5704309 | 5705271 | 1 |
| OR52U1P | ENm009 | 11 | 5697097 | 5698019 | 1 |
| PCDH15 | ENr114 | 10 | 55232538 | 55643815 | -1 |
| PHYHD1 | ENr232 | 9 | 128768702 | 128783872 | 1 |
| RFPL3 | ENm004 | 22 | 31075427 | 31081703 | 1 |
| RGS11 | ENm008 | 16 | 258302 | 265982 | -1 |
| RP11-257K9.7 | ENr223 | 6 | 73989989 | 73992215 | -1 |
| RP1-127L4.6 | ENm004 | 22 | 30869548 | 30879864 | -1 |
| RP1-128O3.4 | ENr323 | 6 | 108703540 | 108704444 | 1 |
| RP11-328M4.3 | ENr334 | 6 | 41578161 | 41595569 | 1 |
| RP11-344B5.4 | ENr232 | 9 | 129100188 | 129101680 | 1 |
| RP11-374F3.3 | ENr111 | 13 | 29788498 | 29792041 | -1 |
| RP11-398K22.14 | ENr223 | 6 | 74136149 | 74137350 | 1 |
| RP11-398K22.4 | ENr223 | 6 | 74129122 | 74130616 | 1 |
| RP11-398K22.7 | ENr223 | 6 | 74119507 | 74120740 | -1 |
| RP11-398K22.8 | ENr223 | 6 | 74040584 | 74076810 | -1 |
| RP1-149M18.4 | ENr334 | 6 | 41481536 | 41482278 | 1 |
| RP11-69I8.2 | ENr222 | 6 | 132264797 | 132283399 | 1 |
| RP11-73M11.2 | ENr132 | 13 | 112447765 | 112457009 | -1 |
| RP1-18D14.7 | SCL | 1 | 47403490 | 47408443 | 1 |
| RP11-90M5.1 | ENr111 | 13 | 29419741 | 29422626 | 1 |
| RP3-429G5.3 | ENr323 | 6 | 108551413 | 108587290 | 1 |
| RP3-523C21.1 | ENr222 | 6 | 132494749 | 132532208 | 1 |
| SERPINB11 | ENr122 | 18 | 59465794 | 59542104 | 1 |
| SERPINB3 | ENr122 | 18 | 59473412 | 59480178 | -1 |
| SERPINB4 | ENr122 | 18 | 59455474 | 59462513 | -1 |
| SLC5A1 | ENm004 | 22 | 30763574 | 30833571 | 1 |
| SLC5A4 | ENm004 | 22 | 30939020 | 30975883 | -1 |
| SPP2 | ENr131 | 2 | 234741324 | 234767779 | 1 |
| ST7OT2 | ENm001 | 7 | 116306078 | 116380486 | -1 |
| TH | ENm011 | 11 | 2141736 | 2149684 | -1 |
| TIMP3 | ENm004 | 22 | 31522242 | 31583585 | 1 |
| TKTL1 | ENm006 | X | 153044872 | 153079548 | 1 |
| TTYH1 | ENm007 | 19 | 59618186 | 59639893 | 1 |
| UBQLN3 | ENm009 | 11 | 5485107 | 5487792 | -1 |
| UGT1A1 | ENr131 | 2 | 234450895 | 234463946 | 1 |
| UGT1A11P | ENr131 | 2 | 234294200 | 234295048 | 1 |
| UGT1A12P | ENr131 | 2 | 234276086 | 234276938 | 1 |
| UGT1A13P | ENr131 | 2 | 234338573 | 234339673 | 1 |
| UGT1A3 | ENr131 | 2 | 234419755 | 234463946 | 1 |
| UGT1A4 | ENr131 | 2 | 234409425 | 234463946 | 1 |
| UGT1A5 | ENr131 | 2 | 234403639 | 234463946 | 1 |
| UGT1A6 | ENr131 | 2 | 234382254 | 234463947 | 1 |
| UGT1A7 | ENr131 | 2 | 234372585 | 234463946 | 1 |
| UGT1A8 | ENr131 | 2 | 234308292 | 234463957 | 1 |
| UGT1A9 | ENr131 | 2 | 234362500 | 234463947 | 1 |
| WNT2 | ENm001 | 7 | 116510637 | 116557295 | -1 |
| Z84721.4 | ENm008 | 16 | 158679 | 159334 | 1 |

Supplementary Table S.6

| **Gene ID** | **Region** | **Chr** | **Start** | **End** | **Strand** |
| --- | --- | --- | --- | --- | --- |
| AC000059.1 | ENm013 | 7 | 89905872 | 89906534 | 1 |
| AC000111.3 | ENm001 | 7 | 116794739 | 116798682 | -1 |
| AC000123.2 | ENm014 | 7 | 126593791 | 126598765 | -1 |
| AC000124.1 | ENm014 | 7 | 126710889 | 126719810 | 1 |
| AC004079.2 | ENm010 | 7 | 26861029 | 26861488 | 1 |
| AC004079.4 | ENm010 | 7 | 26836598 | 26837769 | 1 |
| AC004080.16 | ENm010 | 7 | 27011435 | 27015682 | 1 |
| AC004080.17 | ENm010 | 7 | 27014890 | 27020119 | 1 |
| AC004500.5 | ENm002 | 5 | 132238987 | 132240561 | -1 |
| AC004996.2 | ENm010 | 7 | 27082213 | 27082571 | -1 |
| AC005215.1 | ENr212 | 5 | 141926450 | 141926826 | 1 |
| AC005538.1 | ENr131 | 2 | 234524063 | 234545213 | -1 |
| AC005592.1 | ENr212 | 5 | 142105350 | 142120748 | 1 |
| AC008937.3 | ENr221 | 5 | 56231626 | 56242299 | -1 |
| AC008984.6 | ENm007 | 19 | 59531357 | 59534519 | -1 |
| AC009404.5 | ENr121 | 2 | 118333234 | 118377145 | 1 |
| AC009502.1 | ENr331 | 2 | 220374258 | 220375023 | -1 |
| AC009502.2 | ENr331 | 2 | 220388027 | 220388442 | 1 |
| AC011515.3 | ENm007 | 19 | 59900196 | 59904496 | 1 |
| AC012314.8 | ENm007 | 19 | 59314704 | 59320535 | -1 |
| AC015691.7 | ENm009 | 11 | 5549054 | 5549999 | -1 |
| AC018512.3 | ENr233 | 15 | 41806409 | 41826585 | -1 |
| AC018512.6 | ENr233 | 15 | 41873653 | 41879712 | -1 |
| AC023356.3 | ENr233 | 15 | 41955340 | 41955735 | -1 |
| AC034228.3 | ENm002 | 5 | 131367185 | 131370615 | 1 |
| AC051649.13 | ENm011 | 11 | 1973004 | 1973369 | 1 |
| AC051649.6 | ENm011 | 11 | 1968903 | 1971276 | 1 |
| AC063976.6 | ENm002 | 5 | 131556579 | 131557161 | -1 |
| AC073472.1 | ENm010 | 7 | 26734970 | 26735772 | 1 |
| AC073626.1 | ENm012 | 7 | 113531119 | 113531435 | -1 |
| AC092402.5 | ENm006 | X | 153006120 | 153019603 | 1 |
| AC092661.2 | ENr113 | 4 | 118853271 | 118854933 | -1 |
| AC097463.1 | ENr112 | 2 | 52065672 | 52066152 | -1 |
| AC104389.28 | ENm009 | 11 | 5282783 | 5483459 | -1 |
| AC104389.32 | ENm009 | 11 | 5340422 | 5340656 | 1 |
| AC114812.2 | ENr131 | 2 | 234412169 | 234412712 | -1 |
| AC114973.1 | ENr221 | 5 | 56307178 | 56307809 | -1 |
| AC116366.4 | ENm002 | 5 | 131774228 | 131839636 | 1 |
| AC116366.6 | ENm002 | 5 | 131832479 | 131836635 | -1 |
| AC139143.1 | ENm011 | 11 | 1770360 | 1770677 | 1 |
| AC139143.2 | ENm011 | 11 | 1780696 | 1781902 | -1 |
| ACCN4 | ENr331 | 2 | 220204398 | 220229000 | 1 |
| ADTB1L1 | ENm004 | 22 | 30842519 | 30854011 | 1 |
| ADTB1L2 | ENm004 | 22 | 30854570 | 30854675 | 1 |
| AF064859.2 | ENr133 | 21 | 39421365 | 39421837 | 1 |
| AF121781.17 | ENr133 | 21 | 39704666 | 39722325 | 1 |
| AFF4 | ENm002 | 5 | 132238971 | 132267996 | -1 |
| AL162151.1 | ENr322 | 14 | 98508970 | 98509392 | -1 |
| AL163953.1 | ENr311 | 14 | 53149193 | 53149874 | 1 |
| AP000269.3 | ENm005 | 21 | 32747874 | 32752640 | 1 |
| AP000271.1 | ENm005 | 21 | 32790994 | 32791493 | 1 |
| AP000281.1 | ENm005 | 21 | 33135677 | 33135992 | -1 |
| AP000281.2 | ENm005 | 21 | 33138548 | 33144115 | -1 |
| AP000569.8 | ENm005 | 21 | 34225335 | 34271863 | 1 |
| AP000936.1 | ENm003 | 11 | 116411724 | 116411978 | -1 |
| AP001187.10 | ENr332 | 11 | 64418595 | 64438549 | -1 |
| AP001187.9 | ENr332 | 11 | 64415475 | 64417498 | -1 |
| AP002856.7 | ENr312 | 11 | 130609644 | 130626922 | 1 |
| AP003025.2 | ENr312 | 11 | 130877864 | 130915972 | -1 |
| AP003039.2 | ENr312 | 11 | 131033418 | 131038061 | -1 |
| AP003039.4 | ENr312 | 11 | 130921767 | 130948705 | 1 |
| AP006216.9 | ENm003 | 11 | 116219329 | 116332991 | -1 |
| APOA5 | ENm003 | 11 | 116165294 | 116168338 | -1 |
| ARD1 | ENm006 | X | 152715543 | 152721524 | -1 |
| ARF5 | ENm014 | 7 | 126822351 | 126825711 | 1 |
| ASCL2 | ENm011 | 11 | 2246302 | 2248759 | -1 |
| ATP11A | ENr132 | 13 | 112392645 | 112589484 | 1 |
| ATP5O | ENm005 | 21 | 34197628 | 34210155 | -1 |
| AVPR2 | ENm006 | X | 152688833 | 152693468 | 1 |
| AXIN1 | ENm008 | 16 | 277442 | 342675 | -1 |
| BCL11B | ENr322 | 14 | 98705378 | 98807576 | -1 |
| BUD13 | ENm003 | 11 | 116124097 | 116148915 | -1 |
| BXDC1P | ENr333 | 20 | 33804151 | 33804929 | 1 |
| C11orf21 | ENm011 | 11 | 2273452 | 2280856 | -1 |
| C16orf33 | ENm008 | 16 | 43011 | 47670 | 1 |
| C20orf44 | ENr333 | 20 | 33353784 | 33463359 | -1 |
| C21orf119 | ENm005 | 21 | 32687311 | 32688141 | 1 |
| C21orf120 | ENm005 | 21 | 33084856 | 33085687 | -1 |
| C21orf13 | ENr133 | 21 | 39699641 | 39739602 | -1 |
| C21orf54 | ENm005 | 21 | 33459647 | 33464825 | -1 |
| C21orf59 | ENm005 | 21 | 32886261 | 32907048 | -1 |
| C21orf66 | ENm005 | 21 | 33028082 | 33066041 | -1 |
| C21orf87 | ENr133 | 21 | 39607758 | 39608757 | -1 |
| C22orf30 | ENm004 | 22 | 30396797 | 30470681 | -1 |
| C6orf148 | ENr223 | 6 | 73975315 | 74076660 | -1 |
| C6orf150 | ENr223 | 6 | 74179960 | 74218721 | -1 |
| CACNG6 | ENm007 | 19 | 59187355 | 59207736 | 1 |
| CACNG7 | ENm007 | 19 | 59104402 | 59139008 | 1 |
| CCDC93 | ENr121 | 2 | 118389285 | 118487940 | -1 |
| CDC42BPG | ENr332 | 11 | 64347436 | 64368618 | -1 |
| CDH2 | ENr213 | 18 | 23784929 | 24011409 | -1 |
| CLDN12 | ENm013 | 7 | 89657687 | 89787368 | 1 |
| CPNE1 | ENr333 | 20 | 33677368 | 33725881 | -1 |
| CRYZL1 | ENm005 | 21 | 33883518 | 33938103 | -1 |
| CTD-2183H9.4 | ENm006 | X | 153700865 | 153711083 | -1 |
| CTTNBP2 | ENm001 | 7 | 116944657 | 117108145 | -1 |
| CXorf12 | ENm006 | X | 152758626 | 152769494 | 1 |
| DECR2 | ENm008 | 16 | 391828 | 402489 | 1 |
| DEPDC5 | ENm004 | 22 | 30474499 | 30627556 | 1 |
| DNASE1L1 | ENm006 | X | 153150947 | 153161297 | -1 |
| DOLPP1 | ENr232 | 9 | 128922934 | 128932272 | 1 |
| DONSON | ENm005 | 21 | 33869654 | 34206506 | -1 |
| DRG1 | ENm004 | 22 | 30131553 | 30154993 | 1 |
| DSCR2 | ENr133 | 21 | 39468566 | 39477648 | -1 |
| EIF4ENIF1 | ENm004 | 22 | 30159904 | 30216649 | -1 |
| EMD | ENm006 | X | 153128405 | 153130731 | 1 |
| ENPP1 | ENr222 | 6 | 132221502 | 132257989 | 1 |
| F8A1 | ENm006 | X | 153678329 | 153680041 | 1 |
| FAM3A | ENm006 | X | 153298195 | 153308271 | -1 |
| FBXO7 | ENm004 | 22 | 31195218 | 31219371 | 1 |
| FLNA | ENm006 | X | 153097742 | 153123842 | -1 |
| FRS3 | ENr334 | 6 | 41845893 | 41856183 | -1 |
| FSCN3 | ENm014 | 7 | 126825415 | 126835804 | 1 |
| FUNDC2 | ENm006 | X | 153817960 | 153852283 | 1 |
| FZD1 | ENm013 | 7 | 90538331 | 90542764 | 1 |
| G6PD | ENm006 | X | 153323311 | 153339492 | -1 |
| GART | ENm005 | 21 | 33798109 | 33837668 | -1 |
| GCC1 | ENm014 | 7 | 126814624 | 126827617 | -1 |
| GMPPA | ENr331 | 2 | 220189095 | 220197216 | 1 |
| GTPBP10 | ENm013 | 7 | 89620631 | 89665421 | 1 |
| HBA1 | ENm008 | 16 | 166680 | 167522 | 1 |
| HBA2 | ENm008 | 16 | 162847 | 163710 | 1 |
| HBD | ENm009 | 11 | 5210485 | 5213177 | -1 |
| HBE1 | ENm009 | 11 | 5246159 | 5483424 | -1 |
| HBG1 | ENm009 | 11 | 5225890 | 5227699 | -1 |
| HBG2 | ENm009 | 11 | 5230997 | 5623596 | -1 |
| HCFC1 | ENm006 | X | 152733852 | 152758106 | -1 |
| HOXA5 | ENm010 | 7 | 26953912 | 26956704 | -1 |
| HOXA6 | ENm010 | 7 | 26958256 | 26965458 | -1 |
| HS3ST4 | ENr211 | 16 | 25805569 | 26056511 | 1 |
| HYPK | ENr233 | 15 | 41875633 | 41882534 | 1 |
| IER5L | ENr232 | 9 | 129017386 | 129020096 | -1 |
| IFNAR1 | ENm005 | 21 | 33618653 | 33654039 | 1 |
| IFNAR2 | ENm005 | 21 | 33524077 | 33559840 | 1 |
| IFNGR2 | ENm005 | 21 | 33679170 | 33773526 | 1 |
| IGF2AS | ENm011 | 11 | 2118308 | 2126471 | 1 |
| IKBKG | ENm006 | X | 153333119 | 153359509 | 1 |
| IL10RB | ENm005 | 21 | 33542891 | 33591410 | 1 |
| IL5 | ENm002 | 5 | 131905036 | 131920430 | -1 |
| INS | ENm011 | 11 | 2137585 | 2139148 | -1 |
| IRF1 | ENm002 | 5 | 131845201 | 131854390 | -1 |
| ITGB4BP | ENr333 | 20 | 33330129 | 33336203 | -1 |
| ITSN1 | ENm005 | 21 | 33936577 | 34194036 | 1 |
| KATNAL1 | ENr111 | 13 | 29674768 | 29779622 | -1 |
| KIF3A | ENm002 | 5 | 132056268 | 132101230 | -1 |
| LAGE3 | ENm006 | X | 153269813 | 153271301 | -1 |
| LAIR2 | ENm007 | 19 | 59700913 | 59713710 | 1 |
| LEAP2 | ENm002 | 5 | 132235914 | 132238638 | 1 |
| LENG8 | ENm007 | 19 | 59651878 | 59665030 | 1 |
| LENG9 | ENm007 | 19 | 59664789 | 59666707 | -1 |
| LILRA1 | ENm007 | 19 | 59796860 | 59805368 | 1 |
| LILRA2 | ENm007 | 19 | 59776200 | 59790840 | 1 |
| LILRA3 | ENm007 | 19 | 59491667 | 59501765 | -1 |
| LILRA4 | ENm007 | 19 | 59536269 | 59542234 | -1 |
| LILRA5 | ENm007 | 19 | 59510166 | 59516222 | -1 |
| LILRA6 | ENm007 | 19 | 59432281 | 59438943 | -1 |
| LILRB2 | ENm007 | 19 | 59469488 | 59476852 | -1 |
| LILRB4 | ENm007 | 19 | 59847153 | 59873623 | 1 |
| LILRB5 | ENm007 | 19 | 59446076 | 59452977 | -1 |
| LL22NC03-104C7.1 | ENm004 | 22 | 31303841 | 31304745 | 1 |
| LSP1 | ENm011 | 11 | 1830777 | 1870074 | 1 |
| LUC7L | ENm008 | 16 | 178970 | 219464 | -1 |
| MAP3K1 | ENr221 | 5 | 56147159 | 56227737 | 1 |
| MAP4K2 | ENr332 | 11 | 64313186 | 64327290 | -1 |
| MDFIC | ENm012 | 7 | 114156161 | 114253208 | 1 |
| MEN1 | ENr332 | 11 | 64327565 | 64335343 | -1 |
| MFAP1 | ENr233 | 15 | 41883983 | 41904293 | -1 |
| MMP24 | ENr333 | 20 | 33305670 | 33328216 | 1 |
| MPG | ENm008 | 16 | 67007 | 75853 | 1 |
| MPP1 | ENm006 | X | 153570664 | 153612987 | -1 |
| MRPL28 | ENm008 | 16 | 356929 | 360570 | -1 |
| MTCP1 | ENm006 | X | 153853602 | 153939917 | -1 |
| MYADM | ENm007 | 19 | 59061290 | 59071504 | 1 |
| NR2E1 | ENr323 | 6 | 108593956 | 108616707 | 1 |
| OPN1LW | ENm006 | X | 152930593 | 152945355 | 1 |
| OPN1MW | ENm006 | X | 152969002 | 152982481 | 1 |
| OR51A2 | ENm009 | 11 | 4932579 | 4933520 | -1 |
| OR51A4 | ENm009 | 11 | 4923966 | 4924907 | -1 |
| OR51B5 | ENm009 | 11 | 5320393 | 5321331 | -1 |
| OR51B6 | ENm009 | 11 | 5329315 | 5330253 | 1 |
| OR51B8P | ENm009 | 11 | 5308399 | 5309167 | -1 |
| OR51G1 | ENm009 | 11 | 4901181 | 4902146 | -1 |
| OR51G2 | ENm009 | 11 | 4892526 | 4893470 | -1 |
| OR51T1 | ENm009 | 11 | 4859707 | 4860690 | 1 |
| OR52A4 | ENm009 | 11 | 5098471 | 5102320 | -1 |
| OR52H1 | ENm009 | 11 | 5522368 | 5523330 | -1 |
| OR52Y1P | ENm009 | 11 | 4771585 | 4773091 | -1 |
| OR56B1 | ENm009 | 11 | 5686015 | 5715298 | 1 |
| OSCAR | ENm007 | 19 | 59289746 | 59297813 | -1 |
| OSTM1 | ENr323 | 6 | 108469307 | 108502639 | -1 |
| P4HA2 | ENm002 | 5 | 131555431 | 131658908 | -1 |
| PDIA3 | ENr233 | 15 | 41825883 | 41852770 | 1 |
| PFTK1 | ENm013 | 7 | 89740390 | 90484557 | 1 |
| PISD | ENm004 | 22 | 30339032 | 30382973 | -1 |
| POLR3K | ENm008 | 16 | 36408 | 43629 | -1 |
| PPP2R4 | ENr232 | 9 | 128952784 | 128990780 | 1 |
| PRKCG | ENm007 | 19 | 59074257 | 59102719 | 1 |
| PRPF31 | ENm007 | 19 | 59310650 | 59326956 | 1 |
| PSMB4 | ENr231 | 1 | 148185084 | 148187494 | 1 |
| PSMD4 | ENr231 | 1 | 148040253 | 148053029 | 1 |
| PYGM | ENr332 | 11 | 64270438 | 64284346 | -1 |
| RAB11FIP3 | ENm008 | 16 | 415621 | 495629 | 1 |
| RAD50 | ENm002 | 5 | 131919611 | 132007652 | 1 |
| RASGRP2 | ENr332 | 11 | 64250960 | 64269505 | -1 |
| RBM12 | ENr333 | 20 | 33700262 | 33716253 | -1 |
| RFX5 | ENr231 | 1 | 148126190 | 148132907 | -1 |
| RGS11 | ENm008 | 16 | 258302 | 265982 | -1 |
| RP1-111B22.2 | ENr323 | 6 | 108432255 | 108433466 | -1 |
| RP11-120K24.2 | ENr132 | 13 | 112669825 | 112671140 | -1 |
| RP11-126K1.2 | ENr231 | 1 | 148065574 | 148067479 | -1 |
| RP11-247A12.2 | ENr232 | 9 | 129018605 | 129052382 | 1 |
| RP11-247A12.6 | ENr232 | 9 | 128936628 | 128952564 | -1 |
| RP11-247I13.3 | ENm004 | 22 | 30280024 | 30280480 | 1 |
| RP11-247I13.6 | ENm004 | 22 | 30258893 | 30259130 | -1 |
| RP1-128O3.6 | ENr323 | 6 | 108745977 | 108746302 | 1 |
| RP11-344B5.2 | ENr232 | 9 | 129124292 | 129127562 | 1 |
| RP11-374F3.2 | ENr111 | 13 | 29768285 | 29768873 | 1 |
| RP11-374F3.4 | ENr111 | 13 | 29812408 | 29849283 | -1 |
| RP11-398K22.12 | ENr223 | 6 | 74029660 | 74067846 | 1 |
| RP11-398K22.13 | ENr223 | 6 | 74056848 | 74057707 | 1 |
| RP11-398K22.3 | ENr223 | 6 | 74139553 | 74139708 | -1 |
| RP11-398K22.4 | ENr223 | 6 | 74129122 | 74130616 | 1 |
| RP11-398K22.9 | ENr223 | 6 | 74058407 | 74059722 | 1 |
| RP11-490N5.1 | ENr111 | 13 | 29626204 | 29626724 | 1 |
| RP1-149A16.15 | ENm004 | 22 | 31097224 | 31097677 | 1 |
| RP11-65J3.6 | ENr232 | 9 | 129221975 | 129222304 | -1 |
| RP11-69I8.2 | ENr222 | 6 | 132264797 | 132283399 | 1 |
| RP11-74C1.2 | ENr231 | 1 | 148342996 | 148343490 | 1 |
| RP1-18D14.7 | SCL | 1 | 47403490 | 47408443 | 1 |
| RP1-191J18.65 | ENr323 | 6 | 108412987 | 108413588 | -1 |
| RP11-98F14.4 | ENr132 | 13 | 112832732 | 112834370 | 1 |
| RP1-248E1.2 | ENr222 | 6 | 132634815 | 132636165 | 1 |
| RP1-90G24.10 | ENm004 | 22 | 30925657 | 30990208 | 1 |
| RP1-90G24.5 | ENm004 | 22 | 30989923 | 30993652 | 1 |
| RP1-90G24.8 | ENm004 | 22 | 30879996 | 30880934 | -1 |
| RP3-429G5.3 | ENr323 | 6 | 108551413 | 108587290 | 1 |
| RP3-477O4.15 | ENr333 | 20 | 33564811 | 33568775 | 1 |
| RP3-477O4.5 | ENr333 | 20 | 33572095 | 33574416 | -1 |
| RP3-523C21.2 | ENr222 | 6 | 132447918 | 132462113 | 1 |
| RP5-931E15.4 | ENr324 | X | 122644395 | 122644837 | 1 |
| RPL10 | ENm006 | X | 153147247 | 153151528 | 1 |
| RPL36P4 | ENr333 | 20 | 33595590 | 33595907 | -1 |
| RPS17P4 | ENm004 | 22 | 30760032 | 30760438 | 1 |
| RPS5L | ENm005 | 21 | 33775758 | 33776373 | -1 |
| RPS9 | ENm007 | 19 | 59396423 | 59444675 | 1 |
| SELENBP1 | ENr231 | 1 | 148149852 | 148158283 | -1 |
| SERPINB10 | ENr122 | 18 | 59715389 | 59754326 | 1 |
| SERPINB2 | ENr122 | 18 | 59689907 | 59722105 | 1 |
| SERPINB8 | ENr122 | 18 | 59788140 | 59823259 | 1 |
| SETD3 | ENr322 | 14 | 98933837 | 98950025 | -1 |
| SF1 | ENr332 | 11 | 64288655 | 64302835 | -1 |
| SFI1 | ENm004 | 22 | 30209229 | 30339092 | 1 |
| SH3BGR | ENr133 | 21 | 39739652 | 39739895 | 1 |
| SH3GLB2 | ENr232 | 9 | 128848870 | 128870137 | -1 |
| SHROOM1 | ENm002 | 5 | 132185733 | 132194490 | -1 |
| SIL | SCL | 1 | 47427870 | 47491840 | -1 |
| SLC10A3 | ENm006 | X | 153279350 | 153282707 | -1 |
| SLC22A4 | ENm002 | 5 | 131658036 | 131707799 | 1 |
| SLC22A5 | ENm002 | 5 | 131733344 | 131759206 | 1 |
| SLC4A3 | ENr331 | 2 | 220317793 | 220332208 | 1 |
| SLC5A1 | ENm004 | 22 | 30763574 | 30833571 | 1 |
| SLC5A4 | ENm004 | 22 | 30939020 | 30975883 | -1 |
| SNX3 | ENr323 | 6 | 108639120 | 108689158 | -1 |
| SON | ENm005 | 21 | 33836795 | 33871658 | 1 |
| ST7 | ENm001 | 7 | 116187333 | 116464109 | 1 |
| STAG2 | ENr324 | X | 122819598 | 122962042 | 1 |
| SYN3 | ENm004 | 22 | 31233094 | 31778913 | -1 |
| SYNJ1 | ENm005 | 21 | 32922945 | 33022184 | -1 |
| SYT8 | ENm011 | 11 | 1806114 | 1815328 | 1 |
| TAZ | ENm006 | X | 153160702 | 153170913 | 1 |
| TES | ENm001 | 7 | 115444499 | 115492789 | 1 |
| TFEB | ENr334 | 6 | 41759695 | 41811976 | -1 |
| TIMP3 | ENm004 | 22 | 31522242 | 31583585 | 1 |
| TMC4 | ENm007 | 19 | 59355659 | 59368757 | -1 |
| TMEM15 | ENr232 | 9 | 128787364 | 128789453 | -1 |
| TMEM8 | ENm008 | 16 | 360775 | 377115 | -1 |
| TNNT3 | ENm011 | 11 | 1897369 | 1916513 | 1 |
| TRIM22 | ENm009 | 11 | 5667496 | 5692850 | 1 |
| TRIM6 | ENm009 | 11 | 5573916 | 5590765 | 1 |
| TSEN34 | ENm007 | 19 | 59385602 | 59389334 | 1 |
| TSPAN32 | ENm011 | 11 | 2279804 | 2296007 | 1 |
| TUFT1 | ENr231 | 1 | 148325855 | 148369133 | 1 |
| UBL4 | ENm006 | X | 153275761 | 153278705 | -1 |
| UBQLN3 | ENm009 | 11 | 5485107 | 5487792 | -1 |
| UQCRQ | ENm002 | 5 | 132230152 | 132231623 | 1 |
| WNT2 | ENm001 | 7 | 116510637 | 116557295 | -1 |
| WRB | ENr133 | 21 | 39674041 | 39691686 | 1 |
| XX-FW88778H2.2 | ENm006 | X | 153121959 | 153123854 | -1 |
| YWHAH | ENm004 | 22 | 30665002 | 30678145 | 1 |
| Z69666.2 | ENm008 | 16 | 115737 | 117219 | 1 |
| Z84812.1 | ENm008 | 16 | 283 | 4091 | 1 |
| Z84812.2 | ENm008 | 16 | 4044 | 9453 | -1 |
| ZNF259 | ENm003 | 11 | 116153647 | 116163977 | -1 |
| ZNF687 | ENr231 | 1 | 148067168 | 148077455 | 1 |
| ZNF800 | ENm014 | 7 | 126580796 | 126665930 | -1 |

Supplementary Table S.7

| **Gene ID** | **Region** | **Chr** | **Start** | **End** | **Strand** |
| --- | --- | --- | --- | --- | --- |
| AC000364.1 | ENm014 | 7 | 126314699 | 126324924 | -1 |
| AC002064.2 | ENm013 | 7 | 89519151 | 89585029 | 1 |
| AC004009.2 | ENm010 | 7 | 27225024 | 27225751 | 1 |
| AC004080.13 | ENm010 | 7 | 26941829 | 26965421 | -1 |
| AC004080.18 | ENm010 | 7 | 27052103 | 27054088 | -1 |
| AC005538.2 | ENr131 | 2 | 234556084 | 234559056 | -1 |
| AC005538.3 | ENr131 | 2 | 234556143 | 234558006 | -1 |
| AC005538.5 | ENr131 | 2 | 234636791 | 234646080 | -1 |
| AC006159.3 | ENm001 | 7 | 115804966 | 115848689 | -1 |
| AC006293.1 | ENm007 | 19 | 59912199 | 59916547 | 1 |
| AC006293.3 | ENm007 | 19 | 59913804 | 59916768 | 1 |
| AC006985.5 | ENr131 | 2 | 234466371 | 234524070 | 1 |
| AC008984.2 | ENm007 | 19 | 59511995 | 59540252 | -1 |
| AC008984.4 | ENm007 | 19 | 59523289 | 59524297 | -1 |
| AC009158.1 | ENr211 | 16 | 26236969 | 26253251 | 1 |
| AC009303.1 | ENr121 | 2 | 118469417 | 118470417 | 1 |
| AC009404.2 | ENr121 | 2 | 118307744 | 118315465 | 1 |
| AC009892.8 | ENm007 | 19 | 59851206 | 59852115 | 1 |
| AC009955.5 | ENr331 | 2 | 220240957 | 220261749 | -1 |
| AC011330.5 | ENr233 | 15 | 41743145 | 41763830 | -1 |
| AC011330.8 | ENr233 | 15 | 41778979 | 41797675 | -1 |
| AC012314.6 | ENm007 | 19 | 59308899 | 59309407 | -1 |
| AC023590.1 | ENr321 | 8 | 119363663 | 119377115 | 1 |
| AC051649.15 | ENm011 | 11 | 1866952 | 1868661 | -1 |
| AC053503.11 | ENr331 | 2 | 220172301 | 220207105 | -1 |
| AC073472.2 | ENm010 | 7 | 26795628 | 26796227 | -1 |
| AC080091.1 | ENr112 | 2 | 51648932 | 51650273 | 1 |
| AC087380.14 | ENm009 | 11 | 5496248 | 5502762 | -1 |
| AC092661.1 | ENr113 | 4 | 118707156 | 118969784 | 1 |
| AC106873.2 | ENm001 | 7 | 116206171 | 116206917 | -1 |
| AC113331.9 | ENm009 | 11 | 5083869 | 5085337 | -1 |
| AC113617.1 | ENr113 | 4 | 118639385 | 118651612 | 1 |
| ACSL6 | ENm002 | 5 | 131285415 | 131375770 | -1 |
| AF064858.7 | ENr133 | 21 | 39268226 | 39271571 | -1 |
| AF277315.12 | ENm006 | X | 153450769 | 153451661 | -1 |
| AP000279.69 | ENm005 | 21 | 33022298 | 33037305 | 1 |
| AP000282.3 | ENm005 | 21 | 33253067 | 33254745 | 1 |
| AP000290.7 | ENm005 | 21 | 33450809 | 33453287 | 1 |
| AP001092.5 | ENr332 | 11 | 64175344 | 64180360 | 1 |
| AP003774.4 | ENr332 | 11 | 63973123 | 63975703 | 1 |
| AP005273.1 | ENr332 | 11 | 64024902 | 64029435 | 1 |
| AP006216.5 | ENm003 | 11 | 116189131 | 116189930 | -1 |
| ASZ1 | ENm001 | 7 | 116597228 | 116662129 | -1 |
| ATP6AP1 | ENm006 | X | 153177832 | 153183640 | 1 |
| BIRC4 | ENr324 | X | 122719110 | 122773365 | 1 |
| BRWD1 | ENr133 | 21 | 39477973 | 39615356 | -1 |
| C20orf173 | ENr333 | 20 | 33578214 | 33580892 | -1 |
| C21orf55 | ENm005 | 21 | 33779708 | 33785898 | -1 |
| C21orf77 | ENm005 | 21 | 32866420 | 32870063 | -1 |
| C22orf24 | ENm004 | 22 | 30654062 | 30666059 | -1 |
| C22orf28 | ENm004 | 22 | 31108124 | 31132797 | -1 |
| CATSPER2 | ENr233 | 15 | 41707994 | 41747609 | -1 |
| CAV2 | ENm001 | 7 | 115522079 | 115742547 | 1 |
| CGN | ENr231 | 1 | 148296060 | 148324242 | 1 |
| CKMT1A | ENr233 | 15 | 41772377 | 41778713 | 1 |
| CKMT1B | ENr233 | 15 | 41672545 | 41678897 | 1 |
| CRAT | ENr232 | 9 | 128936644 | 128953023 | -1 |
| CTA-342B11.1 | ENm004 | 22 | 30683899 | 30690942 | 1 |
| CYP4A22 | SCL | 1 | 47315128 | 47327434 | 1 |
| DDX18 | ENr121 | 2 | 118288457 | 118306186 | 1 |
| EEF1A1 | ENr223 | 6 | 74282195 | 74288345 | -1 |
| EVX1 | ENm010 | 7 | 27055525 | 27060692 | 1 |
| FAM73B | ENr232 | 9 | 128878455 | 128913916 | 1 |
| FER1L4 | ENr333 | 20 | 33609922 | 33658899 | -1 |
| FRMD5 | ENr233 | 15 | 41950255 | 42003800 | -1 |
| GAB3 | ENm006 | X | 153467234 | 153543563 | -1 |
| GRM8 | ENm014 | 7 | 125672608 | 126487300 | -1 |
| H19 | ENm011 | 11 | 1972983 | 1979277 | -1 |
| H2AFB1 | ENm006 | X | 153676952 | 153677538 | 1 |
| HBQ1 | ENm008 | 16 | 170453 | 171181 | 1 |
| HOXA1 | ENm010 | 7 | 26905853 | 26908834 | -1 |
| HOXA11 | ENm010 | 7 | 26994017 | 26998083 | -1 |
| HOXA11S | ENm010 | 7 | 26998268 | 27002153 | 1 |
| HOXA13 | ENm010 | 7 | 27008263 | 27012966 | -1 |
| HOXA2 | ENm010 | 7 | 26913217 | 26915546 | -1 |
| HOXA3 | ENm010 | 7 | 26919044 | 26953068 | -1 |
| HOXA4 | ENm010 | 7 | 26941367 | 26943659 | -1 |
| IGF2 | ENm011 | 11 | 2106919 | 2138797 | -1 |
| IL3 | ENm002 | 5 | 131424122 | 131426797 | 1 |
| IL4 | ENm002 | 5 | 132037578 | 132046268 | 1 |
| INHA | ENr331 | 2 | 220259390 | 220265941 | 1 |
| KIR3DX1 | ENm007 | 19 | 59735790 | 59748866 | 1 |
| L1CAM | ENm006 | X | 152647817 | 152695525 | -1 |
| LILRB3 | ENm007 | 19 | 59411960 | 59419191 | -1 |
| MCF2L | ENr132 | 13 | 112604511 | 112802055 | 1 |
| MDFI | ENr334 | 6 | 41712599 | 41729963 | 1 |
| NCR2 | ENr334 | 6 | 41411372 | 41426604 | 1 |
| OLIG1 | ENm005 | 21 | 33364321 | 33366597 | 1 |
| OLIG2 | ENm005 | 21 | 33320024 | 33323375 | 1 |
| OR51A10P | ENm009 | 11 | 5446293 | 5447229 | -1 |
| OR51A5P | ENm009 | 11 | 4950750 | 4951691 | -1 |
| OR51A6P | ENm009 | 11 | 4867400 | 4868403 | -1 |
| OR51B2 | ENm009 | 11 | 5301166 | 5302104 | -1 |
| OR51B4 | ENm009 | 11 | 5278821 | 5279803 | -1 |
| OR51F1 | ENm009 | 11 | 4746786 | 4747745 | -1 |
| OR51I1 | ENm009 | 11 | 5418377 | 5419321 | -1 |
| OR51I2 | ENm009 | 11 | 5431296 | 5432234 | 1 |
| OR51K1P | ENm009 | 11 | 5408460 | 5409409 | -1 |
| OR51N1P | ENm009 | 11 | 4764561 | 4765512 | 1 |
| OR51P1P | ENm009 | 11 | 4992945 | 4993886 | 1 |
| OR51Q1 | ENm009 | 11 | 5400008 | 5400961 | 1 |
| OR52A1 | ENm009 | 11 | 5128816 | 5164189 | -1 |
| OR52B5P | ENm009 | 11 | 5538774 | 5539724 | 1 |
| OR52B6 | ENm009 | 11 | 5558684 | 5559691 | 1 |
| OR52D1 | ENm009 | 11 | 5466514 | 5467470 | 1 |
| OR52E2 | ENm009 | 11 | 5036457 | 5037434 | -1 |
| OR52E3P | ENm009 | 11 | 5070483 | 5071418 | 1 |
| OR52H2P | ENm009 | 11 | 5529469 | 5530370 | -1 |
| OR52J2P | ENm009 | 11 | 5014820 | 5015758 | 1 |
| OR52J3 | ENm009 | 11 | 5024333 | 5025268 | 1 |
| OR52P1P | ENm009 | 11 | 5704309 | 5705271 | 1 |
| PCDH15 | ENr114 | 10 | 55232538 | 55643815 | -1 |
| PDZK1IP1 | SCL | 1 | 47361286 | 47368737 | -1 |
| POGZ | ENr231 | 1 | 148188274 | 148245015 | -1 |
| RP11-247I13.8 | ENm004 | 22 | 30182205 | 30184745 | 1 |
| RP11-257K9.7 | ENr223 | 6 | 73989989 | 73992215 | -1 |
| RP1-128O3.5 | ENr323 | 6 | 108680112 | 108680298 | 1 |
| RP11-328M4.3 | ENr334 | 6 | 41578161 | 41595569 | 1 |
| RP11-344B5.4 | ENr232 | 9 | 129100188 | 129101680 | 1 |
| RP11-380M3.3 | ENr223 | 6 | 73821542 | 73822302 | 1 |
| RP11-398K22.7 | ENr223 | 6 | 74119507 | 74120740 | -1 |
| RP1-149A16.11 | ENm004 | 22 | 31033633 | 31036187 | 1 |
| RP1-149A16.17 | ENm004 | 22 | 31104328 | 31106173 | 1 |
| RP1-149A16.3 | ENm004 | 22 | 31097206 | 31104885 | 1 |
| RP1-149M18.4 | ENr334 | 6 | 41481536 | 41482278 | 1 |
| RP11-629E24.1 | ENr111 | 13 | 29575316 | 29581013 | -1 |
| RP1-180M12.1 | ENm004 | 22 | 30610885 | 30611975 | -1 |
| RP1-302D9.1 | ENm004 | 22 | 31825924 | 31826600 | -1 |
| SERPINB11 | ENr122 | 18 | 59465794 | 59542104 | 1 |
| SERPINB13 | ENr122 | 18 | 59412570 | 59422854 | 1 |
| SLC22A11 | ENr332 | 11 | 64079675 | 64096924 | 1 |
| SLC22A12 | ENr332 | 11 | 64114690 | 64126397 | 1 |
| SPAG4 | ENr333 | 20 | 33667229 | 33672386 | 1 |
| SPP2 | ENr131 | 2 | 234741324 | 234767779 | 1 |
| ST7OT1 | ENm001 | 7 | 116186452 | 116188340 | -1 |
| ST7OT2 | ENm001 | 7 | 116306078 | 116380486 | -1 |
| STRC | ENr233 | 15 | 41678889 | 41698291 | -1 |
| TH | ENm011 | 11 | 2141736 | 2149684 | -1 |
| TKTL1 | ENm006 | X | 153044872 | 153079548 | 1 |
| TRIM34 | ENm009 | 11 | 5597571 | 5622205 | 1 |
| TRIM5 | ENm009 | 11 | 5641364 | 5663201 | -1 |
| TRIM6-TRIM34 | ENm009 | 11 | 5574532 | 5622205 | 1 |
| U52112.12 | ENm006 | X | 152666975 | 152675292 | 1 |
| UGT1A11P | ENr131 | 2 | 234294200 | 234295048 | 1 |
| UGT1A2P | ENr131 | 2 | 234437861 | 234438726 | 1 |
| Z84723.1 | ENm008 | 16 | 12911 | 15124 | 1 |
| Z97634.3 | ENm008 | 16 | 376765 | 377235 | 1 |
| Z97634.5 | ENm008 | 16 | 372099 | 382962 | 1 |
