# Supplementary figures and images for: Complex Exon-Intron Marking by Histone Modifications Is Not Determined Solely by Nucleosome Distribution
Source: PLoS One. 2010 Aug 23;5(8):e12339. doi: 10.1371/journal.pone.0012339 (PMC2925886; doi:10.1371/journal.pone.0012339)

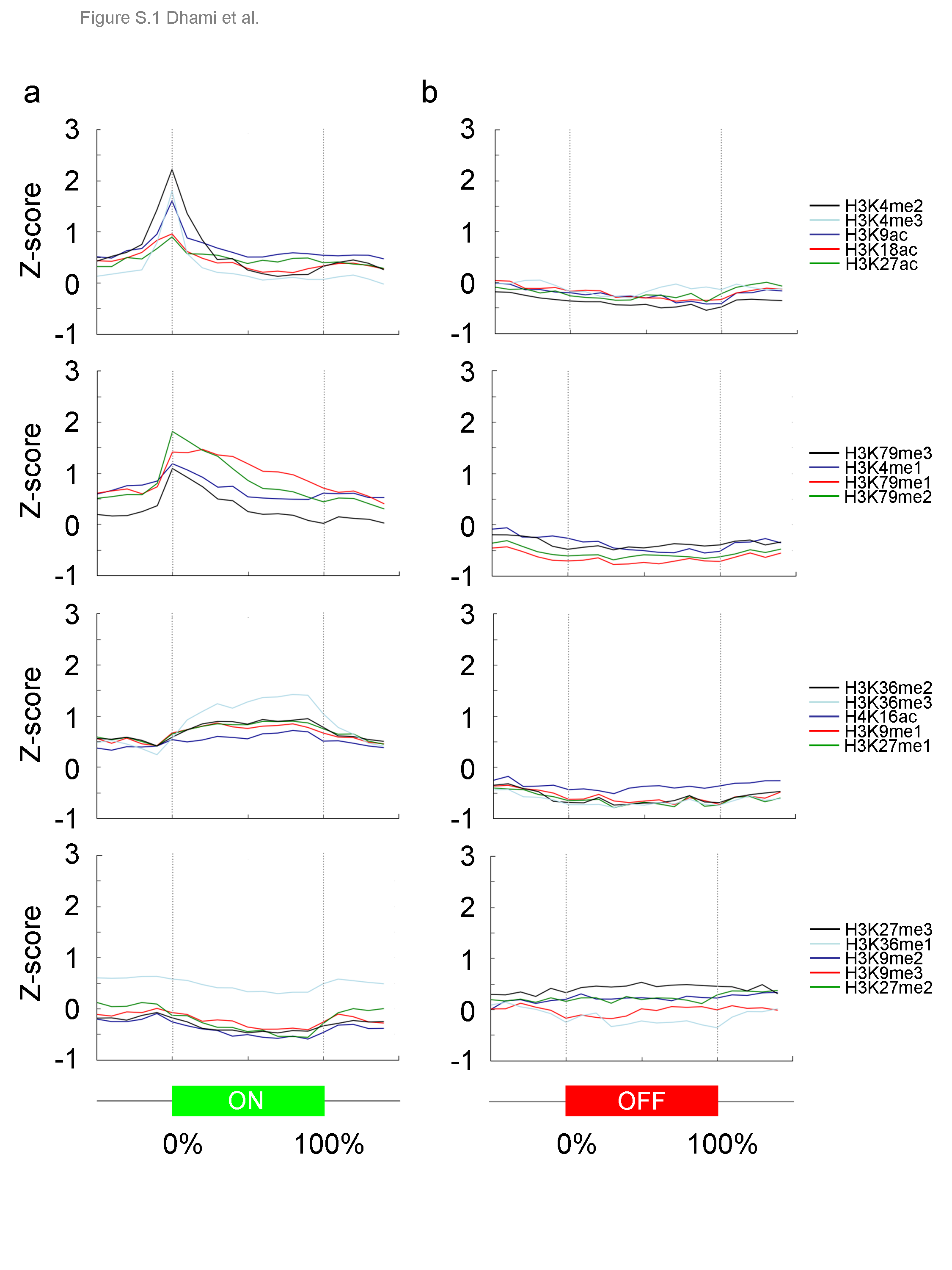

Supplement: Figure S1 — Histone modification patterns for expressed and non-expressed genes across the ENCODE regions in the K562 and U937 cell lines and CD14+ monocytes. a. Consensus gene plots for 19 histone modifications across expressed (ON) genes (n = 366). b. Consensus gene plots for 19 histone modifications across non-expressed (OFF) genes (n = 167). ChIP-chip enrichment levels in both panels are expressed as mean Z-scores. Proportional gene length and flanking regions are shown on the x axis as percentages (%). Color key to modifications depicted in each panel are shown to the right of the figure. Some modifications showed strong association with 5′ ends (i.e., promoters) or with gene bodies of actively transcribed genes (with either a 5′ or 3′ bias). Other modifications showed depletions across gene bodies of expressed genes. Consensus plots for non-expressed genes exhibited the typical hallmarks of H3K27me3 and H3K9me2 enrichments. (1.14 MB TIF) [file pone.0012339.s002.tif]

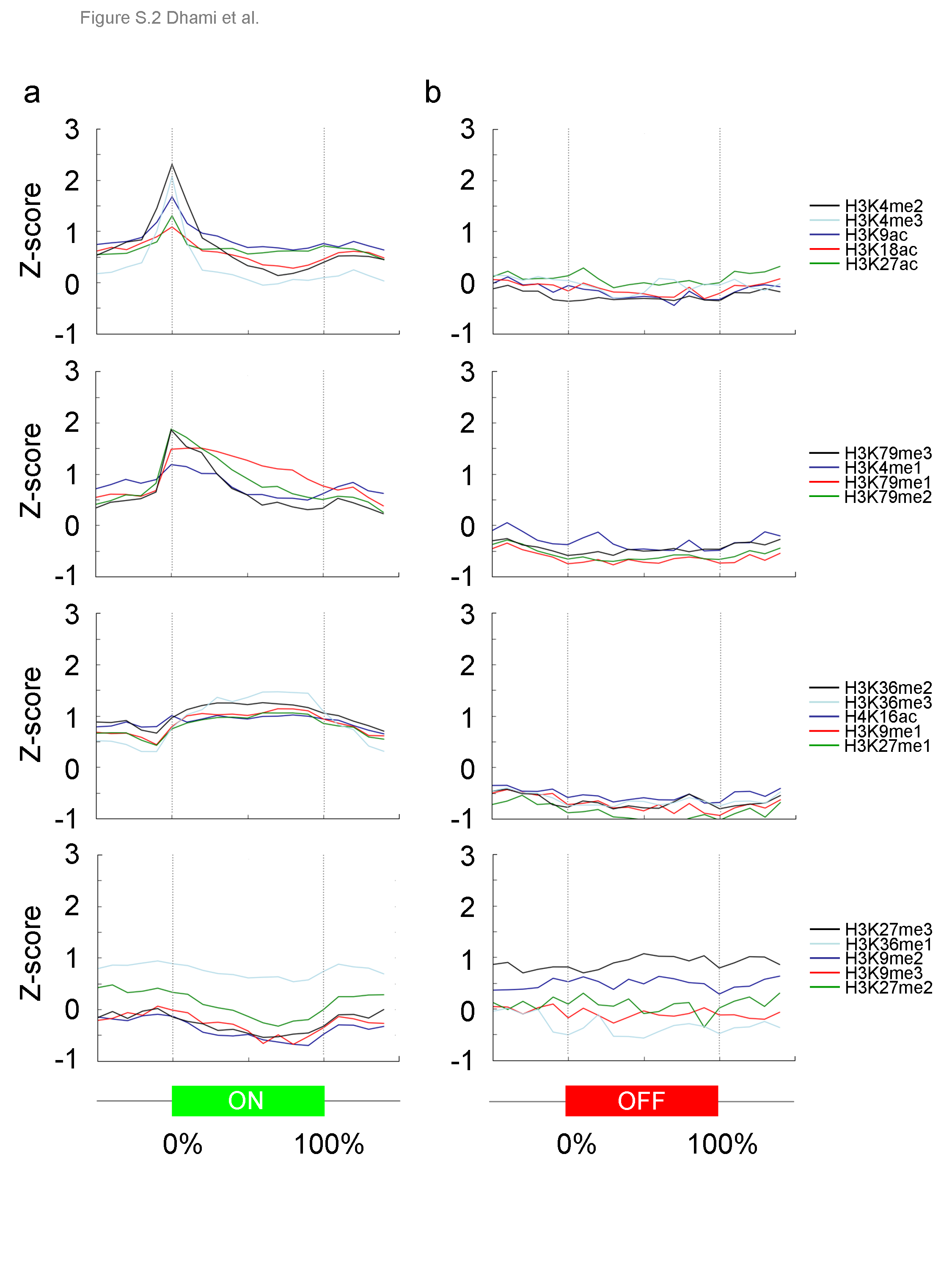

Supplement: Figure S2 — Histone modification patterns for expressed and non-expressed genes across the ENCODE regions in the K562 cell line. a. Consensus gene plots for 19 histone modifications across expressed (ON) genes (n = 111). b. Consensus gene plots for 19 histone modifications across non-expressed (OFF) genes (n = 53). ChIP-chip enrichment levels in both panels are expressed as mean Z-scores. Proportional gene length and flanking regions are shown on the x axis as percentages (%). Color key to modifications depicted in each panel are shown to the right of the figure. Trends were as described in Supplementary Figure S1. (1.16 MB TIF) [file pone.0012339.s003.tif]

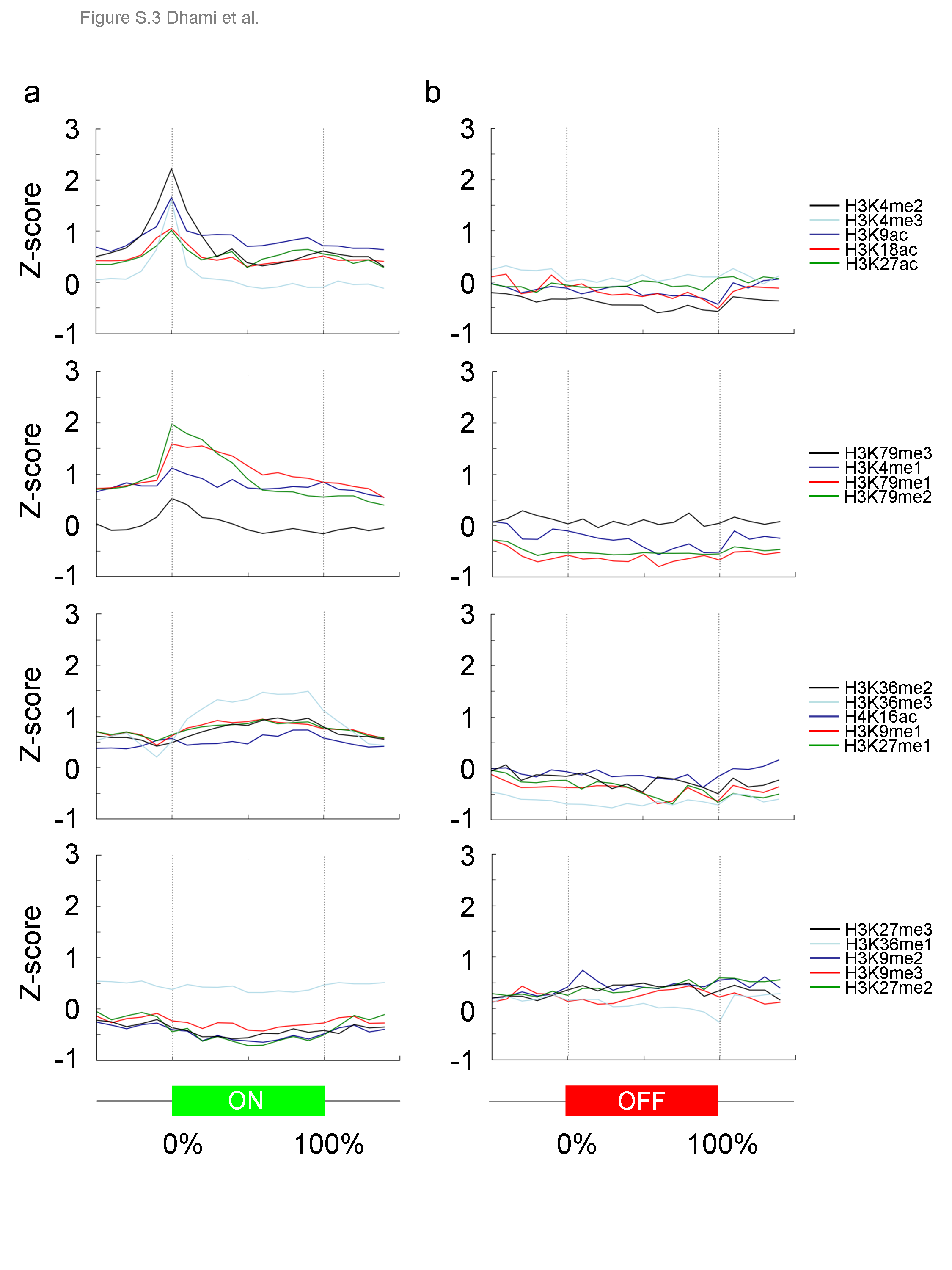

Supplement: Figure S3 — Histone modification patterns for expressed and non-expressed genes across the ENCODE regions in the U937 cell line. a. Consensus gene plots for 19 histone modifications across expressed (ON) genes (n = 134). b. Consensus gene plots for 19 histone modifications across non-expressed (OFF) genes (n = 62). ChIP-chip enrichment levels in both panels are expressed as mean Z-scores. Proportional gene length and flanking regions are shown on the x axis as percentages (%). Color key to modifications depicted in each panel are shown to the right of the figure. Trends were as described in Supplementary Figure S1. (1.15 MB TIF) [file pone.0012339.s004.tif]

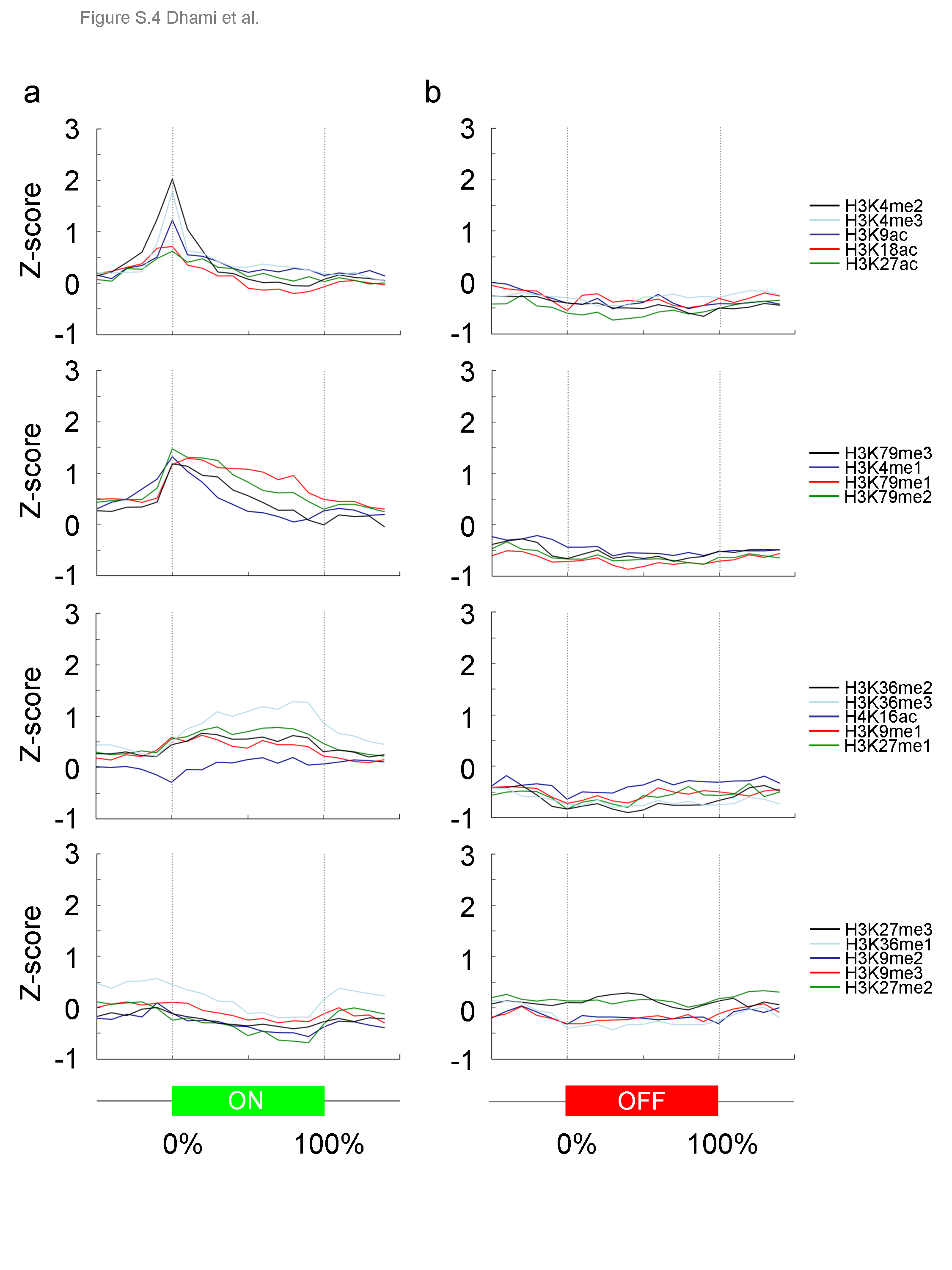

Supplement: Figure S4 — Histone modification patterns for expressed and non-expressed genes across the ENCODE regions in CD14+ monocytes. a. Consensus gene plots for 19 histone modifications across expressed (ON) genes (n = 121). b. Consensus gene plots for 19 histone modifications across non-expressed (OFF) genes (n = 52). ChIP-chip enrichment levels in both panels are expressed as mean Z-scores. Proportional gene length and flanking regions are shown on the x axis as percentages (%). Color key to modifications depicted in each panel are shown to the right of the figure. Trends were as described in Supplementary Figure S1. (1.16 MB TIF) [file pone.0012339.s005.tif]

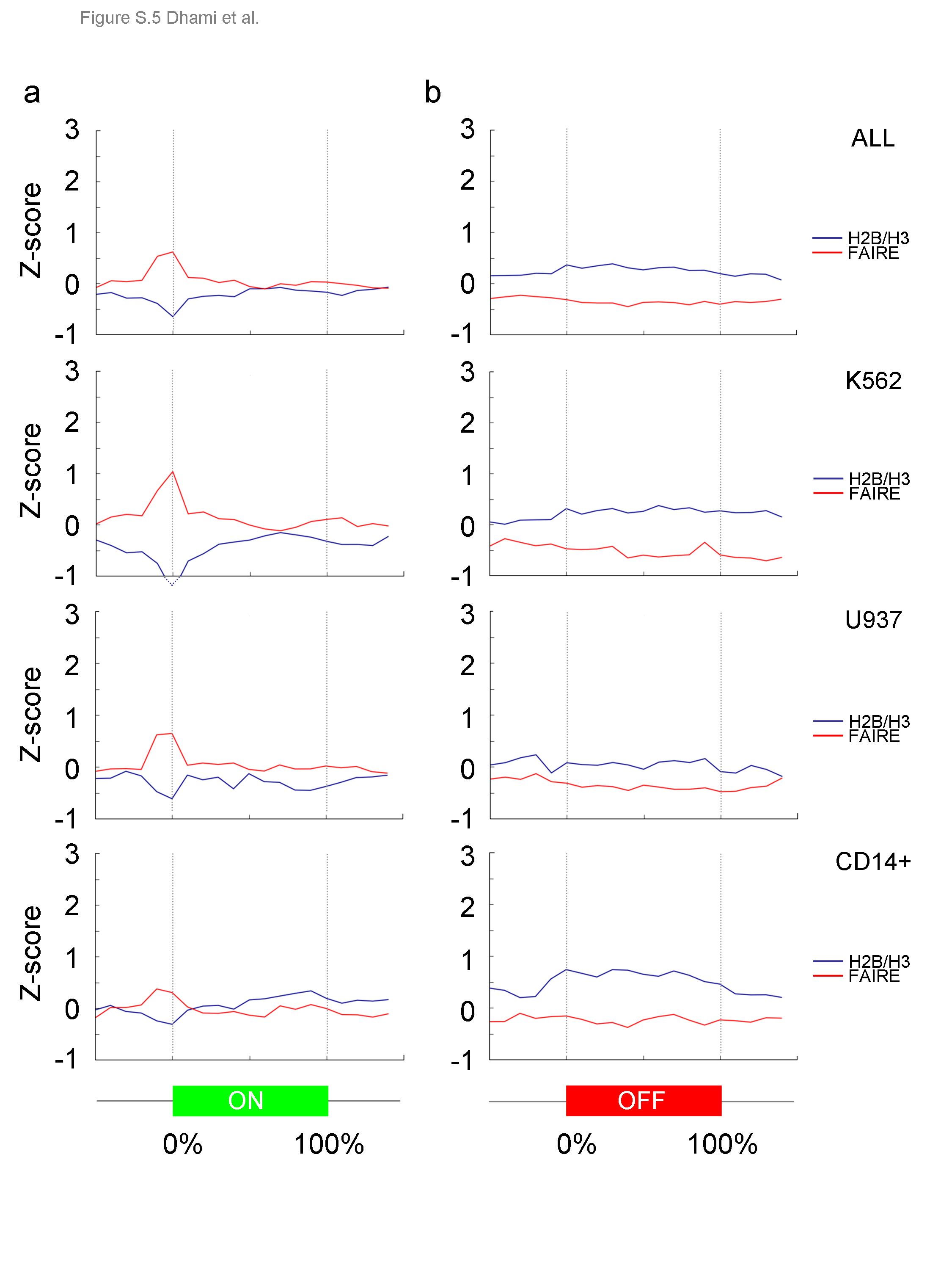

Supplement: Figure S5 — Chromatin accessibility (FAIRE) and histone density patterns (H2B/H3) for expressed and non-expressed genes across the ENCODE regions in the K562 and U937 cell lines and CD14+ monocytes. a. Consensus gene plots across expressed (ON) genes in all three cell types [ALL (n = 366), K562 (n = 111), U937 (n = 134), and CD14+ monocytes (n = 121)]. b. Consensus gene plots across non-expressed (OFF) genes in all three cell types [ALL (n = 167), K562 (n = 53), U937 (n = 62), and CD14+ monocytes (n = 52)]. ChIP-chip enrichment levels in both panels are expressed as mean Z-scores. Proportional gene length and flanking regions are shown on the x axis as percentages (%). Color key to FAIRE and histone density assays in each panel are shown to the right of the figure. (0.88 MB TIF) [file pone.0012339.s006.tif]

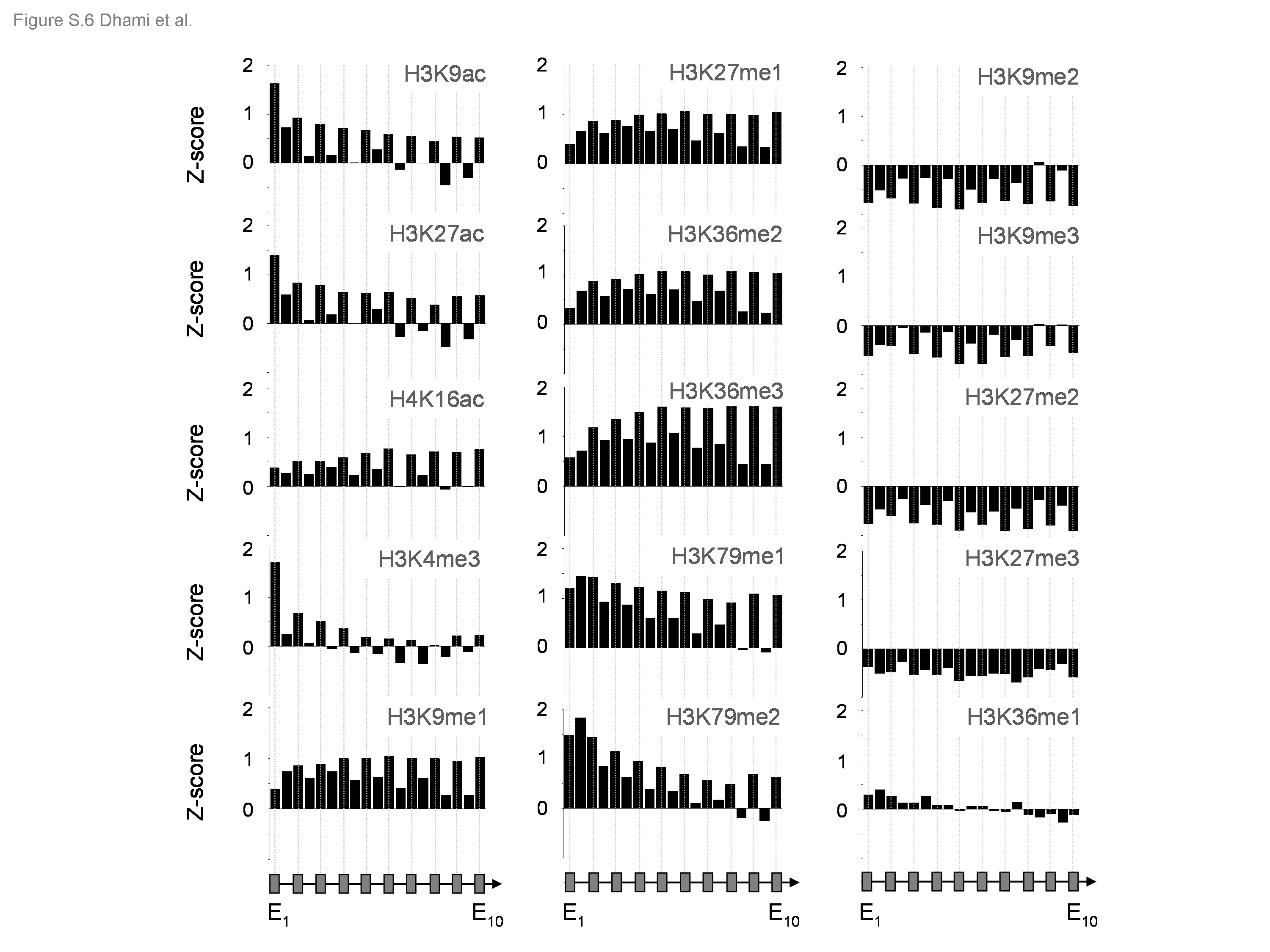

Supplement: Figure S6 — Histone modification patterns track exons and introns across gene bodies without accounting for nucleosome distribution. Histograms show the mean levels of ChIP-chip enrichments (Z-scores) for 15 histone modifications spanning the first ten exons and nine introns of expressed consensus genes (n = 268, exons∶introns = 1466∶551). Data is derived from ENCODE regions in the K562 and U937 cell lines and CD14+ primary monocytes. Datasets are not normalized with the combined histone distribution profiles obtained for H2B and H3 in each cell line. Hypothetical gene structures are shown at the bottom of the figure. Median P-value obtained from bootstrapping for exons and introns across all 19 histone modifications tested in this study was <1.0×10^−15^. Median P-value obtained for pair-wise t-tests between adjacent exon-intron pairs (exon2 → exon10) for the data shown in the figure was 1.13×10^−6^. (0.94 MB TIF) [file pone.0012339.s007.tif]

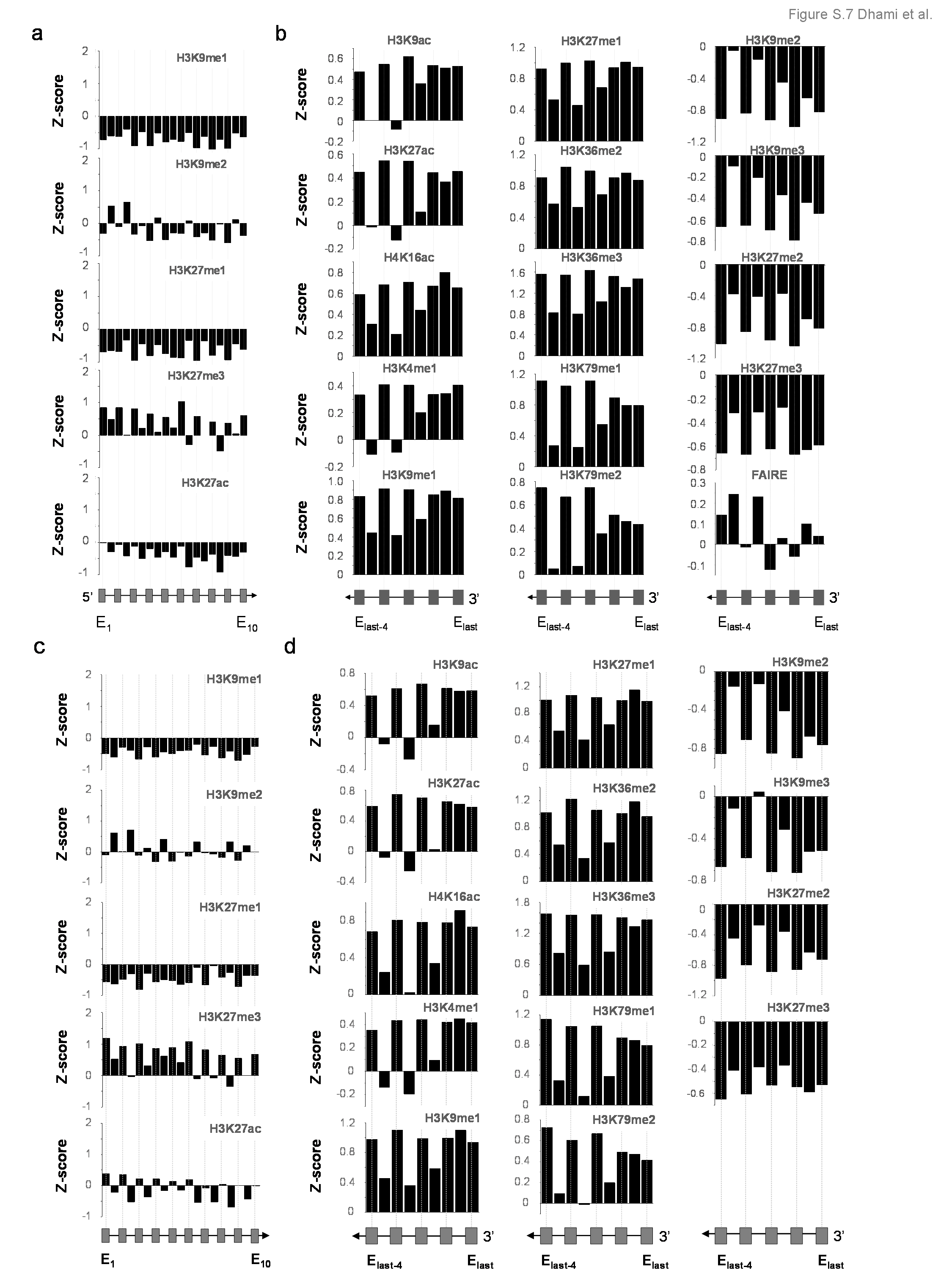

Supplement: Figure S7 — Histone modification and chromatin accessibility (FAIRE) patterns track exons and introns across gene bodies of non-expressed genes and at 3′ ends of expressed genes. Histograms show the mean levels of ChIP-chip enrichments for histone modifications or FAIRE values (Z-scores) spanning the first ten exons and nine introns or last five exons and four introns of consensus genes (hypothetical gene structures are shown at the bottom of each panel of the figure). Data is derived from ENCODE regions in the K562 and U937 cell lines and CD14+ primary monocytes. a. Five histone modifications across first 10 exons and 9 introns of non-expressed genes with histone normalization (n = 92, exons∶introns = 393∶136). b. 14 histone modifications and FAIRE levels across last five exons and four introns of expressed genes with histone normalization (n = 268, exons∶introns = 848∶226). c. Five histone modifications across first ten exons and nine introns of non-expressed genes without histone normalization (n = 92, exons∶introns = 393∶136). d. 14 histone modifications and FAIRE levels across last five exons and four introns of expressed genes without histone normalization (n = 268, exons∶introns = 848∶226). Median P-values obtained from bootstrapping for exons and introns across all patterns shown were <1.0×10^−15^ (panel a), <1.0×10^−15^ (panel b), <1.0×10^−15^ (panel c), and <1.0×10^−15^ (panel d). Median P-values obtained for pair-wise t-tests between adjacent exon-intron pairs were 3.15×10^−2^ (panel a), 4.40×10^−4^ (panel b), 1.35×10^−2^ (panel c), and 1.49×10^−7^ (panel d). (1.27 MB TIF) [file pone.0012339.s008.tif]

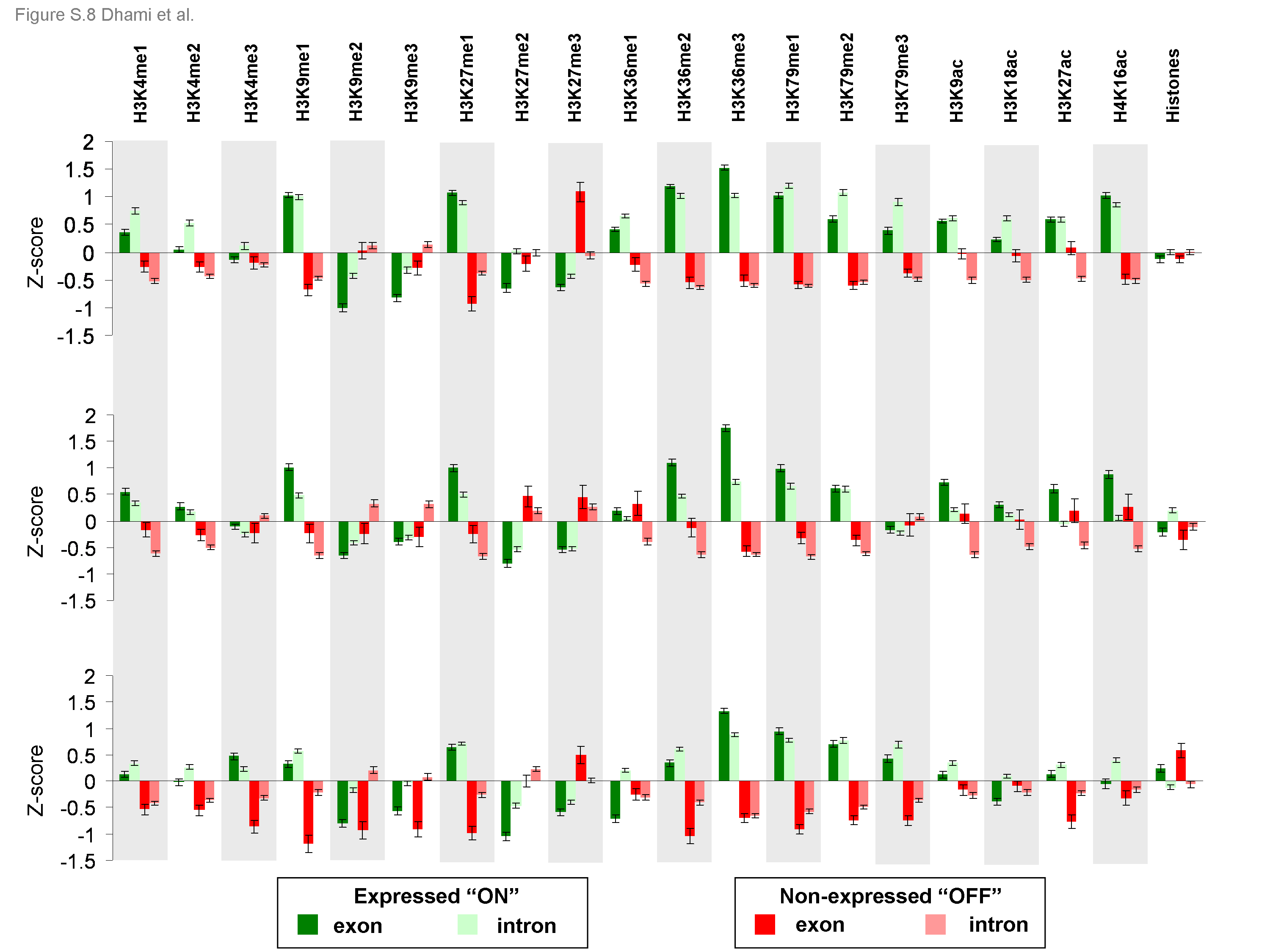

Supplement: Figure S8 — Cell type specificity of exon-intron marking by histone modifications cannot be accounted for by nucleosome distributions. Histograms show the mean levels of ChIP-chip enrichments (Z-scores) for histone modifications across exons and introns of consensus expressed (ON) (green) or non-expressed (OFF) genes (red). Data is derived from ENCODE regions in the K562 and U937 cell lines and CD14+ primary monocytes and each cell line is shown in separate panels. Histone modifications assayed are shown along the top of the figure. Levels of histones are also shown for each cell line at the right of the figure. Data for each cell line is derived as follows. K562 expressed genes (n = 76, exons/introns = 713/290), non-expressed genes (n = 25, exons/introns = 134/76); U937 - expressed genes (n = 88, exons/introns = 801/327), non-expressed genes (n = 20, exons/introns = 128/75); CD14+ monocytes - expressed genes (n = 80, exons/introns = 681/285), non-expressed genes (n = 27, exons/introns = 228/98). All exonic levels were determined for canonical exons only. Error bars are 95% confidence intervals. (1.02 MB TIF) [file pone.0012339.s009.tif]

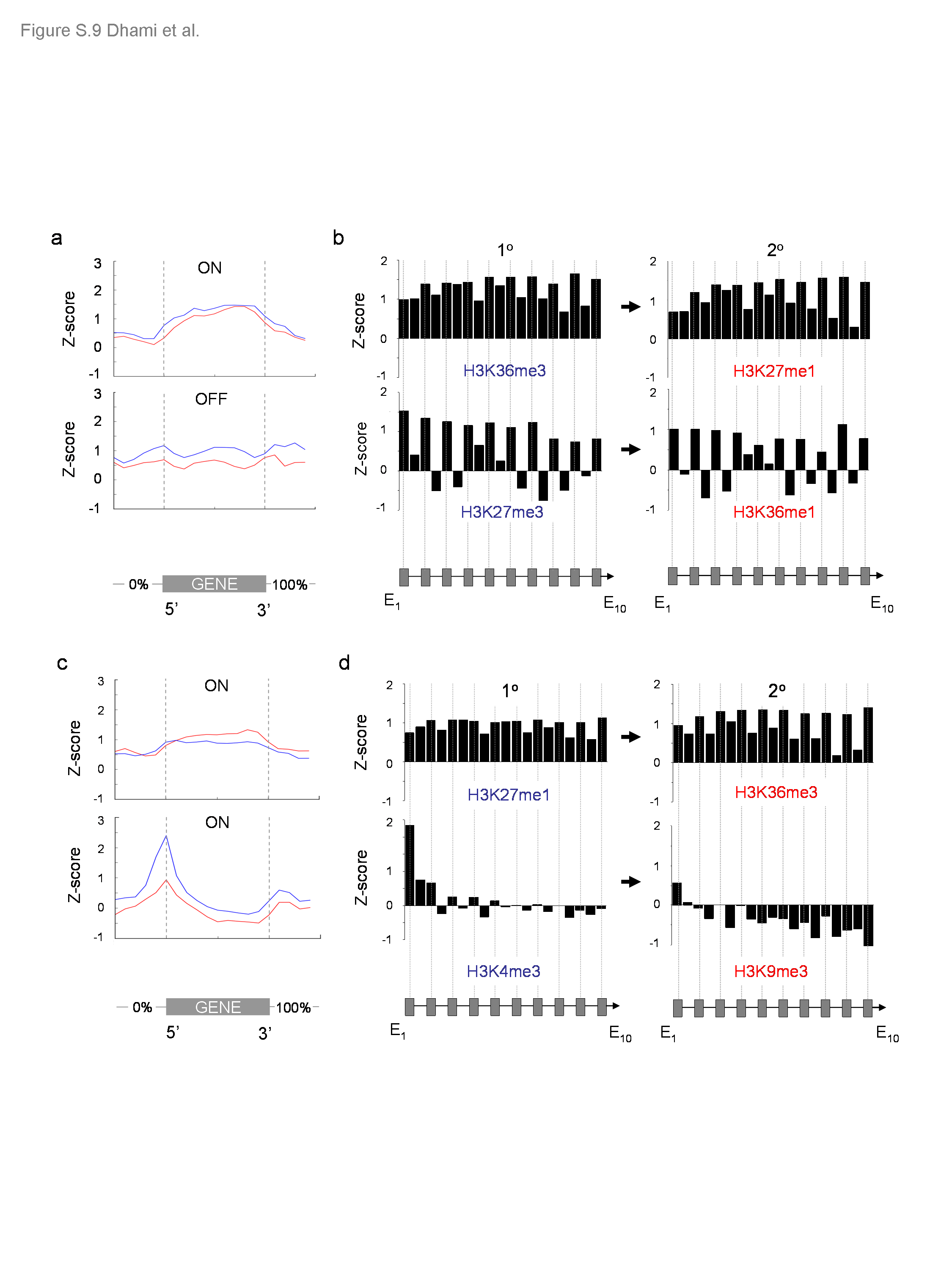

Supplement: Figure S9 — Sequential ChIP-chip enhances histone modification tracking of exon-intron structures in the K562 cell line. Sequential-ChIP-chip was performed using two combinations of primary (1°, blue) and secondary (2°, red) ChIP assays which showed exon-intron tracking across gene bodies (panels a and b). Two control sequential ChIP-chip experiments were also performed (panels c and d). In all cases, data was analyzed to take into account nucleosome distribution (i.e., normalized with respect to histone H2B and H3 density). a. Consensus gene plot showing mean enrichment levels (Z-scores) across expressed (ON) (n = 111) or non-expressed (OFF) genes (n = 53) in the K562 cell line from ENCODE regions: 1° with anti-H3K36me3 and sequential 2° with anti-H3K27me1 (top panel); 1° with anti-H3K27me3 and sequential 2° with anti-H3K36me1 (bottom panel). Proportional gene length and flanking regions are shown on the x axis as percentages (%). b. Histograms show the levels of combinations of histone modifications across the first ten exons and nine introns of consensus expressed (ON) genes (n = 85, exons∶introns = 499∶185) or non-expressed (OFF) genes (n = 26, exons∶introns = 132∶54) from panel a. Hypothetical gene structures are shown below the panel. Median P-values obtained from bootstrapping for exons and introns were <1.0×10^−15^ (expressed genes) and <1.0×10^−15^ (non-expressed genes). Median P-values obtained for pair-wise t-tests between adjacent exon-intron pairs (exon2 → exon10) were 3.63×10^−4^ (expressed genes) and 1.24×10^−3^ (non-expressed genes). c. Consensus gene plot showing mean enrichment levels (Z-scores) across expressed (ON) (n = 111) genes in the K562 cell line from ENCODE regions: 1° with anti-H3K27me1 and sequential 2° with anti-H3K36me3 (top panel); 1° with anti-H3K4me3 and sequential 2° with anti-H3K9me3 (bottom panel). d. Histograms show the levels of combinations of histone modifications across the first ten exons and nine introns of consensus expressed genes (ON) (n = [file pone.0012339.s010.tif]

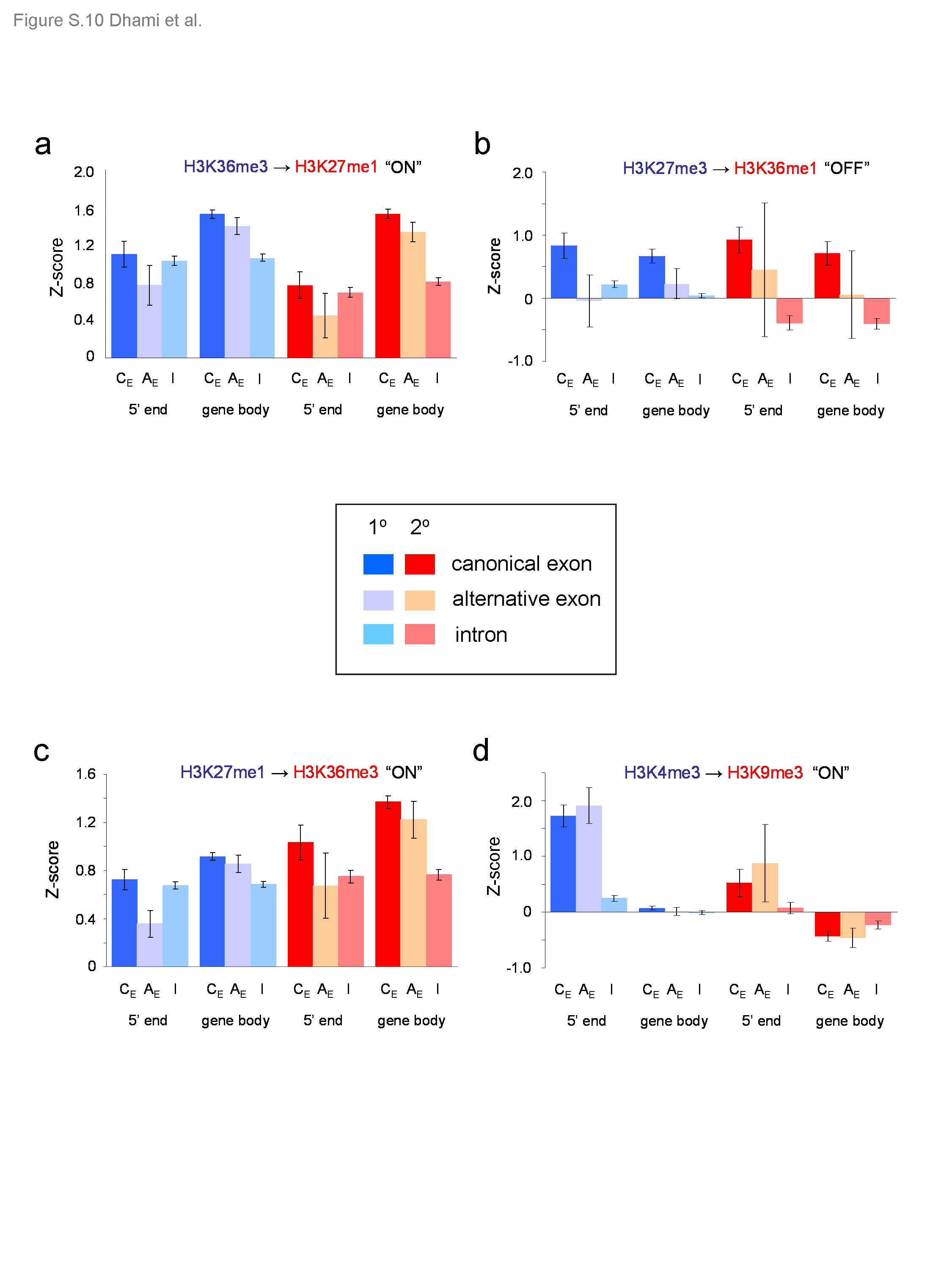

Supplement: Figure S10 — Histograms show the levels of sequential-ChIP-chip enrichments for combinations of histone modifications (those described in Figure S9) spanning typical canonical/alternatively-spliced exons (CE and AE respectively) and introns (I) of expressed genes [n = 85, canonical exons:alternatively-spliced exons∶introns = 145∶68∶221 (5′ ends) or 796∶166∶976 (gene bodies)]. Blue bars show ChIP-chip enrichments after 1° antibody and red bars show ChIP-chip enrichment after the 2° antibody. Error bars are 95% confidence intervals. In all panels, histone modification ChIP-chip enrichment levels are expressed as mean Z-scores. (0.84 MB TIF) [file pone.0012339.s011.tif]

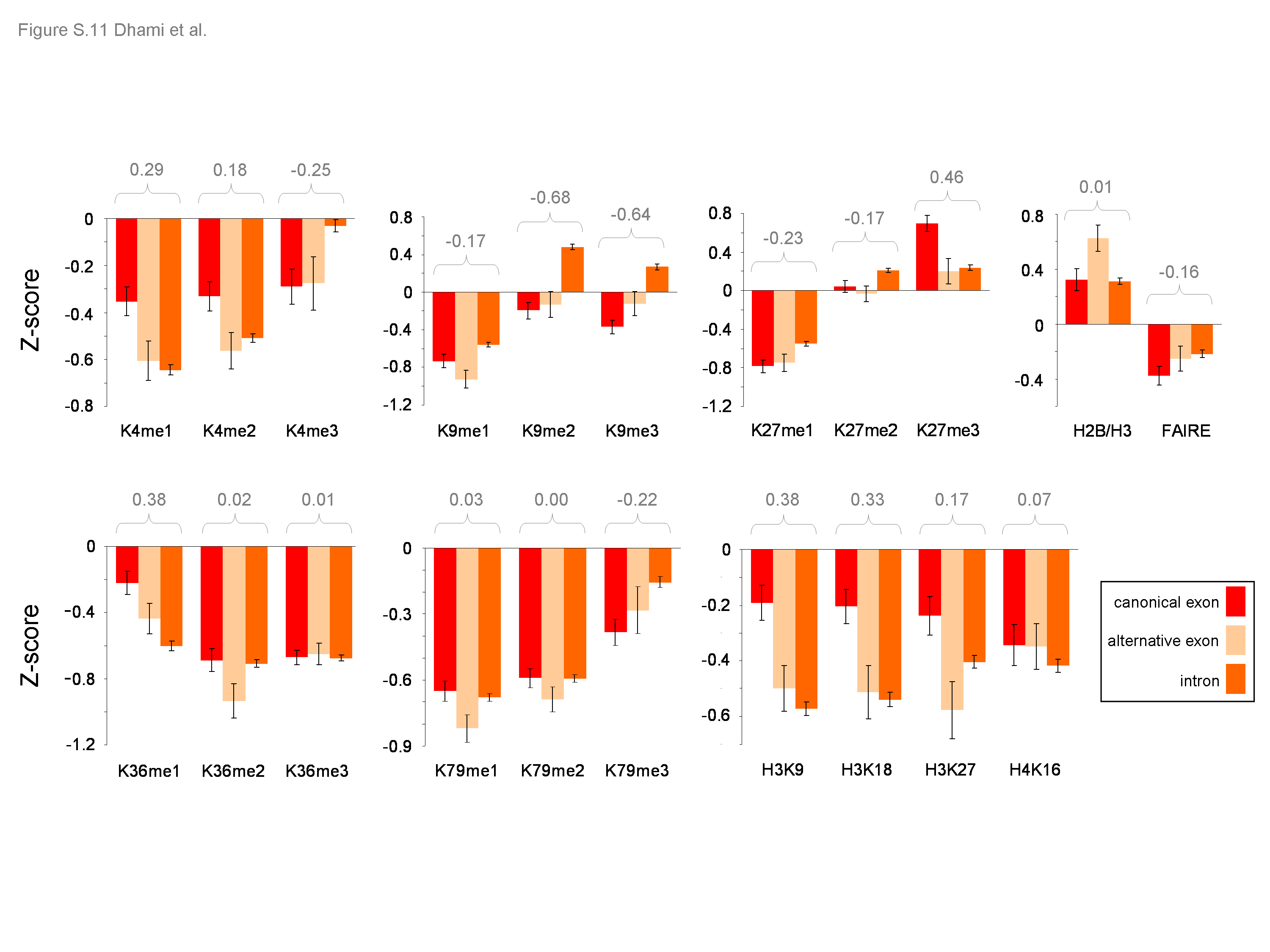

Supplement: Figure S11 — Histone modifications differentially mark canonical and alternatively-spliced exons and introns across non-expressed genes. Histograms show the mean levels (Z-scores) for histone modifications and histones (ChIP-chip enrichments) or chromatin accessibility (FAIRE) spanning typical canonical/alternatively-spliced exons and introns. Data was derived from gene bodies of non-expressed genes (n = 92, canonical exons:alternatively-spliced exons:introns = 631∶184∶826) in the K562 and U937 cell lines and CD14+ primary monocytes across the ENCODE regions. Histone distribution was based on the combined data for H2B and H3 in each cell type. Biases favoring either canonical exon or intron are summarized by the difference in Z-scores shown above each assay in grey. Positive (+) differences in Z-scores reflect exon biases, while negative (−) differences reflect intron biases. Error bars are 95% confidence intervals. (0.76 MB TIF) [file pone.0012339.s012.tif]

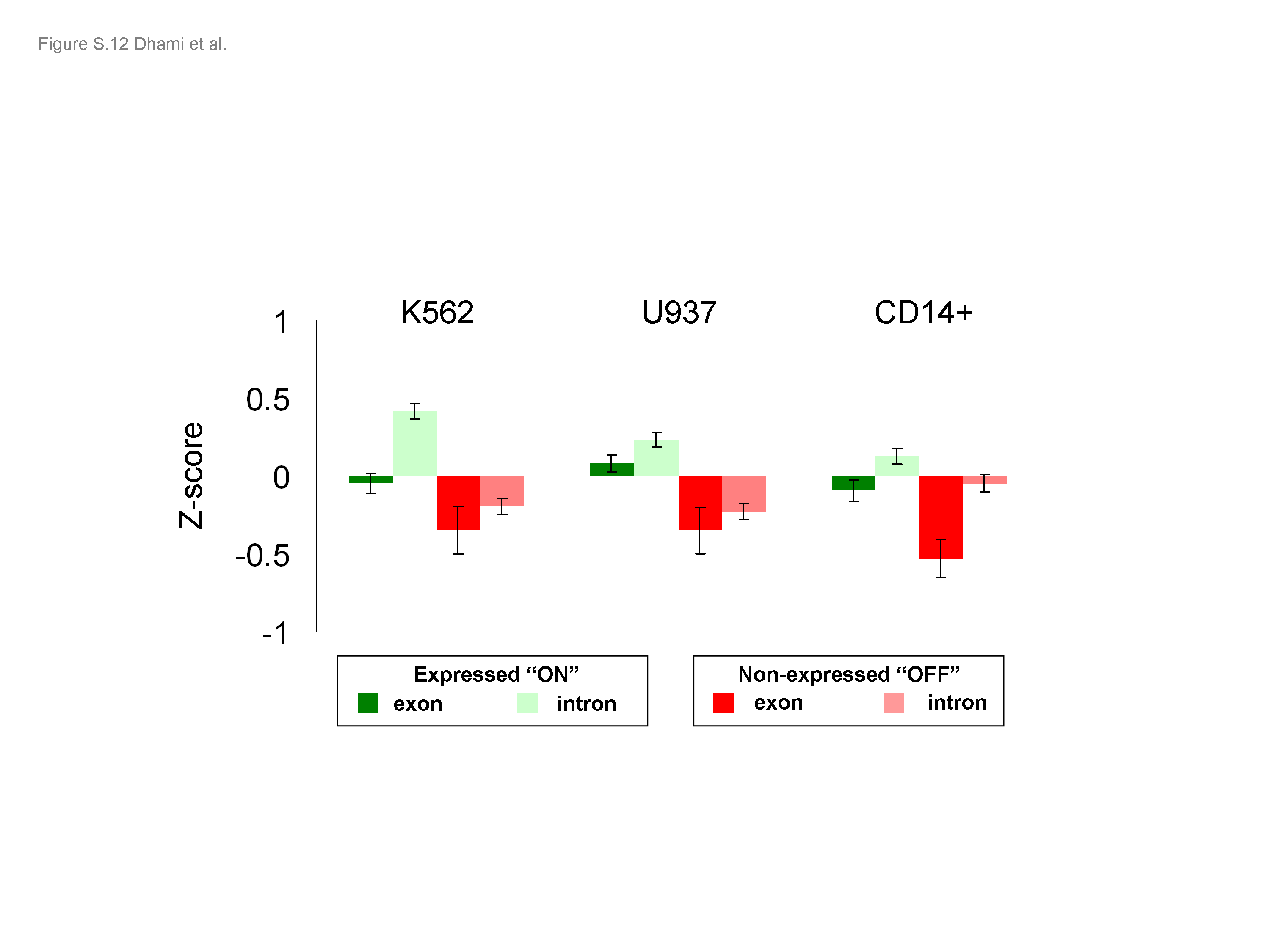

Supplement: Figure S12 — FAIRE accessibility assays show introns are preferentially accessible across three cell types. Histograms show the mean levels of FAIRE enrichments (Z-scores) across exons and introns of consensus expressed (ON) (green) or non-expressed (OFF) genes (red). Data is derived from ENCODE regions in the K562 and U937 cell lines and CD14+ primary monocytes and each cell line is shown separately. Datapoints for each cell line were derived as follows. K562 expressed genes (n = 76, exons/introns = 713/290), non-expressed genes (n = 25, exons/introns = 134/76); U937 - expressed genes (n = 88, exons/introns = 801/327), non-expressed genes (n = 20, exons/introns = 128/75); CD14+ monocytes - expressed genes (n = 80, exons/introns = 681/285), non-expressed genes (n = 27, exons/introns = 228/98). All exonic levels were determined for canonical exons only. Error bars are 95% confidence intervals. (0.50 MB TIF) [file pone.0012339.s013.tif]

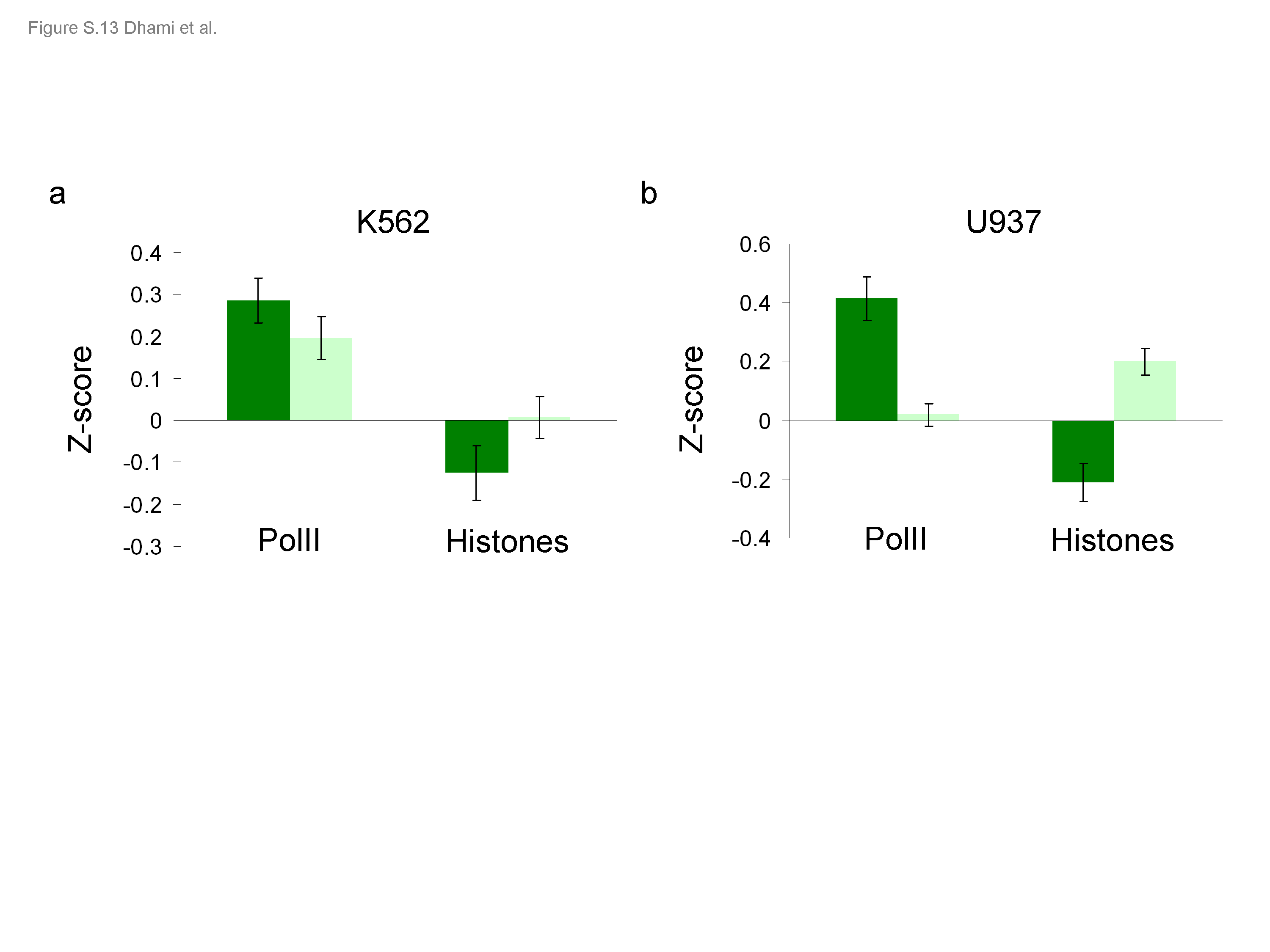

Supplement: Figure S13 — RNA polymerase II (Pol II) occupancy levels are not accounted for by nucleosome distributions. Histograms show the mean levels of ChIP-chip enrichments (Z-scores) for Pol II and histones across exons and introns of consensus expressed (ON) (green) or non-expressed (OFF) genes (red). a. Data derived from ENCODE regions in the K562 cell line: expressed genes (n = 76, exons/introns = 707/287), non expressed genes (n = 25, exons/introns = 133/76). b. Data derived from U937 cell line: expressed genes (n = 88, exons/introns = 797/325), non expressed genes (n = 20, exons/introns = 123/73). All exonic levels were determined for canonical exons only. Error bars are 95% confidence intervals. (0.52 MB TIF) [file pone.0012339.s014.tif]

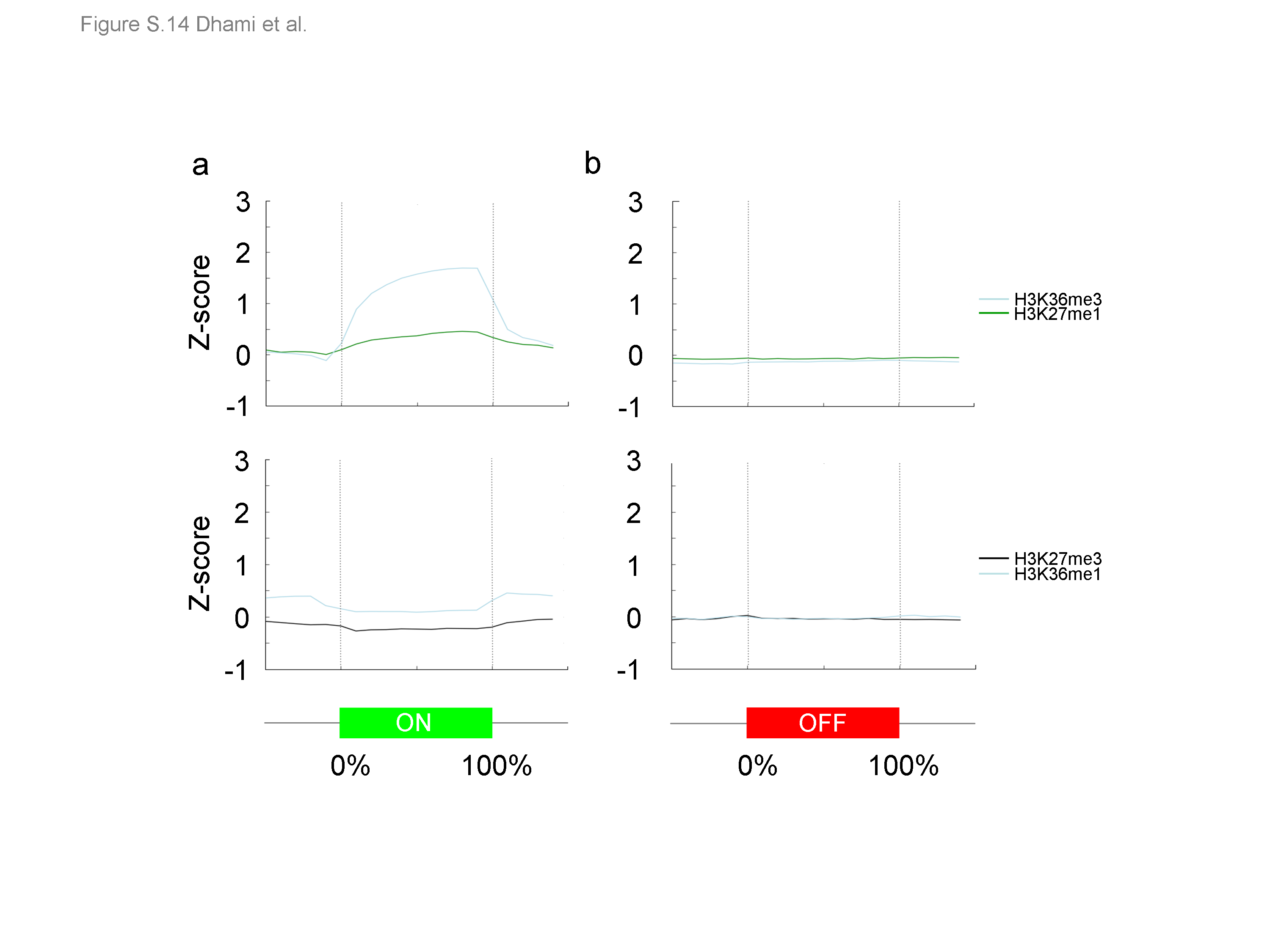

Supplement: Figure S14 — Genome-wide patterns of H3K27me1, H3K27me3, H3K36me1 and H3K36me3 for expressed and non-expressed genes in the K562 cell line. a. Consensus gene plots for four histone modifications across expressed (ON) genes (n = 2066). b. Consensus gene plots for four histone modifications across non-expressed (OFF) genes (n = 1973). ChIP-chip enrichment levels in both panels are expressed as mean Z-scores. Proportional gene length and flanking regions are shown on the x axis as percentages (%). Color key to modifications depicted in each panel are shown to the right of the figure. (0.60 MB TIF) [file pone.0012339.s015.tif]

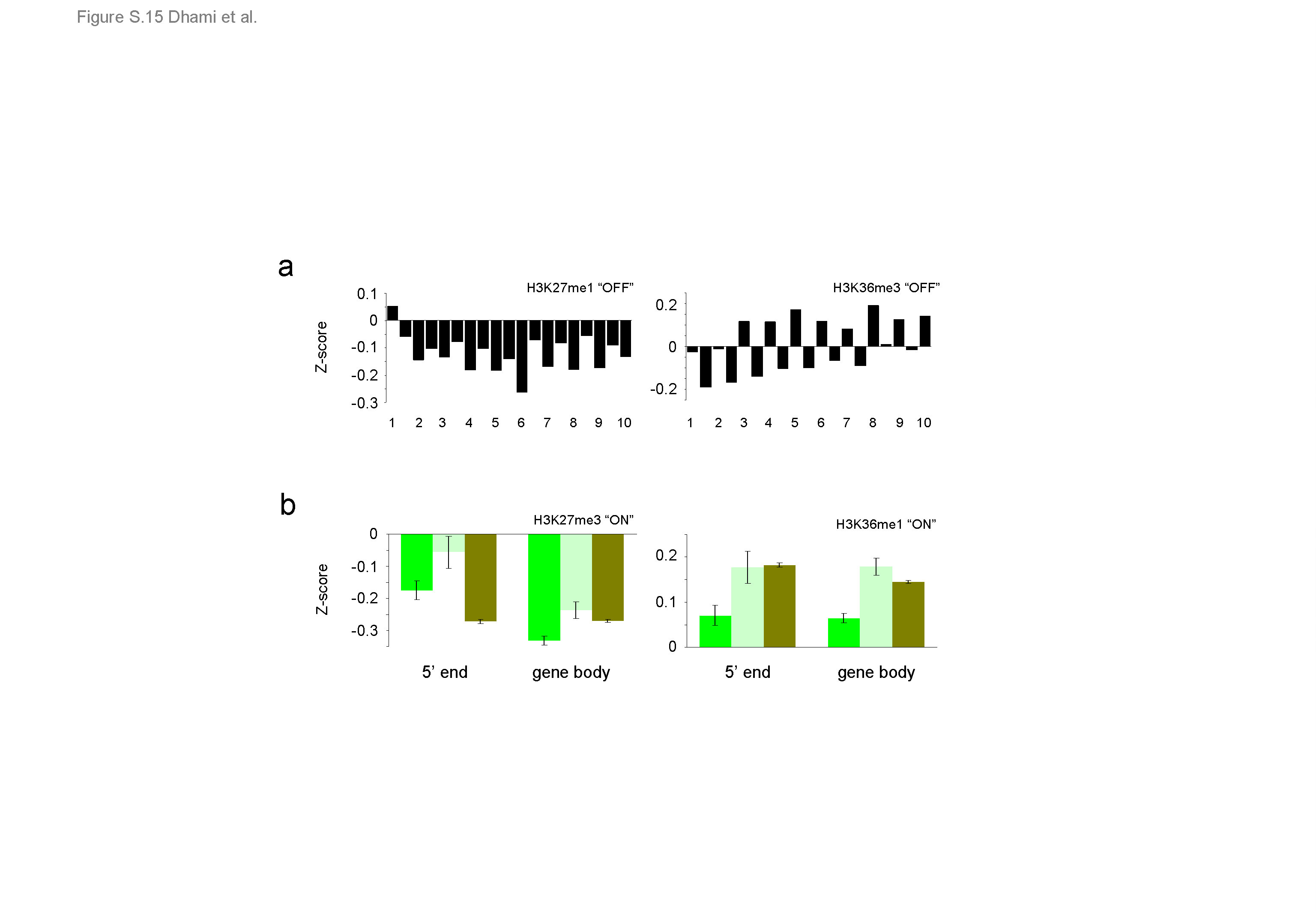

Supplement: Figure S15 — Histone modifications patterns mark exon-intron structures across the whole human genome. a. Histograms show the level of H3K27me1 (bin 0%-10%, n = 662, exon∶introns = 4378∶3697) and H3K36me3 (bin 0%–25%, n = 1657, exon∶introns = 10911∶9194) across the first ten exons and nine introns of consensus non-expressed (OFF) genes. Exon numbering is at the bottom of panel. b. Histograms show the levels of ChIP-chip enrichments for H3K27me3 [bin 95%–100%, n = 332, canonical exons:alternatively-spliced exons∶introns = 642∶165∶811 (5′ ends) or 2415∶402∶2817 (gene bodies)] and H3K36me1 [bin 90%–100%, n = 700, canonical exons:alternatively-spliced exons∶introns = 1385∶400∶1803 (5′ ends) or 5750∶882∶6649 (gene bodies)] spanning typical canonical (dark green)/alternatively-spliced (light green) exons and introns (olive green) of expressed (ON) genes. Error bars are 95% confidence intervals. In both panels, ChIP-chip enrichments obtained from genome-wide analysis of the K562 cell line are expressed as mean Z-scores. (0.51 MB TIF) [file pone.0012339.s016.tif]

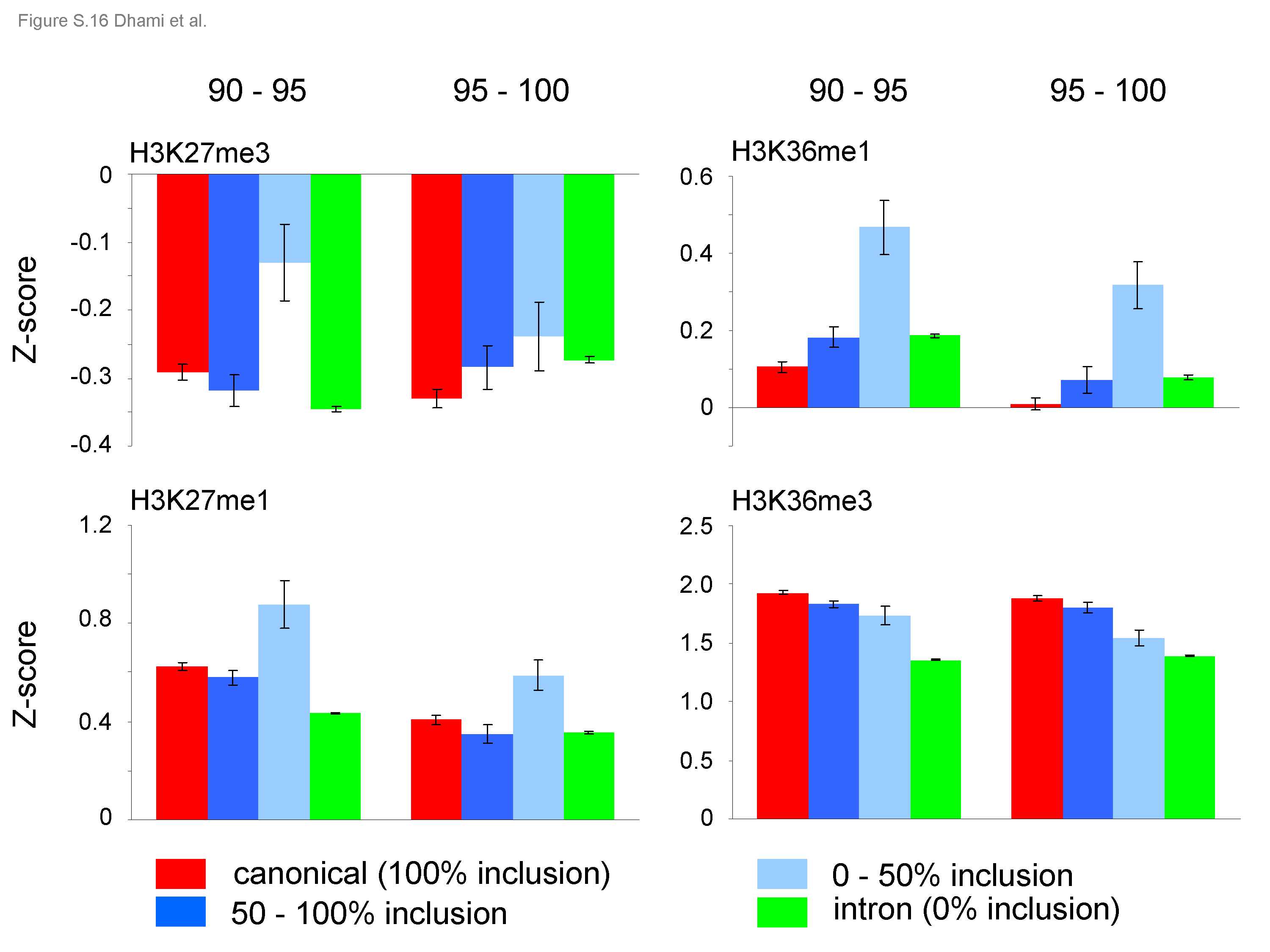

Supplement: Figure S16 — Histone modification patterns show relationships with either exon inclusion or with exon exclusion within gene bodies of highly expressed genes. Histograms show the levels of four histone modifications for the top ten percent of expressed genes in the K562 cell line. Genes (991) were placed into two bins: 90–95% and 95%–100% based on ranked expression level. Analysis shown was based on canonical exons = 6967, introns = 7714, and alternatively-spliced exons 221: 1040 (0–50% inclusion: 50–100% inclusion). Error bars are 95% confidence intervals. In all panels, ChIP-chip enrichments obtained from genome-wide analysis of the K562 cell line are expressed as mean Z-scores. (0.93 MB TIF) [file pone.0012339.s017.tif]

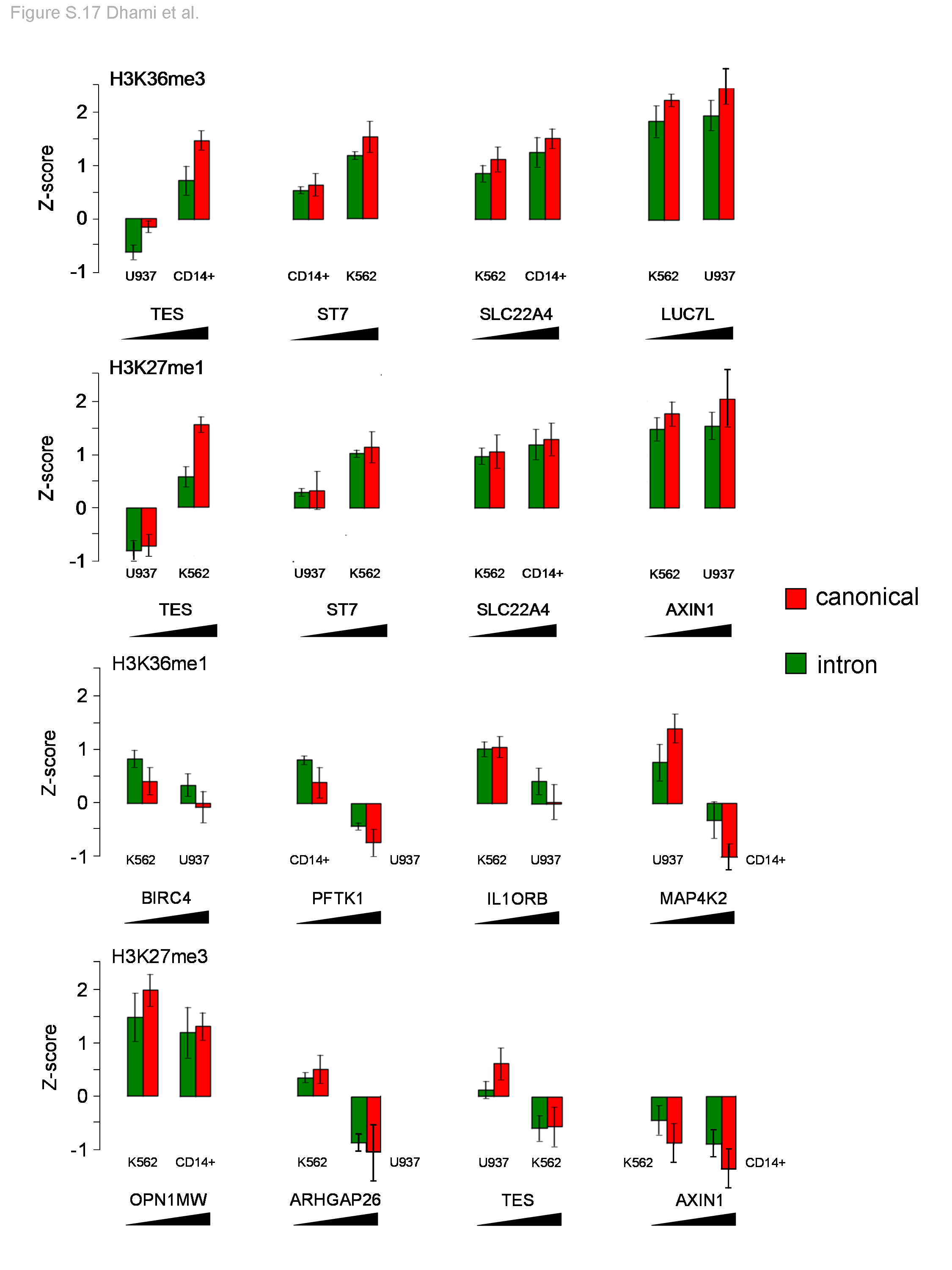

Supplement: Figure S17 — Changes in gene expression levels are accompanied by changes in histone modification levels across gene bodies. Levels of H3K36me3, H3K27me1, H3K36me1, and H3K27me3 for genes which show differential expression between cell types (K562, U937 and CD14+) are shown. Histone modification ChIP-chip enrichment levels (Z-scores) for canonical exons (red) and introns (green) are shown on the y axis. Gene names and their expression levels in two different cell types (level of expression - low → high or off → on - denoted by the black triangle) are shown below the x axis. H3K36me3 and H3K27me1 both show exon enrichment biases for all differentially-expressed gene pairs shown. However, both H3K36me1 and H3K27me3 show either exon or intron enrichment biases depending on the level of gene expression, which are consistent with the whole genome datasets shown in Figure 5. Error bars are 95% confidence intervals. (1.13 MB TIF) [file pone.0012339.s018.tif]

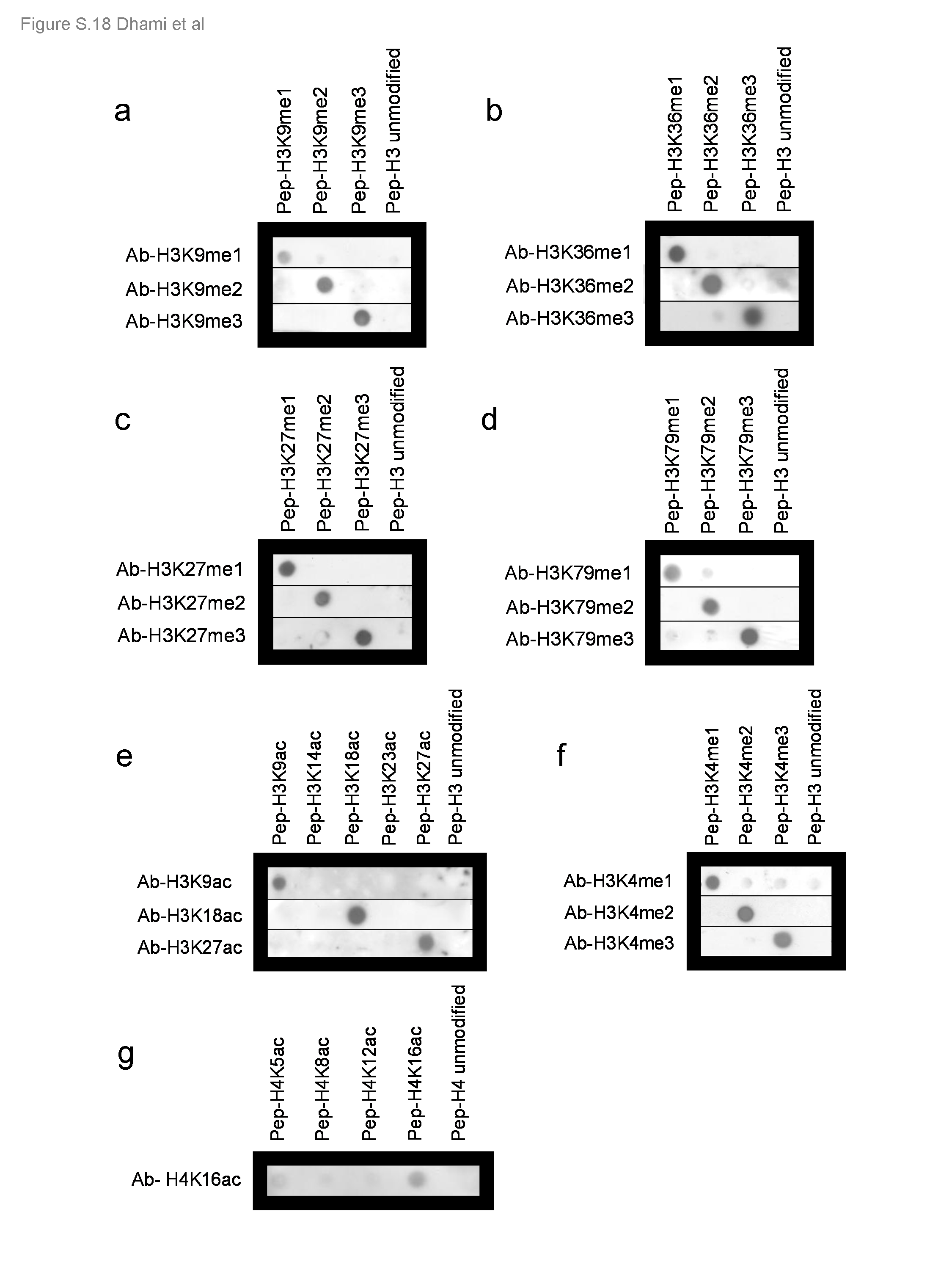

Supplement: Figure S18 — Determination of specificity of antibodies used in this study by dot blot analysis. Antibodies raised against histone modifications were each hybridized to a panel of relevant methyl-modified or acetyl modified peptides of histone H3 and histone H4 and their unmodified forms (see also Materials and Methods). Antibodies (Ab) and peptides (Pep) used are shown on the left and top of each panel respectively. Images in each panel are composites of different hybridizations denoted by the black lines dividing the sections of the panels. a. H3K9 methyl modifications. b. H3K36 methyl modifications. c. H3K27 methyl modifications. d. H3K79 methyl modifications. e. H3K9, 18, 27 acetyl modifications. f. H3K4 methyl modifications. g. H4K16 acetyl modification. In all panels, the results are shown for peptides spotted onto the immunoblot at a concentration of 25 ng/µl. (1.44 MB TIF) [file pone.0012339.s019.tif]
