# Supplementary material for: Complex Exon-Intron Marking by Histone Modifications Is Not Determined Solely by Nucleosome Distribution
Source: PLoS One. 2010 Aug 23;5(8):e12339. doi: 10.1371/journal.pone.0012339 (PMC2925886; doi:10.1371/journal.pone.0012339)
Supplement: Table S2 — Antibodies and peptides used in this ChIP-chip study. a. The name of the epitopes to which each antibody used in ChIP-chip is given in the first column. The supplier and the catalogue number of each antibody are given in the second and third columns respectively. Lot numbers of each antibody appears in the last column. b. The names of peptides used in dot blot analysis are shown in the first column. The supplier and the catalogue number of each peptide are given in the second and third columns respectively. (0.08 MB DOC) [file pone.0012339.s021.doc]

**a.**

| **ANTIBODY EPITOPE** | **SUPPLIER** | **CATALOGUE NUMBER** | **LOT NUMBER** |
| --- | --- | --- | --- |
| Rabbit IgG | Millipore (Upstate) | 12-370 | DAM1421465 |
| Mouse IgG | Millipore (Upstate) | 12-371 | 32714 |
| RNA polymerase II | Abcam | ab5408 | 75815 |
| histone H3 acetyl K9 | Millipore (Upstate) | 07-352 | 31388 |
| histone H3 acetyl K18 | Millipore (Upstate) | 07-354 | 27102 |
| histone H3 acetyl K27 | Millipore (Upstate) | 07-360 | 26817 |
| histone H4 acetyl K16 | Abcam | ab1762 | 131051 |
| histone H3 monomethyl K4 | Abcam | ab8895 | 70938 |
| histone H3 dimethyl K4 | Abcam | ab7766 | 56290 |
| histone H3 trimethyl K4 | Abcam | ab8580 | 412671 |
| histone H3 monomethyl K9 | Abcam | ab9045 | 247931 |
| histone H3 dimethyl K9 | Millipore (Upstate) | 07-212 | 27253 |
| histone H3 trimethyl K9 | Millipore (Upstate) | 07-523 | 27759 |
| histone H3 monomethyl K27 | Millipore (Upstate) | 07-448 | 24439 |
| histone H3 dimethyl K27 | Abcam | ab24684 | 196569 |
| histone H3 trimethyl K27 | Millipore (Upstate) | 07-449 | DAM1421462 |
| histone H3 monomethyl K36 | Abcam | ab9048 | 206009 |
| histone H3 dimethyl K36 | Millipore (Upstate) | 07-274 | 31553 |
| histone H3 trimethyl K36 | Abcam | ab9050 | 690604 |
| histone H3 monomethyl K79 | Abcam | ab2886 | 17647 |
| histone H3 dimethyl K79 | Abcam | ab3594 | 19752 |
| histone H3 trimethyl K79 | Abcam | ab2621 | 456837 |
| histone H3 | Abcam | ab1791 | 624416 |
| histone H2B | Abcam | ab1790 | 632626 |

**b.**

| **PEPTIDE +/- MODIFICATION** | **SUPPLIER** | **CATALOGUE NUMBER** |
| --- | --- | --- |
| H3 K9ac | Millipore (Upstate) | 12-358 |
| H3K14ac | Millipore (Upstate) | 12-359 |
| H3 K18ac | Abcam | ab24003 |
| H3K23ac | Cell Signalling Technology | CO1-955 |
| H3 K27ac | Abcam | ab24404 |
| H4 K5ac | Millipore (Upstate) | 12-343 |
| H4 K8ac | Millipore (Upstate) | 12-344 |
| H4 K12ac | Millipore (Upstate) | 12-345 |
| H4 K16ac | Millipore (Upstate) | 12-346 |
| H3 K4me1 | Abcam | ab1340 |
| H3 K4me2 | Abcam | ab7768 |
| H3 K4me3 | Abcam | ab1342 |
| H3 K9me1 | Abcam | ab1771 |
| H3 K9me2 | Abcam | ab1772 |
| H3 K9me3 | Abcam | ab1773 |
| H3 K27me1 | Abcam | ab1780 |
| H3 K27me2 | Abcam | ab1781 |
| H3 K27me3 | Abcam | ab1782 |
| H3 K36me1 | Abcam | ab1783 |
| H3 K36me2 | Abcam | ab1784 |
| H3 K36me3 | Abcam | ab1785 |
| H3 K79me1 | Abcam | ab4555 |
| H3 K79me2 | Abcam | ab4556 |
| H3 K79me3 | Abcam | ab4557 |
| H3 unmodified (residues 1-16) | Abcam | ab7228 |
| H3 unmodified | Millipore (Upstate) | 12-357 |
| H3 unmodified (residues 25-36) | Abcam | ab14794 |
| H3 unmodified (residues 74-83) | Abcam | ab4558 |
| H4 unmodified | Millipore (Upstate) | 12-347 |
