# Supplementary material for: Complex Exon-Intron Marking by Histone Modifications Is Not Determined Solely by Nucleosome Distribution
Source: PLoS One. 2010 Aug 23;5(8):e12339. doi: 10.1371/journal.pone.0012339 (PMC2925886; doi:10.1371/journal.pone.0012339)
Supplement: Table S4 — Non-expressed genes in the K562 cell line across the ENCODE regions. Non-expressed genes were determined as described in Materials and Methods and this list reflects the intersecting bottom quartile of expression values obtained from Affymetrix GeneChip® and Sanger Institute microarray expression studies. Gene ID/name is shown in the first column. The ENCODE region, chromosome co-ordinates [(NCBI human genome build 35 (hg17)] and direction of transcript/strand are also shown in the additional columns. (0.24 MB DOC) [file pone.0012339.s023.doc]

| **Gene ID** | **Region** | **Chr** | **Start** | **End** | **Strand** |
| --- | --- | --- | --- | --- | --- |
| AC002064.6 | ENm013 | 7 | 89609189 | 89618060 | 1 |
| AC005592.1 | ENr212 | 5 | 142105350 | 142120748 | 1 |
| AC006293.1 | ENm007 | 19 | 59912199 | 59916547 | 1 |
| AC006293.3 | ENm007 | 19 | 59913804 | 59916768 | 1 |
| AC006985.5 | ENr131 | 2 | 234466371 | 234524070 | 1 |
| AC008440.10 | ENm007 | 19 | 59059830 | 59061207 | 1 |
| AC008746.10 | ENm007 | 19 | 59582906 | 59583377 | 1 |
| AC008746.9 | ENm007 | 19 | 59724719 | 59725027 | 1 |
| AC008940.1 | ENr221 | 5 | 56102384 | 56103881 | 1 |
| AC009892.2 | ENm007 | 19 | 59755066 | 59755352 | 1 |
| AC009892.5 | ENm007 | 19 | 59811150 | 59811656 | 1 |
| AC009892.8 | ENm007 | 19 | 59851206 | 59852115 | 1 |
| AC009892.9 | ENm007 | 19 | 59855348 | 59855493 | 1 |
| AC009955.5 | ENr331 | 2 | 220240957 | 220261749 | -1 |
| AC011330.12 | ENr233 | 15 | 41678883 | 41684392 | 1 |
| AC011501.2 | ENm007 | 19 | 59958288 | 59970636 | 1 |
| AC011501.4 | ENm007 | 19 | 59989728 | 59993583 | 1 |
| AC011515.2 | ENm007 | 19 | 59874495 | 59875855 | 1 |
| AC011515.3 | ENm007 | 19 | 59900196 | 59904496 | 1 |
| AC023590.1 | ENr321 | 8 | 119363663 | 119377115 | 1 |
| AC051649.12 | ENm011 | 11 | 1841984 | 1844474 | 1 |
| AC079630.2 | ENr123 | 12 | 38836309 | 38847777 | 1 |
| AC092402.4 | ENm006 | X | 152982660 | 153003603 | -1 |
| AC092402.5 | ENm006 | X | 153006120 | 153019603 | 1 |
| AC098784.1 | ENm007 | 19 | 59907543 | 59907826 | 1 |
| AC104389.16 | ENm009 | 11 | 5182848 | 5185115 | 1 |
| AC114812.10 | ENr131 | 2 | 234441264 | 234444303 | 1 |
| AC114812.9 | ENr131 | 2 | 234429599 | 234432604 | 1 |
| AF064861.94 | ENr133 | 21 | 39610956 | 39617015 | 1 |
| AFF4 | ENm002 | 5 | 132238971 | 132267996 | -1 |
| AL163953.1 | ENr311 | 14 | 53149193 | 53149874 | 1 |
| AP000269.3 | ENm005 | 21 | 32747874 | 32752640 | 1 |
| AP000282.3 | ENm005 | 21 | 33253067 | 33254745 | 1 |
| AP000288.2 | ENm005 | 21 | 33352006 | 33359160 | 1 |
| AP000569.2 | ENm005 | 21 | 34243101 | 34258131 | 1 |
| AP001092.5 | ENr332 | 11 | 64175344 | 64180360 | 1 |
| AP002856.7 | ENr312 | 11 | 130609644 | 130626922 | 1 |
| AP003774.5 | ENr332 | 11 | 63952210 | 63956719 | 1 |
| AP005273.1 | ENr332 | 11 | 64024902 | 64029435 | 1 |
| AP006288.1 | ENr332 | 11 | 64055093 | 64057019 | 1 |
| APOC3 | ENm003 | 11 | 116205633 | 116208999 | 1 |
| ARHGAP26 | ENr212 | 5 | 142130134 | 142374156 | 1 |
| C21orf55 | ENm005 | 21 | 33779708 | 33785898 | -1 |
| C21orf62 | ENm005 | 21 | 33087747 | 33107924 | -1 |
| C21orf63 | ENm005 | 21 | 32706186 | 32809571 | 1 |
| C9orf106 | ENr232 | 9 | 129162850 | 129166739 | 1 |
| CACNG8 | ENm007 | 19 | 59158107 | 59185282 | 1 |
| CKMT1 | ENr233 | 15 | 41774623 | 41775340 | 1 |
| CKMT1A | ENr233 | 15 | 41772377 | 41778713 | 1 |
| CKMT1B | ENr233 | 15 | 41672545 | 41678897 | 1 |
| CTAG1A | ENm006 | X | 153377112 | 153378780 | 1 |
| CTGF | ENr222 | 6 | 132311010 | 132314207 | -1 |
| CXorf2 | ENm006 | X | 153019778 | 153044644 | -1 |
| CXorf2B | ENm006 | X | 152945530 | 152966482 | -1 |
| CYP4A22 | SCL | 1 | 47315128 | 47327434 | 1 |
| CYP4Z1 | SCL | 1 | 47245181 | 47296012 | 1 |
| F7 | ENr132 | 13 | 112808107 | 112822997 | 1 |
| FSCN3 | ENm014 | 7 | 126825415 | 126835804 | 1 |
| GRM8 | ENm014 | 7 | 125672608 | 126487300 | -1 |
| H2AFB1 | ENm006 | X | 153676952 | 153677538 | 1 |
| HOXA1 | ENm010 | 7 | 26905853 | 26908834 | -1 |
| HOXA2 | ENm010 | 7 | 26913217 | 26915546 | -1 |
| HOXA5 | ENm010 | 7 | 26953912 | 26956704 | -1 |
| HOXA6 | ENm010 | 7 | 26958256 | 26965458 | -1 |
| HOXA7 | ENm010 | 7 | 26966576 | 26970796 | -1 |
| HOXA9 | ENm010 | 7 | 26975298 | 26988040 | -1 |
| HS3ST4 | ENr211 | 16 | 25805569 | 26056511 | 1 |
| IGF2 | ENm011 | 11 | 2106919 | 2138797 | -1 |
| IGF2AS | ENm011 | 11 | 2118308 | 2126471 | 1 |
| IGLCOR22-1 | ENm004 | 22 | 30920461 | 30920776 | 1 |
| IL5 | ENm002 | 5 | 131905036 | 131920430 | -1 |
| INS | ENm011 | 11 | 2137585 | 2139148 | -1 |
| ITSN1 | ENm005 | 21 | 33936577 | 34194036 | 1 |
| KIF3A | ENm002 | 5 | 132056268 | 132101230 | -1 |
| KIR2DL1 | ENm007 | 19 | 59973076 | 59987311 | 1 |
| KIR2DL3 | ENm007 | 19 | 59941793 | 59956317 | 1 |
| KIR2DL4 | ENm007 | 19 | 60006879 | 60017785 | 1 |
| KIR3DL1 | ENm007 | 19 | 60019736 | 60023280 | 1 |
| KIR3DL3 | ENm007 | 19 | 59927797 | 59939816 | 1 |
| LAIR2 | ENm007 | 19 | 59700913 | 59713710 | 1 |
| LILRA1 | ENm007 | 19 | 59796860 | 59805368 | 1 |
| LILRA2 | ENm007 | 19 | 59776200 | 59790840 | 1 |
| LILRA3 | ENm007 | 19 | 59491667 | 59501765 | -1 |
| LILRA5 | ENm007 | 19 | 59510166 | 59516222 | -1 |
| LILRB1 | ENm007 | 19 | 59820425 | 59840792 | 1 |
| LILRB4 | ENm007 | 19 | 59847153 | 59873623 | 1 |
| LILRB5 | ENm007 | 19 | 59446076 | 59452977 | -1 |
| NCR2 | ENr334 | 6 | 41411372 | 41426604 | 1 |
| NRXN2 | ENr332 | 11 | 64130223 | 64247237 | -1 |
| OLIG1 | ENm005 | 21 | 33364321 | 33366597 | 1 |
| OLIG2 | ENm005 | 21 | 33320024 | 33323375 | 1 |
| OPN1LW | ENm006 | X | 152930593 | 152945355 | 1 |
| OPN1MW | ENm006 | X | 152969002 | 152982481 | 1 |
| OR51A7 | ENm009 | 11 | 4885177 | 4886115 | 1 |
| OR51F2 | ENm009 | 11 | 4799193 | 4800221 | 1 |
| OR51H2P | ENm009 | 11 | 4854364 | 4855269 | 1 |
| OR51L1 | ENm009 | 11 | 4976790 | 4977737 | 1 |
| OR51N1P | ENm009 | 11 | 4764561 | 4765512 | 1 |
| OR51T1 | ENm009 | 11 | 4859707 | 4860690 | 1 |
| OR52A1 | ENm009 | 11 | 5128816 | 5164189 | -1 |
| OR52E1P | ENm009 | 11 | 5047379 | 5048304 | 1 |
| OR52E3P | ENm009 | 11 | 5070483 | 5071418 | 1 |
| OR52J1P | ENm009 | 11 | 5081960 | 5082891 | 1 |
| OR52J2P | ENm009 | 11 | 5014820 | 5015758 | 1 |
| OR52J3 | ENm009 | 11 | 5024333 | 5025268 | 1 |
| OR52U1P | ENm009 | 11 | 5697097 | 5698019 | 1 |
| OR56B1 | ENm009 | 11 | 5686015 | 5715298 | 1 |
| OSTM1 | ENr323 | 6 | 108469307 | 108502639 | -1 |
| RFPL2 | ENm004 | 22 | 30910980 | 30924019 | -1 |
| RFPL3 | ENm004 | 22 | 31075427 | 31081703 | 1 |
| RGS11 | ENm008 | 16 | 258302 | 265982 | -1 |
| RP11-257K9.7 | ENr223 | 6 | 73989989 | 73992215 | -1 |
| RP11-298J23.5 | ENr334 | 6 | 41796113 | 41809749 | 1 |
| RP11-328M4.3 | ENr334 | 6 | 41578161 | 41595569 | 1 |
| RP11-344B5.3 | ENr232 | 9 | 129100896 | 129103415 | 1 |
| RP11-344B5.4 | ENr232 | 9 | 129100188 | 129101680 | 1 |
| RP11-398K22.14 | ENr223 | 6 | 74136149 | 74137350 | 1 |
| RP11-398K22.4 | ENr223 | 6 | 74129122 | 74130616 | 1 |
| RP1-149A16.11 | ENm004 | 22 | 31033633 | 31036187 | 1 |
| RP11-69I8.2 | ENr222 | 6 | 132264797 | 132283399 | 1 |
| RP11-88E10.3 | ENr132 | 13 | 112349360 | 112386813 | 1 |
| RP3-523C21.1 | ENr222 | 6 | 132494749 | 132532208 | 1 |
| SLC5A1 | ENm004 | 22 | 30763574 | 30833571 | 1 |
| SLC5A4 | ENm004 | 22 | 30939020 | 30975883 | -1 |
| SPP2 | ENr131 | 2 | 234741324 | 234767779 | 1 |
| SYNJ1 | ENm005 | 21 | 32922945 | 33022184 | -1 |
| TH | ENm011 | 11 | 2141736 | 2149684 | -1 |
| UBQLN3 | ENm009 | 11 | 5485107 | 5487792 | -1 |
| UGT1A1 | ENr131 | 2 | 234450895 | 234463946 | 1 |
| UGT1A11P | ENr131 | 2 | 234294200 | 234295048 | 1 |
| UGT1A12P | ENr131 | 2 | 234276086 | 234276938 | 1 |
| UGT1A13P | ENr131 | 2 | 234338573 | 234339673 | 1 |
| UGT1A3 | ENr131 | 2 | 234419755 | 234463946 | 1 |
| UGT1A4 | ENr131 | 2 | 234409425 | 234463946 | 1 |
| UGT1A5 | ENr131 | 2 | 234403639 | 234463946 | 1 |
| UGT1A6 | ENr131 | 2 | 234382254 | 234463947 | 1 |
| UGT1A7 | ENr131 | 2 | 234372585 | 234463946 | 1 |
| UGT1A8 | ENr131 | 2 | 234308292 | 234463957 | 1 |
| UGT1A9 | ENr131 | 2 | 234362500 | 234463947 | 1 |
