# Supplementary material for: Complex Exon-Intron Marking by Histone Modifications Is Not Determined Solely by Nucleosome Distribution
Source: PLoS One. 2010 Aug 23;5(8):e12339. doi: 10.1371/journal.pone.0012339 (PMC2925886; doi:10.1371/journal.pone.0012339)
Supplement: Table S5 — Expressed genes in the U937 cell line across the ENCODE regions. Expressed genes were determined as described in Materials and Methods and this list reflects the intersecting top two quartiles of expression values obtained from Affymetrix GeneChip® and Sanger Institute microarray expression studies. Gene ID/name is shown in the first column. The ENCODE region, chromosome co-ordinates [(NCBI human genome build 35 (hg17)] and direction of transcript/strand are also shown in the additional columns. (0.57 MB DOC) [file pone.0012339.s024.doc]

| **Gene ID** | **Region** | **Chr** | **Start** | **End** | **Strand** |
| --- | --- | --- | --- | --- | --- |
| AC000059.1 | ENm013 | 7 | 89905872 | 89906534 | 1 |
| AC000110.1 | ENm014 | 7 | 126529626 | 126530209 | 1 |
| AC000111.4 | ENm001 | 7 | 116721743 | 116721935 | 1 |
| AC000123.4 | ENm014 | 7 | 126584134 | 126585529 | 1 |
| AC000362.1 | ENm014 | 7 | 126214973 | 126215784 | 1 |
| AC002465.2 | ENm001 | 7 | 116534867 | 116556242 | 1 |
| AC004079.2 | ENm010 | 7 | 26861029 | 26861488 | 1 |
| AC004079.4 | ENm010 | 7 | 26836598 | 26837769 | 1 |
| AC004079.7 | ENm010 | 7 | 26908984 | 26913118 | 1 |
| AC004080.15 | ENm010 | 7 | 26981759 | 26984775 | 1 |
| AC004080.17 | ENm010 | 7 | 27014890 | 27020119 | 1 |
| AC004237.1 | ENm002 | 5 | 132052273 | 132085175 | 1 |
| AC004500.5 | ENm002 | 5 | 132238987 | 132240561 | -1 |
| AC004775.1 | ENm002 | 5 | 132111037 | 132117756 | 1 |
| AC006153.3 | ENm013 | 7 | 89614337 | 89659127 | 1 |
| AC006293.1 | ENm007 | 19 | 59912199 | 59916547 | 1 |
| AC006293.3 | ENm007 | 19 | 59913804 | 59916768 | 1 |
| AC006326.2 | ENm001 | 7 | 116497868 | 116498400 | 1 |
| AC007568.1 | ENm001 | 7 | 117116865 | 117117681 | 1 |
| AC008746.5 | ENm007 | 19 | 59649821 | 59650038 | 1 |
| AC009303.2 | ENr121 | 2 | 118487981 | 118489480 | 1 |
| AC009404.2 | ENr121 | 2 | 118307744 | 118315465 | 1 |
| AC009404.7 | ENr121 | 2 | 118304691 | 118313637 | 1 |
| AC009502.2 | ENr331 | 2 | 220388027 | 220388442 | 1 |
| AC009802.2 | ENr122 | 18 | 59767516 | 59799989 | 1 |
| AC009892.2 | ENm007 | 19 | 59755066 | 59755352 | 1 |
| AC009892.5 | ENm007 | 19 | 59811150 | 59811656 | 1 |
| AC009892.9 | ENm007 | 19 | 59855348 | 59855493 | 1 |
| AC009955.4 | ENr331 | 2 | 220233891 | 220240823 | 1 |
| AC010492.2 | ENm007 | 19 | 59424724 | 59425901 | 1 |
| AC010492.5 | ENm007 | 19 | 59463672 | 59464712 | 1 |
| AC011330.13 | ENr233 | 15 | 41721880 | 41722514 | -1 |
| AC011330.6 | ENr233 | 15 | 41728614 | 41728771 | 1 |
| AC011515.2 | ENm007 | 19 | 59874495 | 59875855 | 1 |
| AC011515.3 | ENm007 | 19 | 59900196 | 59904496 | 1 |
| AC021607.1 | ENr122 | 18 | 59890372 | 59890867 | 1 |
| AC023356.3 | ENr233 | 15 | 41955340 | 41955735 | -1 |
| AC034228.2 | ENm002 | 5 | 131375041 | 131376792 | 1 |
| AC034228.4 | ENm002 | 5 | 131308001 | 131331787 | 1 |
| AC051649.10 | ENm011 | 11 | 1868985 | 1870069 | 1 |
| AC051649.12 | ENm011 | 11 | 1841984 | 1844474 | 1 |
| AC063976.7 | ENm002 | 5 | 131563049 | 131567080 | 1 |
| AC068580.6 | ENm011 | 11 | 1738155 | 1740293 | 1 |
| AC068610.3 | ENm012 | 7 | 114312964 | 114360320 | 1 |
| AC073472.1 | ENm010 | 7 | 26734970 | 26735772 | 1 |
| AC080091.1 | ENr112 | 2 | 51648932 | 51650273 | 1 |
| AC098789.1 | ENm007 | 19 | 59463671 | 59464709 | 1 |
| AC104389.32 | ENm009 | 11 | 5340422 | 5340656 | 1 |
| AC113617.1 | ENr113 | 4 | 118639385 | 118651612 | 1 |
| AC116366.4 | ENm002 | 5 | 131774228 | 131839636 | 1 |
| AC139143.1 | ENm011 | 11 | 1770360 | 1770677 | 1 |
| ADTB1L1 | ENm004 | 22 | 30842519 | 30854011 | 1 |
| ADTB1L2 | ENm004 | 22 | 30854570 | 30854675 | 1 |
| AF064859.2 | ENr133 | 21 | 39421365 | 39421837 | 1 |
| AF129408.14 | ENr133 | 21 | 39510421 | 39511303 | 1 |
| AF277315.13 | ENm006 | X | 153446525 | 153447304 | 1 |
| AF277315.17 | ENm006 | X | 153424441 | 153425153 | -1 |
| AL162151.1 | ENr322 | 14 | 98508970 | 98509392 | -1 |
| AL162151.3 | ENr322 | 14 | 98509405 | 98509562 | 1 |
| AP000271.1 | ENm005 | 21 | 32790994 | 32791493 | 1 |
| AP000279.69 | ENm005 | 21 | 33022298 | 33037305 | 1 |
| AP000295.7 | ENm005 | 21 | 33558049 | 33560436 | -1 |
| AP000297.1 | ENm005 | 21 | 33628748 | 33629273 | 1 |
| AP000569.8 | ENm005 | 21 | 34225335 | 34271863 | 1 |
| AP000936.1 | ENm003 | 11 | 116411724 | 116411978 | -1 |
| AP001187.10 | ENr332 | 11 | 64418595 | 64438549 | -1 |
| AP001187.11 | ENr332 | 11 | 64405584 | 64405994 | 1 |
| AP001462.7 | ENr332 | 11 | 64326241 | 64326612 | 1 |
| AP003774.4 | ENr332 | 11 | 63973123 | 63975703 | 1 |
| AP006216.10 | ENm003 | 11 | 116149316 | 116150132 | 1 |
| AP006216.9 | ENm003 | 11 | 116219329 | 116332991 | -1 |
| APOA4 | ENm003 | 11 | 116196630 | 116199233 | -1 |
| ARD1 | ENm006 | X | 152715543 | 152721524 | -1 |
| ARF5 | ENm014 | 7 | 126822351 | 126825711 | 1 |
| ARHGAP26 | ENr212 | 5 | 142130134 | 142374156 | 1 |
| ASCL2 | ENm011 | 11 | 2246302 | 2248759 | -1 |
| ATF4P | ENm006 | X | 153372894 | 153373966 | 1 |
| ATP11A | ENr132 | 13 | 112392645 | 112589484 | 1 |
| ATP6AP1 | ENm006 | X | 153177832 | 153183640 | 1 |
| AVPR2 | ENm006 | X | 152688833 | 152693468 | 1 |
| AXIN1 | ENm008 | 16 | 277442 | 342675 | -1 |
| BIRC4 | ENr324 | X | 122719110 | 122773365 | 1 |
| BRCC3 | ENm006 | X | 153863400 | 153915054 | 1 |
| BUD13 | ENm003 | 11 | 116124097 | 116148915 | -1 |
| BXDC1P | ENr333 | 20 | 33804151 | 33804929 | 1 |
| C16orf33 | ENm008 | 16 | 43011 | 47670 | 1 |
| C16orf35 | ENm008 | 16 | 74274 | 128860 | -1 |
| C20orf173 | ENr333 | 20 | 33578214 | 33580892 | -1 |
| C20orf52 | ENr333 | 20 | 33750609 | 33752321 | 1 |
| C21orf119 | ENm005 | 21 | 32687311 | 32688141 | 1 |
| C21orf120 | ENm005 | 21 | 33084856 | 33085687 | -1 |
| C21orf59 | ENm005 | 21 | 32886261 | 32907048 | -1 |
| C5orf35 | ENr221 | 5 | 56240845 | 56248933 | 1 |
| C6orf148 | ENr223 | 6 | 73975315 | 74076660 | -1 |
| C6orf150 | ENr223 | 6 | 74179960 | 74218721 | -1 |
| C6orf49 | ENr334 | 6 | 41856066 | 41865858 | 1 |
| CAPZA2 | ENm001 | 7 | 116045076 | 116153267 | 1 |
| CAV2 | ENm001 | 7 | 115522079 | 115742547 | 1 |
| CCDC93 | ENr121 | 2 | 118389285 | 118487940 | -1 |
| CEP250 | ENr333 | 20 | 33506400 | 33563219 | 1 |
| CGN | ENr231 | 1 | 148296060 | 148324242 | 1 |
| CMPK | SCL | 1 | 47511502 | 47556532 | 1 |
| CNOT3 | ENm007 | 19 | 59333257 | 59351259 | 1 |
| CPNE1 | ENr333 | 20 | 33677368 | 33725881 | -1 |
| CRYZL1 | ENm005 | 21 | 33883518 | 33938103 | -1 |
| CSF2 | ENm002 | 5 | 131437383 | 131439759 | 1 |
| CTA-415G2.2 | ENm004 | 22 | 31735520 | 31736001 | 1 |
| CTSD | ENm011 | 11 | 1725474 | 1741799 | -1 |
| CXorf12 | ENm006 | X | 152758626 | 152769494 | 1 |
| CXorf52 | ENm006 | X | 153363184 | 153363896 | 1 |
| DDX18 | ENr121 | 2 | 118288457 | 118306186 | 1 |
| DECR2 | ENm008 | 16 | 391828 | 402489 | 1 |
| DEPDC5 | ENm004 | 22 | 30474499 | 30627556 | 1 |
| DKC1 | ENm006 | X | 153554740 | 153569669 | 1 |
| DOLPP1 | ENr232 | 9 | 128922934 | 128932272 | 1 |
| DONSON | ENm005 | 21 | 33869654 | 34206506 | -1 |
| DRG1 | ENm004 | 22 | 30131553 | 30154993 | 1 |
| DSCR2 | ENr133 | 21 | 39468566 | 39477648 | -1 |
| EEF1A1 | ENr223 | 6 | 74282195 | 74288345 | -1 |
| EHD1 | ENr332 | 11 | 64375691 | 64412345 | -1 |
| EIF4ENIF1 | ENm004 | 22 | 30159904 | 30216649 | -1 |
| ELL3 | ENr233 | 15 | 41852091 | 41878624 | -1 |
| EMD | ENm006 | X | 153128405 | 153130731 | 1 |
| ERGIC3 | ENr333 | 20 | 33593185 | 33608820 | 1 |
| EVX1 | ENm010 | 7 | 27055525 | 27060692 | 1 |
| F10 | ENr132 | 13 | 112825130 | 112832622 | 1 |
| F8 | ENm006 | X | 153627768 | 153818920 | -1 |
| F8A1 | ENm006 | X | 153678329 | 153680041 | 1 |
| FAM3A | ENm006 | X | 153298195 | 153308271 | -1 |
| FAM50A | ENm006 | X | 153236209 | 153242707 | 1 |
| FAM73B | ENr232 | 9 | 128878455 | 128913916 | 1 |
| FAM83C | ENr333 | 20 | 33336949 | 33343640 | -1 |
| FBXO7 | ENm004 | 22 | 31195218 | 31219371 | 1 |
| FER1L4 | ENr333 | 20 | 33609922 | 33658899 | -1 |
| FLNA | ENm006 | X | 153097742 | 153123842 | -1 |
| FOXP4 | ENr334 | 6 | 41622143 | 41678101 | 1 |
| FRS3 | ENr334 | 6 | 41845893 | 41856183 | -1 |
| FUNDC2 | ENm006 | X | 153817960 | 153852283 | 1 |
| FZD1 | ENm013 | 7 | 90538331 | 90542764 | 1 |
| GAB3 | ENm006 | X | 153467234 | 153543563 | -1 |
| GART | ENm005 | 21 | 33798109 | 33837668 | -1 |
| GDF5 | ENr333 | 20 | 33484560 | 33505983 | -1 |
| GDF9 | ENm002 | 5 | 132224773 | 132230229 | -1 |
| GMPPA | ENr331 | 2 | 220189095 | 220197216 | 1 |
| GTPBP10 | ENm013 | 7 | 89620631 | 89665421 | 1 |
| HBG2 | ENm009 | 11 | 5230997 | 5623596 | -1 |
| HCFC1 | ENm006 | X | 152733852 | 152758106 | -1 |
| HMGN1 | ENr133 | 21 | 39636112 | 39643444 | -1 |
| HOXA1 | ENm010 | 7 | 26905853 | 26908834 | -1 |
| HOXA10 | ENm010 | 7 | 26983451 | 26993121 | -1 |
| HOXA11 | ENm010 | 7 | 26994017 | 26998083 | -1 |
| HOXA11S | ENm010 | 7 | 26998268 | 27002153 | 1 |
| HOXA13 | ENm010 | 7 | 27008263 | 27012966 | -1 |
| HOXA3 | ENm010 | 7 | 26919044 | 26953068 | -1 |
| HOXA4 | ENm010 | 7 | 26941367 | 26943659 | -1 |
| HOXA5 | ENm010 | 7 | 26953912 | 26956704 | -1 |
| HOXA9 | ENm010 | 7 | 26975298 | 26988040 | -1 |
| HYPK | ENr233 | 15 | 41875633 | 41882534 | 1 |
| IER5L | ENr232 | 9 | 129017386 | 129020096 | -1 |
| IFNAR1 | ENm005 | 21 | 33618653 | 33654039 | 1 |
| IFNAR2 | ENm005 | 21 | 33524077 | 33559840 | 1 |
| IFNGR2 | ENm005 | 21 | 33679170 | 33773526 | 1 |
| IGLCOR22-1 | ENm004 | 22 | 30920461 | 30920776 | 1 |
| IL10RB | ENm005 | 21 | 33542891 | 33591410 | 1 |
| IL13 | ENm002 | 5 | 132019855 | 132024702 | 1 |
| IL3 | ENm002 | 5 | 131424122 | 131426797 | 1 |
| INHA | ENr331 | 2 | 220259390 | 220265941 | 1 |
| IRF1 | ENm002 | 5 | 131845201 | 131854390 | -1 |
| ITFG3 | ENm008 | 16 | 224547 | 258972 | 1 |
| ITGB4BP | ENr333 | 20 | 33330129 | 33336203 | -1 |
| ITSN1 | ENm005 | 21 | 33936577 | 34194036 | 1 |
| KCNQ5 | ENr223 | 6 | 73808381 | 73965296 | 1 |
| LACE1 | ENr323 | 6 | 108722792 | 108829952 | 1 |
| LAGE3 | ENm006 | X | 153269813 | 153271301 | -1 |
| LAIR1 | ENm007 | 19 | 59557075 | 59573978 | -1 |
| LAIR2 | ENm007 | 19 | 59700913 | 59713710 | 1 |
| LEAP2 | ENm002 | 5 | 132235914 | 132238638 | 1 |
| LENG1 | ENm007 | 19 | 59350712 | 59355433 | -1 |
| LENG4 | ENm007 | 19 | 59368922 | 59385479 | -1 |
| LENG8 | ENm007 | 19 | 59651878 | 59665030 | 1 |
| LENG9 | ENm007 | 19 | 59664789 | 59666707 | -1 |
| LILRA1 | ENm007 | 19 | 59796860 | 59805368 | 1 |
| LILRA2 | ENm007 | 19 | 59776200 | 59790840 | 1 |
| LILRA3 | ENm007 | 19 | 59491667 | 59501765 | -1 |
| LILRA4 | ENm007 | 19 | 59536269 | 59542234 | -1 |
| LILRA6 | ENm007 | 19 | 59432281 | 59438943 | -1 |
| LILRB1 | ENm007 | 19 | 59820425 | 59840792 | 1 |
| LILRB2 | ENm007 | 19 | 59469488 | 59476852 | -1 |
| LSP1 | ENm011 | 11 | 1830777 | 1870074 | 1 |
| LUC7L | ENm008 | 16 | 178970 | 219464 | -1 |
| MAP3K1 | ENr221 | 5 | 56147159 | 56227737 | 1 |
| MAP4K2 | ENr332 | 11 | 64313186 | 64327290 | -1 |
| MEN1 | ENr332 | 11 | 64327565 | 64335343 | -1 |
| MFAP1 | ENr233 | 15 | 41883983 | 41904293 | -1 |
| MMP24 | ENr333 | 20 | 33305670 | 33328216 | 1 |
| MPG | ENm008 | 16 | 67007 | 75853 | 1 |
| MPP1 | ENm006 | X | 153570664 | 153612987 | -1 |
| MRPL23 | ENm011 | 11 | 1925085 | 1962329 | 1 |
| MRPL28 | ENm008 | 16 | 356929 | 360570 | -1 |
| MTCP1 | ENm006 | X | 153853602 | 153939917 | -1 |
| MYADM | ENm007 | 19 | 59061290 | 59071504 | 1 |
| NDUFA3 | ENm007 | 19 | 59297849 | 59306711 | 1 |
| NFS1 | ENr333 | 20 | 33720025 | 33750696 | -1 |
| NME4 | ENm008 | 16 | 386727 | 402489 | 1 |
| NUP188 | ENr232 | 9 | 128783312 | 128848930 | 1 |
| OLIG1 | ENm005 | 21 | 33364321 | 33366597 | 1 |
| OR51I1 | ENm009 | 11 | 5418377 | 5419321 | -1 |
| OR56B1 | ENm009 | 11 | 5686015 | 5715298 | 1 |
| OSCAR | ENm007 | 19 | 59289746 | 59297813 | -1 |
| OSTM1 | ENr323 | 6 | 108469307 | 108502639 | -1 |
| PDIA2 | ENm008 | 16 | 273154 | 277217 | 1 |
| PDIA3 | ENr233 | 15 | 41825883 | 41852770 | 1 |
| PDLIM4 | ENm002 | 5 | 131621264 | 131637047 | 1 |
| PFTK1 | ENm013 | 7 | 89740390 | 90484557 | 1 |
| PIK4CB | ENr231 | 1 | 148077487 | 148113265 | -1 |
| PIP5K1A | ENr231 | 1 | 147983499 | 148035086 | 1 |
| PISD | ENm004 | 22 | 30339032 | 30382973 | -1 |
| PLXNA3 | ENm006 | X | 153250326 | 153265694 | 1 |
| POGZ | ENr231 | 1 | 148188274 | 148245015 | -1 |
| POLR3K | ENm008 | 16 | 36408 | 43629 | -1 |
| PPP2R4 | ENr232 | 9 | 128952784 | 128990780 | 1 |
| PRPF31 | ENm007 | 19 | 59310650 | 59326956 | 1 |
| PSMB4 | ENr231 | 1 | 148185084 | 148187494 | 1 |
| PSMD4 | ENr231 | 1 | 148040253 | 148053029 | 1 |
| RAB11FIP3 | ENm008 | 16 | 415621 | 495629 | 1 |
| RASGRP2 | ENr332 | 11 | 64250960 | 64269505 | -1 |
| RBM12 | ENr333 | 20 | 33700262 | 33716253 | -1 |
| RENBP | ENm006 | X | 152721564 | 152730991 | -1 |
| RFPL3S | ENm004 | 22 | 31080448 | 31091618 | -1 |
| RFX5 | ENr231 | 1 | 148126190 | 148132907 | -1 |
| RP11-115M6.4 | ENm006 | X | 153608306 | 153609501 | -1 |
| RP1-111B22.2 | ENr323 | 6 | 108432255 | 108433466 | -1 |
| RP11-126K1.8 | ENr231 | 1 | 148126198 | 148127516 | 1 |
| RP11-144L2.1 | ENr132 | 13 | 112399147 | 112419991 | 1 |
| RP11-223E19.1 | ENr111 | 13 | 29900129 | 29900593 | 1 |
| RP11-247A12.1 | ENr232 | 9 | 128939688 | 128942100 | 1 |
| RP11-247A12.6 | ENr232 | 9 | 128936628 | 128952564 | -1 |
| RP11-247I13.3 | ENm004 | 22 | 30280024 | 30280480 | 1 |
| RP1-128O3.5 | ENr323 | 6 | 108680112 | 108680298 | 1 |
| RP1-128O3.6 | ENr323 | 6 | 108745977 | 108746302 | 1 |
| RP11-298J23.7 | ENr334 | 6 | 41863379 | 41865615 | 1 |
| RP11-328M4.2 | ENr334 | 6 | 41570570 | 41624338 | -1 |
| RP11-374F3.2 | ENr111 | 13 | 29768285 | 29768873 | 1 |
| RP11-380M3.3 | ENr223 | 6 | 73821542 | 73822302 | 1 |
| RP11-398K22.10 | ENr223 | 6 | 74089282 | 74089895 | 1 |
| RP11-398K22.11 | ENr223 | 6 | 74135000 | 74161578 | -1 |
| RP11-398K22.12 | ENr223 | 6 | 74029660 | 74067846 | 1 |
| RP11-398K22.13 | ENr223 | 6 | 74056848 | 74057707 | 1 |
| RP11-398K22.6 | ENr223 | 6 | 74093969 | 74095462 | 1 |
| RP11-398K22.9 | ENr223 | 6 | 74058407 | 74059722 | 1 |
| RP1-149A16.15 | ENm004 | 22 | 31097224 | 31097677 | 1 |
| RP1-149A16.16 | ENm004 | 22 | 31107210 | 31107410 | 1 |
| RP1-149A16.17 | ENm004 | 22 | 31104328 | 31106173 | 1 |
| RP1-149M18.3 | ENr334 | 6 | 41457109 | 41457549 | -1 |
| RP11-74C1.2 | ENr231 | 1 | 148342996 | 148343490 | 1 |
| RP1-196A12.1 | ENm008 | 16 | 441406 | 443463 | 1 |
| RP11-98F14.4 | ENr132 | 13 | 112832732 | 112834370 | 1 |
| RP1-248E1.2 | ENr222 | 6 | 132634815 | 132636165 | 1 |
| RP1-90G24.10 | ENm004 | 22 | 30925657 | 30990208 | 1 |
| RP1-90G24.5 | ENm004 | 22 | 30989923 | 30993652 | 1 |
| RP1-90G24.6 | ENm004 | 22 | 30993962 | 30997728 | 1 |
| RP3-466I7.1 | ENr323 | 6 | 108815410 | 108815978 | 1 |
| RP3-477O4.15 | ENr333 | 20 | 33564811 | 33568775 | 1 |
| RP4-614O4.5 | ENr333 | 20 | 33307257 | 33331261 | -1 |
| RP4-696P19.2 | ENr334 | 6 | 41742623 | 41743366 | 1 |
| RP5-931E15.2 | ENr324 | X | 122592560 | 122593680 | 1 |
| RP5-931E15.3 | ENr324 | X | 122624687 | 122625072 | 1 |
| RP5-931E15.4 | ENr324 | X | 122644395 | 122644837 | 1 |
| RPL10 | ENm006 | X | 153147247 | 153151528 | 1 |
| RPL37P1 | ENr333 | 20 | 33639661 | 33639944 | 1 |
| RPS17P4 | ENm004 | 22 | 30760032 | 30760438 | 1 |
| SERF2 | ENr233 | 15 | 41856578 | 41882080 | 1 |
| SERPINB10 | ENr122 | 18 | 59715389 | 59754326 | 1 |
| SERPINB2 | ENr122 | 18 | 59689907 | 59722105 | 1 |
| SERPINB8 | ENr122 | 18 | 59788140 | 59823259 | 1 |
| SF1 | ENr332 | 11 | 64288655 | 64302835 | -1 |
| SFI1 | ENm004 | 22 | 30209229 | 30339092 | 1 |
| SH3GLB2 | ENr232 | 9 | 128848870 | 128870137 | -1 |
| SIL | SCL | 1 | 47427870 | 47491840 | -1 |
| SLC10A3 | ENm006 | X | 153279350 | 153282707 | -1 |
| SLC22A4 | ENm002 | 5 | 131658036 | 131707799 | 1 |
| SLC22A5 | ENm002 | 5 | 131733344 | 131759206 | 1 |
| SLC4A3 | ENr331 | 2 | 220317793 | 220332208 | 1 |
| SNX27 | ENr231 | 1 | 148397615 | 148468995 | 1 |
| SNX3 | ENr323 | 6 | 108639120 | 108689158 | -1 |
| SON | ENm005 | 21 | 33836795 | 33871658 | 1 |
| SPAG4 | ENr333 | 20 | 33667229 | 33672386 | 1 |
| ST7 | ENm001 | 7 | 116187333 | 116464109 | 1 |
| ST7OT4 | ENm001 | 7 | 116187905 | 116202580 | 1 |
| STAG2 | ENr324 | X | 122819598 | 122962042 | 1 |
| STK11IP | ENr331 | 2 | 220288088 | 220306679 | 1 |
| TAL1 | SCL | 1 | 47393984 | 47409913 | -1 |
| TAZ | ENm006 | X | 153160702 | 153170913 | 1 |
| TES | ENm001 | 7 | 115444499 | 115492789 | 1 |
| TFPT | ENm007 | 19 | 59302133 | 59310868 | -1 |
| TMC4 | ENm007 | 19 | 59355659 | 59368757 | -1 |
| TMEM15 | ENr232 | 9 | 128787364 | 128789453 | -1 |
| TMEM50B | ENm005 | 21 | 33726663 | 33775370 | -1 |
| TMEM8 | ENm008 | 16 | 360775 | 377115 | -1 |
| TNNI2 | ENm011 | 11 | 1816796 | 1819485 | 1 |
| TP53BP1 | ENr233 | 15 | 41520750 | 41590219 | -1 |
| TRIM22 | ENm009 | 11 | 5667496 | 5692850 | 1 |
| TRIM34 | ENm009 | 11 | 5597571 | 5622205 | 1 |
| TRIM6-TRIM34 | ENm009 | 11 | 5574532 | 5622205 | 1 |
| TSEN34 | ENm007 | 19 | 59385602 | 59389334 | 1 |
| TSPAN32 | ENm011 | 11 | 2279804 | 2296007 | 1 |
| TUFT1 | ENr231 | 1 | 148325855 | 148369133 | 1 |
| U52112.12 | ENm006 | X | 152666975 | 152675292 | 1 |
| UQCRQ | ENm002 | 5 | 132230152 | 132231623 | 1 |
| WDR76 | ENr233 | 15 | 41906454 | 41947910 | 1 |
| WRB | ENr133 | 21 | 39674041 | 39691686 | 1 |
| XX-FW81657B9.5 | ENm006 | X | 153269077 | 153269823 | 1 |
| XX-FW83563B9.5 | ENm006 | X | 153146067 | 153148005 | -1 |
| YWHAH | ENm004 | 22 | 30665002 | 30678145 | 1 |
| Z69890.1 | ENm008 | 16 | 221135 | 221473 | 1 |
| Z84812.1 | ENm008 | 16 | 283 | 4091 | 1 |
| Z84812.2 | ENm008 | 16 | 4044 | 9453 | -1 |
| Z84812.3 | ENm008 | 16 | 1692 | 3352 | 1 |
| Z97634.3 | ENm008 | 16 | 376765 | 377235 | 1 |
| Z97634.5 | ENm008 | 16 | 372099 | 382962 | 1 |
| ZNF259 | ENm003 | 11 | 116153647 | 116163977 | -1 |
| ZNF687 | ENr231 | 1 | 148067168 | 148077455 | 1 |
| ZNF800 | ENm014 | 7 | 126580796 | 126665930 | -1 |
