# Supplementary material for: Complex Exon-Intron Marking by Histone Modifications Is Not Determined Solely by Nucleosome Distribution
Source: PLoS One. 2010 Aug 23;5(8):e12339. doi: 10.1371/journal.pone.0012339 (PMC2925886; doi:10.1371/journal.pone.0012339)
Supplement: Table S6 — Non-expressed genes in the U937 cell line across the ENCODE regions. Non-expressed genes were determined as described in Materials and Methods and this list reflects the intersecting bottom quartile of expression values obtained from Affymetrix GeneChip® and Sanger Institute microarray expression studies. Gene ID/name is shown in the first column. The ENCODE region, chromosome co-ordinates [(NCBI human genome build 35 (hg17)] and direction of transcript/strand are also shown in the additional columns. (0.29 MB DOC) [file pone.0012339.s025.doc]

| **Gene ID** | **Region** | **Chr** | **Start** | **End** | **Strand** |
| --- | --- | --- | --- | --- | --- |
| AC000123.2 | ENm014 | 7 | 126593791 | 126598765 | -1 |
| AC000123.3 | ENm014 | 7 | 126583839 | 126584299 | -1 |
| AC000124.1 | ENm014 | 7 | 126710889 | 126719810 | 1 |
| AC000374.1 | ENm014 | 7 | 125745141 | 125746885 | 1 |
| AC004009.1 | ENm010 | 7 | 27193422 | 27194066 | 1 |
| AC004009.2 | ENm010 | 7 | 27225024 | 27225751 | 1 |
| AC004009.3 | ENm010 | 7 | 27174703 | 27222798 | 1 |
| AC004041.2 | ENm002 | 5 | 131994181 | 132027864 | -1 |
| AC004240.2 | ENm001 | 7 | 116923719 | 116949977 | 1 |
| AC004500.4 | ENm002 | 5 | 132255409 | 132267996 | -1 |
| AC005592.2 | ENr212 | 5 | 141889745 | 142031751 | 1 |
| AC008599.2 | ENm002 | 5 | 131708274 | 131712333 | -1 |
| AC008940.1 | ENr221 | 5 | 56102384 | 56103881 | 1 |
| AC009158.1 | ENr211 | 16 | 26236969 | 26253251 | 1 |
| AC009404.5 | ENr121 | 2 | 118333234 | 118377145 | 1 |
| AC009502.3 | ENr331 | 2 | 220420338 | 220427970 | 1 |
| AC009892.8 | ENm007 | 19 | 59851206 | 59852115 | 1 |
| AC009955.5 | ENr331 | 2 | 220240957 | 220261749 | -1 |
| AC011330.12 | ENr233 | 15 | 41678883 | 41684392 | 1 |
| AC011501.2 | ENm007 | 19 | 59958288 | 59970636 | 1 |
| AC011501.4 | ENm007 | 19 | 59989728 | 59993583 | 1 |
| AC015933.2 | ENr213 | 18 | 23788482 | 23797650 | 1 |
| AC016644.1 | ENr221 | 5 | 56272892 | 56278737 | 1 |
| AC023590.1 | ENr321 | 8 | 119363663 | 119377115 | 1 |
| AC051649.13 | ENm011 | 11 | 1973004 | 1973369 | 1 |
| AC074021.1 | ENm001 | 7 | 115521386 | 115565782 | 1 |
| AC092402.5 | ENm006 | X | 153006120 | 153019603 | 1 |
| AC098784.1 | ENm007 | 19 | 59907543 | 59907826 | 1 |
| AC104389.16 | ENm009 | 11 | 5182848 | 5185115 | 1 |
| AC104389.19 | ENm009 | 11 | 5219927 | 5221344 | -1 |
| AC113188.2 | ENr321 | 8 | 119270876 | 119271391 | -1 |
| AC114812.10 | ENr131 | 2 | 234441264 | 234444303 | 1 |
| AC114812.5 | ENr131 | 2 | 234444952 | 234445992 | -1 |
| AC114812.6 | ENr131 | 2 | 234433397 | 234434662 | -1 |
| AC114812.9 | ENr131 | 2 | 234429599 | 234432604 | 1 |
| AC132217.4 | ENm011 | 11 | 2106927 | 2108043 | -1 |
| AFF4 | ENm002 | 5 | 132238971 | 132267996 | -1 |
| ANKRD43 | ENm002 | 5 | 132177179 | 132180389 | 1 |
| AP000269.3 | ENm005 | 21 | 32747874 | 32752640 | 1 |
| AP000282.3 | ENm005 | 21 | 33253067 | 33254745 | 1 |
| AP000288.2 | ENm005 | 21 | 33352006 | 33359160 | 1 |
| AP002856.5 | ENr312 | 11 | 130628528 | 130675877 | 1 |
| AP002856.7 | ENr312 | 11 | 130609644 | 130626922 | 1 |
| AP003774.5 | ENr332 | 11 | 63952210 | 63956719 | 1 |
| AP005273.1 | ENr332 | 11 | 64024902 | 64029435 | 1 |
| AP006288.1 | ENr332 | 11 | 64055093 | 64057019 | 1 |
| ASZ1 | ENm001 | 7 | 116597228 | 116662129 | -1 |
| BCL11B | ENr322 | 14 | 98705378 | 98807576 | -1 |
| C21orf55 | ENm005 | 21 | 33779708 | 33785898 | -1 |
| C21orf63 | ENm005 | 21 | 32706186 | 32809571 | 1 |
| C21orf87 | ENr133 | 21 | 39607758 | 39608757 | -1 |
| C9orf106 | ENr232 | 9 | 129162850 | 129166739 | 1 |
| CACNG6 | ENm007 | 19 | 59187355 | 59207736 | 1 |
| CACNG7 | ENm007 | 19 | 59104402 | 59139008 | 1 |
| CACNG8 | ENm007 | 19 | 59158107 | 59185282 | 1 |
| CDC42EP5 | ENm007 | 19 | 59668023 | 59676224 | -1 |
| CKMT1 | ENr233 | 15 | 41774623 | 41775340 | 1 |
| CKMT1A | ENr233 | 15 | 41772377 | 41778713 | 1 |
| CKMT1B | ENr233 | 15 | 41672545 | 41678897 | 1 |
| CTAG1A | ENm006 | X | 153377112 | 153378780 | 1 |
| CTAG1B | ENm006 | X | 153409570 | 153411238 | -1 |
| CTAG2 | ENm006 | X | 153443951 | 153445558 | -1 |
| CTGF | ENr222 | 6 | 132311010 | 132314207 | -1 |
| CYP4A22 | SCL | 1 | 47315128 | 47327434 | 1 |
| CYP4Z1 | SCL | 1 | 47245181 | 47296012 | 1 |
| DDX43 | ENr223 | 6 | 74161193 | 74184014 | 1 |
| F7 | ENr132 | 13 | 112808107 | 112822997 | 1 |
| FOXP2 | ENm012 | 7 | 113600512 | 113927779 | 1 |
| FSCN3 | ENm014 | 7 | 126825415 | 126835804 | 1 |
| GRM8 | ENm014 | 7 | 125672608 | 126487300 | -1 |
| H19 | ENm011 | 11 | 1972983 | 1979277 | -1 |
| HBA2 | ENm008 | 16 | 162847 | 163710 | 1 |
| HNT | ENr312 | 11 | 130745584 | 131036336 | 1 |
| HOXA2 | ENm010 | 7 | 26913217 | 26915546 | -1 |
| HS3ST4 | ENr211 | 16 | 25805569 | 26056511 | 1 |
| IGF2AS | ENm011 | 11 | 2118308 | 2126471 | 1 |
| IL4 | ENm002 | 5 | 132037578 | 132046268 | 1 |
| IL5 | ENm002 | 5 | 131905036 | 131920430 | -1 |
| KIF3A | ENm002 | 5 | 132056268 | 132101230 | -1 |
| KIR2DL1 | ENm007 | 19 | 59973076 | 59987311 | 1 |
| KIR2DL3 | ENm007 | 19 | 59941793 | 59956317 | 1 |
| KIR2DL4 | ENm007 | 19 | 60006879 | 60017785 | 1 |
| KIR3DL1 | ENm007 | 19 | 60019736 | 60023280 | 1 |
| KIR3DL3 | ENm007 | 19 | 59927797 | 59939816 | 1 |
| LILRA5 | ENm007 | 19 | 59510166 | 59516222 | -1 |
| LILRB5 | ENm007 | 19 | 59446076 | 59452977 | -1 |
| LRRK2 | ENr123 | 12 | 38876814 | 39049355 | 1 |
| MAP1A | ENr233 | 15 | 41590449 | 41611111 | 1 |
| MDFI | ENr334 | 6 | 41712599 | 41729963 | 1 |
| MIER3 | ENr221 | 5 | 56251187 | 56303260 | -1 |
| MOXD1 | ENr222 | 6 | 132658888 | 132691427 | -1 |
| NCR2 | ENr334 | 6 | 41411372 | 41426604 | 1 |
| OPN1LW | ENm006 | X | 152930593 | 152945355 | 1 |
| OPN1MW | ENm006 | X | 152969002 | 152982481 | 1 |
| OR51A10P | ENm009 | 11 | 5446293 | 5447229 | -1 |
| OR51B4 | ENm009 | 11 | 5278821 | 5279803 | -1 |
| OR51B5 | ENm009 | 11 | 5320393 | 5321331 | -1 |
| OR51F2 | ENm009 | 11 | 4799193 | 4800221 | 1 |
| OR51H2P | ENm009 | 11 | 4854364 | 4855269 | 1 |
| OR51I2 | ENm009 | 11 | 5431296 | 5432234 | 1 |
| OR51J1 | ENm009 | 11 | 5380404 | 5381354 | 1 |
| OR51L1 | ENm009 | 11 | 4976790 | 4977737 | 1 |
| OR51N1P | ENm009 | 11 | 4764561 | 4765512 | 1 |
| OR51Q1 | ENm009 | 11 | 5400008 | 5400961 | 1 |
| OR51T1 | ENm009 | 11 | 4859707 | 4860690 | 1 |
| OR52D1 | ENm009 | 11 | 5466514 | 5467470 | 1 |
| OR52E1P | ENm009 | 11 | 5047379 | 5048304 | 1 |
| OR52J2P | ENm009 | 11 | 5014820 | 5015758 | 1 |
| OR52J3 | ENm009 | 11 | 5024333 | 5025268 | 1 |
| OR52P1P | ENm009 | 11 | 5704309 | 5705271 | 1 |
| OR52U1P | ENm009 | 11 | 5697097 | 5698019 | 1 |
| PCDH15 | ENr114 | 10 | 55232538 | 55643815 | -1 |
| PHYHD1 | ENr232 | 9 | 128768702 | 128783872 | 1 |
| RFPL3 | ENm004 | 22 | 31075427 | 31081703 | 1 |
| RGS11 | ENm008 | 16 | 258302 | 265982 | -1 |
| RP11-257K9.7 | ENr223 | 6 | 73989989 | 73992215 | -1 |
| RP1-127L4.6 | ENm004 | 22 | 30869548 | 30879864 | -1 |
| RP1-128O3.4 | ENr323 | 6 | 108703540 | 108704444 | 1 |
| RP11-328M4.3 | ENr334 | 6 | 41578161 | 41595569 | 1 |
| RP11-344B5.4 | ENr232 | 9 | 129100188 | 129101680 | 1 |
| RP11-374F3.3 | ENr111 | 13 | 29788498 | 29792041 | -1 |
| RP11-398K22.14 | ENr223 | 6 | 74136149 | 74137350 | 1 |
| RP11-398K22.4 | ENr223 | 6 | 74129122 | 74130616 | 1 |
| RP11-398K22.7 | ENr223 | 6 | 74119507 | 74120740 | -1 |
| RP11-398K22.8 | ENr223 | 6 | 74040584 | 74076810 | -1 |
| RP1-149M18.4 | ENr334 | 6 | 41481536 | 41482278 | 1 |
| RP11-69I8.2 | ENr222 | 6 | 132264797 | 132283399 | 1 |
| RP11-73M11.2 | ENr132 | 13 | 112447765 | 112457009 | -1 |
| RP1-18D14.7 | SCL | 1 | 47403490 | 47408443 | 1 |
| RP11-90M5.1 | ENr111 | 13 | 29419741 | 29422626 | 1 |
| RP3-429G5.3 | ENr323 | 6 | 108551413 | 108587290 | 1 |
| RP3-523C21.1 | ENr222 | 6 | 132494749 | 132532208 | 1 |
| SERPINB11 | ENr122 | 18 | 59465794 | 59542104 | 1 |
| SERPINB3 | ENr122 | 18 | 59473412 | 59480178 | -1 |
| SERPINB4 | ENr122 | 18 | 59455474 | 59462513 | -1 |
| SLC5A1 | ENm004 | 22 | 30763574 | 30833571 | 1 |
| SLC5A4 | ENm004 | 22 | 30939020 | 30975883 | -1 |
| SPP2 | ENr131 | 2 | 234741324 | 234767779 | 1 |
| ST7OT2 | ENm001 | 7 | 116306078 | 116380486 | -1 |
| TH | ENm011 | 11 | 2141736 | 2149684 | -1 |
| TIMP3 | ENm004 | 22 | 31522242 | 31583585 | 1 |
| TKTL1 | ENm006 | X | 153044872 | 153079548 | 1 |
| TTYH1 | ENm007 | 19 | 59618186 | 59639893 | 1 |
| UBQLN3 | ENm009 | 11 | 5485107 | 5487792 | -1 |
| UGT1A1 | ENr131 | 2 | 234450895 | 234463946 | 1 |
| UGT1A11P | ENr131 | 2 | 234294200 | 234295048 | 1 |
| UGT1A12P | ENr131 | 2 | 234276086 | 234276938 | 1 |
| UGT1A13P | ENr131 | 2 | 234338573 | 234339673 | 1 |
| UGT1A3 | ENr131 | 2 | 234419755 | 234463946 | 1 |
| UGT1A4 | ENr131 | 2 | 234409425 | 234463946 | 1 |
| UGT1A5 | ENr131 | 2 | 234403639 | 234463946 | 1 |
| UGT1A6 | ENr131 | 2 | 234382254 | 234463947 | 1 |
| UGT1A7 | ENr131 | 2 | 234372585 | 234463946 | 1 |
| UGT1A8 | ENr131 | 2 | 234308292 | 234463957 | 1 |
| UGT1A9 | ENr131 | 2 | 234362500 | 234463947 | 1 |
| WNT2 | ENm001 | 7 | 116510637 | 116557295 | -1 |
| Z84721.4 | ENm008 | 16 | 158679 | 159334 | 1 |
