# Supplementary material for: Complex Exon-Intron Marking by Histone Modifications Is Not Determined Solely by Nucleosome Distribution
Source: PLoS One. 2010 Aug 23;5(8):e12339. doi: 10.1371/journal.pone.0012339 (PMC2925886; doi:10.1371/journal.pone.0012339)
Supplement: Table S7 — Expressed genes in CD14+ monocytes across the ENCODE regions. Expressed genes were determined as described in Materials and Methods and this list reflects the top two quartiles of expression values obtained from Illumina BeadChip® expression studies. Gene ID/name is shown in the first column. The ENCODE region, chromosome co-ordinates [(NCBI human genome build 35 (hg17)] and direction of transcript/strand are also shown in the additional columns. (0.51 MB DOC) [file pone.0012339.s026.doc]

| **Gene ID** | **Region** | **Chr** | **Start** | **End** | **Strand** |
| --- | --- | --- | --- | --- | --- |
| AC000059.1 | ENm013 | 7 | 89905872 | 89906534 | 1 |
| AC000111.3 | ENm001 | 7 | 116794739 | 116798682 | -1 |
| AC000123.2 | ENm014 | 7 | 126593791 | 126598765 | -1 |
| AC000124.1 | ENm014 | 7 | 126710889 | 126719810 | 1 |
| AC004079.2 | ENm010 | 7 | 26861029 | 26861488 | 1 |
| AC004079.4 | ENm010 | 7 | 26836598 | 26837769 | 1 |
| AC004080.16 | ENm010 | 7 | 27011435 | 27015682 | 1 |
| AC004080.17 | ENm010 | 7 | 27014890 | 27020119 | 1 |
| AC004500.5 | ENm002 | 5 | 132238987 | 132240561 | -1 |
| AC004996.2 | ENm010 | 7 | 27082213 | 27082571 | -1 |
| AC005215.1 | ENr212 | 5 | 141926450 | 141926826 | 1 |
| AC005538.1 | ENr131 | 2 | 234524063 | 234545213 | -1 |
| AC005592.1 | ENr212 | 5 | 142105350 | 142120748 | 1 |
| AC008937.3 | ENr221 | 5 | 56231626 | 56242299 | -1 |
| AC008984.6 | ENm007 | 19 | 59531357 | 59534519 | -1 |
| AC009404.5 | ENr121 | 2 | 118333234 | 118377145 | 1 |
| AC009502.1 | ENr331 | 2 | 220374258 | 220375023 | -1 |
| AC009502.2 | ENr331 | 2 | 220388027 | 220388442 | 1 |
| AC011515.3 | ENm007 | 19 | 59900196 | 59904496 | 1 |
| AC012314.8 | ENm007 | 19 | 59314704 | 59320535 | -1 |
| AC015691.7 | ENm009 | 11 | 5549054 | 5549999 | -1 |
| AC018512.3 | ENr233 | 15 | 41806409 | 41826585 | -1 |
| AC018512.6 | ENr233 | 15 | 41873653 | 41879712 | -1 |
| AC023356.3 | ENr233 | 15 | 41955340 | 41955735 | -1 |
| AC034228.3 | ENm002 | 5 | 131367185 | 131370615 | 1 |
| AC051649.13 | ENm011 | 11 | 1973004 | 1973369 | 1 |
| AC051649.6 | ENm011 | 11 | 1968903 | 1971276 | 1 |
| AC063976.6 | ENm002 | 5 | 131556579 | 131557161 | -1 |
| AC073472.1 | ENm010 | 7 | 26734970 | 26735772 | 1 |
| AC073626.1 | ENm012 | 7 | 113531119 | 113531435 | -1 |
| AC092402.5 | ENm006 | X | 153006120 | 153019603 | 1 |
| AC092661.2 | ENr113 | 4 | 118853271 | 118854933 | -1 |
| AC097463.1 | ENr112 | 2 | 52065672 | 52066152 | -1 |
| AC104389.28 | ENm009 | 11 | 5282783 | 5483459 | -1 |
| AC104389.32 | ENm009 | 11 | 5340422 | 5340656 | 1 |
| AC114812.2 | ENr131 | 2 | 234412169 | 234412712 | -1 |
| AC114973.1 | ENr221 | 5 | 56307178 | 56307809 | -1 |
| AC116366.4 | ENm002 | 5 | 131774228 | 131839636 | 1 |
| AC116366.6 | ENm002 | 5 | 131832479 | 131836635 | -1 |
| AC139143.1 | ENm011 | 11 | 1770360 | 1770677 | 1 |
| AC139143.2 | ENm011 | 11 | 1780696 | 1781902 | -1 |
| ACCN4 | ENr331 | 2 | 220204398 | 220229000 | 1 |
| ADTB1L1 | ENm004 | 22 | 30842519 | 30854011 | 1 |
| ADTB1L2 | ENm004 | 22 | 30854570 | 30854675 | 1 |
| AF064859.2 | ENr133 | 21 | 39421365 | 39421837 | 1 |
| AF121781.17 | ENr133 | 21 | 39704666 | 39722325 | 1 |
| AFF4 | ENm002 | 5 | 132238971 | 132267996 | -1 |
| AL162151.1 | ENr322 | 14 | 98508970 | 98509392 | -1 |
| AL163953.1 | ENr311 | 14 | 53149193 | 53149874 | 1 |
| AP000269.3 | ENm005 | 21 | 32747874 | 32752640 | 1 |
| AP000271.1 | ENm005 | 21 | 32790994 | 32791493 | 1 |
| AP000281.1 | ENm005 | 21 | 33135677 | 33135992 | -1 |
| AP000281.2 | ENm005 | 21 | 33138548 | 33144115 | -1 |
| AP000569.8 | ENm005 | 21 | 34225335 | 34271863 | 1 |
| AP000936.1 | ENm003 | 11 | 116411724 | 116411978 | -1 |
| AP001187.10 | ENr332 | 11 | 64418595 | 64438549 | -1 |
| AP001187.9 | ENr332 | 11 | 64415475 | 64417498 | -1 |
| AP002856.7 | ENr312 | 11 | 130609644 | 130626922 | 1 |
| AP003025.2 | ENr312 | 11 | 130877864 | 130915972 | -1 |
| AP003039.2 | ENr312 | 11 | 131033418 | 131038061 | -1 |
| AP003039.4 | ENr312 | 11 | 130921767 | 130948705 | 1 |
| AP006216.9 | ENm003 | 11 | 116219329 | 116332991 | -1 |
| APOA5 | ENm003 | 11 | 116165294 | 116168338 | -1 |
| ARD1 | ENm006 | X | 152715543 | 152721524 | -1 |
| ARF5 | ENm014 | 7 | 126822351 | 126825711 | 1 |
| ASCL2 | ENm011 | 11 | 2246302 | 2248759 | -1 |
| ATP11A | ENr132 | 13 | 112392645 | 112589484 | 1 |
| ATP5O | ENm005 | 21 | 34197628 | 34210155 | -1 |
| AVPR2 | ENm006 | X | 152688833 | 152693468 | 1 |
| AXIN1 | ENm008 | 16 | 277442 | 342675 | -1 |
| BCL11B | ENr322 | 14 | 98705378 | 98807576 | -1 |
| BUD13 | ENm003 | 11 | 116124097 | 116148915 | -1 |
| BXDC1P | ENr333 | 20 | 33804151 | 33804929 | 1 |
| C11orf21 | ENm011 | 11 | 2273452 | 2280856 | -1 |
| C16orf33 | ENm008 | 16 | 43011 | 47670 | 1 |
| C20orf44 | ENr333 | 20 | 33353784 | 33463359 | -1 |
| C21orf119 | ENm005 | 21 | 32687311 | 32688141 | 1 |
| C21orf120 | ENm005 | 21 | 33084856 | 33085687 | -1 |
| C21orf13 | ENr133 | 21 | 39699641 | 39739602 | -1 |
| C21orf54 | ENm005 | 21 | 33459647 | 33464825 | -1 |
| C21orf59 | ENm005 | 21 | 32886261 | 32907048 | -1 |
| C21orf66 | ENm005 | 21 | 33028082 | 33066041 | -1 |
| C21orf87 | ENr133 | 21 | 39607758 | 39608757 | -1 |
| C22orf30 | ENm004 | 22 | 30396797 | 30470681 | -1 |
| C6orf148 | ENr223 | 6 | 73975315 | 74076660 | -1 |
| C6orf150 | ENr223 | 6 | 74179960 | 74218721 | -1 |
| CACNG6 | ENm007 | 19 | 59187355 | 59207736 | 1 |
| CACNG7 | ENm007 | 19 | 59104402 | 59139008 | 1 |
| CCDC93 | ENr121 | 2 | 118389285 | 118487940 | -1 |
| CDC42BPG | ENr332 | 11 | 64347436 | 64368618 | -1 |
| CDH2 | ENr213 | 18 | 23784929 | 24011409 | -1 |
| CLDN12 | ENm013 | 7 | 89657687 | 89787368 | 1 |
| CPNE1 | ENr333 | 20 | 33677368 | 33725881 | -1 |
| CRYZL1 | ENm005 | 21 | 33883518 | 33938103 | -1 |
| CTD-2183H9.4 | ENm006 | X | 153700865 | 153711083 | -1 |
| CTTNBP2 | ENm001 | 7 | 116944657 | 117108145 | -1 |
| CXorf12 | ENm006 | X | 152758626 | 152769494 | 1 |
| DECR2 | ENm008 | 16 | 391828 | 402489 | 1 |
| DEPDC5 | ENm004 | 22 | 30474499 | 30627556 | 1 |
| DNASE1L1 | ENm006 | X | 153150947 | 153161297 | -1 |
| DOLPP1 | ENr232 | 9 | 128922934 | 128932272 | 1 |
| DONSON | ENm005 | 21 | 33869654 | 34206506 | -1 |
| DRG1 | ENm004 | 22 | 30131553 | 30154993 | 1 |
| DSCR2 | ENr133 | 21 | 39468566 | 39477648 | -1 |
| EIF4ENIF1 | ENm004 | 22 | 30159904 | 30216649 | -1 |
| EMD | ENm006 | X | 153128405 | 153130731 | 1 |
| ENPP1 | ENr222 | 6 | 132221502 | 132257989 | 1 |
| F8A1 | ENm006 | X | 153678329 | 153680041 | 1 |
| FAM3A | ENm006 | X | 153298195 | 153308271 | -1 |
| FBXO7 | ENm004 | 22 | 31195218 | 31219371 | 1 |
| FLNA | ENm006 | X | 153097742 | 153123842 | -1 |
| FRS3 | ENr334 | 6 | 41845893 | 41856183 | -1 |
| FSCN3 | ENm014 | 7 | 126825415 | 126835804 | 1 |
| FUNDC2 | ENm006 | X | 153817960 | 153852283 | 1 |
| FZD1 | ENm013 | 7 | 90538331 | 90542764 | 1 |
| G6PD | ENm006 | X | 153323311 | 153339492 | -1 |
| GART | ENm005 | 21 | 33798109 | 33837668 | -1 |
| GCC1 | ENm014 | 7 | 126814624 | 126827617 | -1 |
| GMPPA | ENr331 | 2 | 220189095 | 220197216 | 1 |
| GTPBP10 | ENm013 | 7 | 89620631 | 89665421 | 1 |
| HBA1 | ENm008 | 16 | 166680 | 167522 | 1 |
| HBA2 | ENm008 | 16 | 162847 | 163710 | 1 |
| HBD | ENm009 | 11 | 5210485 | 5213177 | -1 |
| HBE1 | ENm009 | 11 | 5246159 | 5483424 | -1 |
| HBG1 | ENm009 | 11 | 5225890 | 5227699 | -1 |
| HBG2 | ENm009 | 11 | 5230997 | 5623596 | -1 |
| HCFC1 | ENm006 | X | 152733852 | 152758106 | -1 |
| HOXA5 | ENm010 | 7 | 26953912 | 26956704 | -1 |
| HOXA6 | ENm010 | 7 | 26958256 | 26965458 | -1 |
| HS3ST4 | ENr211 | 16 | 25805569 | 26056511 | 1 |
| HYPK | ENr233 | 15 | 41875633 | 41882534 | 1 |
| IER5L | ENr232 | 9 | 129017386 | 129020096 | -1 |
| IFNAR1 | ENm005 | 21 | 33618653 | 33654039 | 1 |
| IFNAR2 | ENm005 | 21 | 33524077 | 33559840 | 1 |
| IFNGR2 | ENm005 | 21 | 33679170 | 33773526 | 1 |
| IGF2AS | ENm011 | 11 | 2118308 | 2126471 | 1 |
| IKBKG | ENm006 | X | 153333119 | 153359509 | 1 |
| IL10RB | ENm005 | 21 | 33542891 | 33591410 | 1 |
| IL5 | ENm002 | 5 | 131905036 | 131920430 | -1 |
| INS | ENm011 | 11 | 2137585 | 2139148 | -1 |
| IRF1 | ENm002 | 5 | 131845201 | 131854390 | -1 |
| ITGB4BP | ENr333 | 20 | 33330129 | 33336203 | -1 |
| ITSN1 | ENm005 | 21 | 33936577 | 34194036 | 1 |
| KATNAL1 | ENr111 | 13 | 29674768 | 29779622 | -1 |
| KIF3A | ENm002 | 5 | 132056268 | 132101230 | -1 |
| LAGE3 | ENm006 | X | 153269813 | 153271301 | -1 |
| LAIR2 | ENm007 | 19 | 59700913 | 59713710 | 1 |
| LEAP2 | ENm002 | 5 | 132235914 | 132238638 | 1 |
| LENG8 | ENm007 | 19 | 59651878 | 59665030 | 1 |
| LENG9 | ENm007 | 19 | 59664789 | 59666707 | -1 |
| LILRA1 | ENm007 | 19 | 59796860 | 59805368 | 1 |
| LILRA2 | ENm007 | 19 | 59776200 | 59790840 | 1 |
| LILRA3 | ENm007 | 19 | 59491667 | 59501765 | -1 |
| LILRA4 | ENm007 | 19 | 59536269 | 59542234 | -1 |
| LILRA5 | ENm007 | 19 | 59510166 | 59516222 | -1 |
| LILRA6 | ENm007 | 19 | 59432281 | 59438943 | -1 |
| LILRB2 | ENm007 | 19 | 59469488 | 59476852 | -1 |
| LILRB4 | ENm007 | 19 | 59847153 | 59873623 | 1 |
| LILRB5 | ENm007 | 19 | 59446076 | 59452977 | -1 |
| LL22NC03-104C7.1 | ENm004 | 22 | 31303841 | 31304745 | 1 |
| LSP1 | ENm011 | 11 | 1830777 | 1870074 | 1 |
| LUC7L | ENm008 | 16 | 178970 | 219464 | -1 |
| MAP3K1 | ENr221 | 5 | 56147159 | 56227737 | 1 |
| MAP4K2 | ENr332 | 11 | 64313186 | 64327290 | -1 |
| MDFIC | ENm012 | 7 | 114156161 | 114253208 | 1 |
| MEN1 | ENr332 | 11 | 64327565 | 64335343 | -1 |
| MFAP1 | ENr233 | 15 | 41883983 | 41904293 | -1 |
| MMP24 | ENr333 | 20 | 33305670 | 33328216 | 1 |
| MPG | ENm008 | 16 | 67007 | 75853 | 1 |
| MPP1 | ENm006 | X | 153570664 | 153612987 | -1 |
| MRPL28 | ENm008 | 16 | 356929 | 360570 | -1 |
| MTCP1 | ENm006 | X | 153853602 | 153939917 | -1 |
| MYADM | ENm007 | 19 | 59061290 | 59071504 | 1 |
| NR2E1 | ENr323 | 6 | 108593956 | 108616707 | 1 |
| OPN1LW | ENm006 | X | 152930593 | 152945355 | 1 |
| OPN1MW | ENm006 | X | 152969002 | 152982481 | 1 |
| OR51A2 | ENm009 | 11 | 4932579 | 4933520 | -1 |
| OR51A4 | ENm009 | 11 | 4923966 | 4924907 | -1 |
| OR51B5 | ENm009 | 11 | 5320393 | 5321331 | -1 |
| OR51B6 | ENm009 | 11 | 5329315 | 5330253 | 1 |
| OR51B8P | ENm009 | 11 | 5308399 | 5309167 | -1 |
| OR51G1 | ENm009 | 11 | 4901181 | 4902146 | -1 |
| OR51G2 | ENm009 | 11 | 4892526 | 4893470 | -1 |
| OR51T1 | ENm009 | 11 | 4859707 | 4860690 | 1 |
| OR52A4 | ENm009 | 11 | 5098471 | 5102320 | -1 |
| OR52H1 | ENm009 | 11 | 5522368 | 5523330 | -1 |
| OR52Y1P | ENm009 | 11 | 4771585 | 4773091 | -1 |
| OR56B1 | ENm009 | 11 | 5686015 | 5715298 | 1 |
| OSCAR | ENm007 | 19 | 59289746 | 59297813 | -1 |
| OSTM1 | ENr323 | 6 | 108469307 | 108502639 | -1 |
| P4HA2 | ENm002 | 5 | 131555431 | 131658908 | -1 |
| PDIA3 | ENr233 | 15 | 41825883 | 41852770 | 1 |
| PFTK1 | ENm013 | 7 | 89740390 | 90484557 | 1 |
| PISD | ENm004 | 22 | 30339032 | 30382973 | -1 |
| POLR3K | ENm008 | 16 | 36408 | 43629 | -1 |
| PPP2R4 | ENr232 | 9 | 128952784 | 128990780 | 1 |
| PRKCG | ENm007 | 19 | 59074257 | 59102719 | 1 |
| PRPF31 | ENm007 | 19 | 59310650 | 59326956 | 1 |
| PSMB4 | ENr231 | 1 | 148185084 | 148187494 | 1 |
| PSMD4 | ENr231 | 1 | 148040253 | 148053029 | 1 |
| PYGM | ENr332 | 11 | 64270438 | 64284346 | -1 |
| RAB11FIP3 | ENm008 | 16 | 415621 | 495629 | 1 |
| RAD50 | ENm002 | 5 | 131919611 | 132007652 | 1 |
| RASGRP2 | ENr332 | 11 | 64250960 | 64269505 | -1 |
| RBM12 | ENr333 | 20 | 33700262 | 33716253 | -1 |
| RFX5 | ENr231 | 1 | 148126190 | 148132907 | -1 |
| RGS11 | ENm008 | 16 | 258302 | 265982 | -1 |
| RP1-111B22.2 | ENr323 | 6 | 108432255 | 108433466 | -1 |
| RP11-120K24.2 | ENr132 | 13 | 112669825 | 112671140 | -1 |
| RP11-126K1.2 | ENr231 | 1 | 148065574 | 148067479 | -1 |
| RP11-247A12.2 | ENr232 | 9 | 129018605 | 129052382 | 1 |
| RP11-247A12.6 | ENr232 | 9 | 128936628 | 128952564 | -1 |
| RP11-247I13.3 | ENm004 | 22 | 30280024 | 30280480 | 1 |
| RP11-247I13.6 | ENm004 | 22 | 30258893 | 30259130 | -1 |
| RP1-128O3.6 | ENr323 | 6 | 108745977 | 108746302 | 1 |
| RP11-344B5.2 | ENr232 | 9 | 129124292 | 129127562 | 1 |
| RP11-374F3.2 | ENr111 | 13 | 29768285 | 29768873 | 1 |
| RP11-374F3.4 | ENr111 | 13 | 29812408 | 29849283 | -1 |
| RP11-398K22.12 | ENr223 | 6 | 74029660 | 74067846 | 1 |
| RP11-398K22.13 | ENr223 | 6 | 74056848 | 74057707 | 1 |
| RP11-398K22.3 | ENr223 | 6 | 74139553 | 74139708 | -1 |
| RP11-398K22.4 | ENr223 | 6 | 74129122 | 74130616 | 1 |
| RP11-398K22.9 | ENr223 | 6 | 74058407 | 74059722 | 1 |
| RP11-490N5.1 | ENr111 | 13 | 29626204 | 29626724 | 1 |
| RP1-149A16.15 | ENm004 | 22 | 31097224 | 31097677 | 1 |
| RP11-65J3.6 | ENr232 | 9 | 129221975 | 129222304 | -1 |
| RP11-69I8.2 | ENr222 | 6 | 132264797 | 132283399 | 1 |
| RP11-74C1.2 | ENr231 | 1 | 148342996 | 148343490 | 1 |
| RP1-18D14.7 | SCL | 1 | 47403490 | 47408443 | 1 |
| RP1-191J18.65 | ENr323 | 6 | 108412987 | 108413588 | -1 |
| RP11-98F14.4 | ENr132 | 13 | 112832732 | 112834370 | 1 |
| RP1-248E1.2 | ENr222 | 6 | 132634815 | 132636165 | 1 |
| RP1-90G24.10 | ENm004 | 22 | 30925657 | 30990208 | 1 |
| RP1-90G24.5 | ENm004 | 22 | 30989923 | 30993652 | 1 |
| RP1-90G24.8 | ENm004 | 22 | 30879996 | 30880934 | -1 |
| RP3-429G5.3 | ENr323 | 6 | 108551413 | 108587290 | 1 |
| RP3-477O4.15 | ENr333 | 20 | 33564811 | 33568775 | 1 |
| RP3-477O4.5 | ENr333 | 20 | 33572095 | 33574416 | -1 |
| RP3-523C21.2 | ENr222 | 6 | 132447918 | 132462113 | 1 |
| RP5-931E15.4 | ENr324 | X | 122644395 | 122644837 | 1 |
| RPL10 | ENm006 | X | 153147247 | 153151528 | 1 |
| RPL36P4 | ENr333 | 20 | 33595590 | 33595907 | -1 |
| RPS17P4 | ENm004 | 22 | 30760032 | 30760438 | 1 |
| RPS5L | ENm005 | 21 | 33775758 | 33776373 | -1 |
| RPS9 | ENm007 | 19 | 59396423 | 59444675 | 1 |
| SELENBP1 | ENr231 | 1 | 148149852 | 148158283 | -1 |
| SERPINB10 | ENr122 | 18 | 59715389 | 59754326 | 1 |
| SERPINB2 | ENr122 | 18 | 59689907 | 59722105 | 1 |
| SERPINB8 | ENr122 | 18 | 59788140 | 59823259 | 1 |
| SETD3 | ENr322 | 14 | 98933837 | 98950025 | -1 |
| SF1 | ENr332 | 11 | 64288655 | 64302835 | -1 |
| SFI1 | ENm004 | 22 | 30209229 | 30339092 | 1 |
| SH3BGR | ENr133 | 21 | 39739652 | 39739895 | 1 |
| SH3GLB2 | ENr232 | 9 | 128848870 | 128870137 | -1 |
| SHROOM1 | ENm002 | 5 | 132185733 | 132194490 | -1 |
| SIL | SCL | 1 | 47427870 | 47491840 | -1 |
| SLC10A3 | ENm006 | X | 153279350 | 153282707 | -1 |
| SLC22A4 | ENm002 | 5 | 131658036 | 131707799 | 1 |
| SLC22A5 | ENm002 | 5 | 131733344 | 131759206 | 1 |
| SLC4A3 | ENr331 | 2 | 220317793 | 220332208 | 1 |
| SLC5A1 | ENm004 | 22 | 30763574 | 30833571 | 1 |
| SLC5A4 | ENm004 | 22 | 30939020 | 30975883 | -1 |
| SNX3 | ENr323 | 6 | 108639120 | 108689158 | -1 |
| SON | ENm005 | 21 | 33836795 | 33871658 | 1 |
| ST7 | ENm001 | 7 | 116187333 | 116464109 | 1 |
| STAG2 | ENr324 | X | 122819598 | 122962042 | 1 |
| SYN3 | ENm004 | 22 | 31233094 | 31778913 | -1 |
| SYNJ1 | ENm005 | 21 | 32922945 | 33022184 | -1 |
| SYT8 | ENm011 | 11 | 1806114 | 1815328 | 1 |
| TAZ | ENm006 | X | 153160702 | 153170913 | 1 |
| TES | ENm001 | 7 | 115444499 | 115492789 | 1 |
| TFEB | ENr334 | 6 | 41759695 | 41811976 | -1 |
| TIMP3 | ENm004 | 22 | 31522242 | 31583585 | 1 |
| TMC4 | ENm007 | 19 | 59355659 | 59368757 | -1 |
| TMEM15 | ENr232 | 9 | 128787364 | 128789453 | -1 |
| TMEM8 | ENm008 | 16 | 360775 | 377115 | -1 |
| TNNT3 | ENm011 | 11 | 1897369 | 1916513 | 1 |
| TRIM22 | ENm009 | 11 | 5667496 | 5692850 | 1 |
| TRIM6 | ENm009 | 11 | 5573916 | 5590765 | 1 |
| TSEN34 | ENm007 | 19 | 59385602 | 59389334 | 1 |
| TSPAN32 | ENm011 | 11 | 2279804 | 2296007 | 1 |
| TUFT1 | ENr231 | 1 | 148325855 | 148369133 | 1 |
| UBL4 | ENm006 | X | 153275761 | 153278705 | -1 |
| UBQLN3 | ENm009 | 11 | 5485107 | 5487792 | -1 |
| UQCRQ | ENm002 | 5 | 132230152 | 132231623 | 1 |
| WNT2 | ENm001 | 7 | 116510637 | 116557295 | -1 |
| WRB | ENr133 | 21 | 39674041 | 39691686 | 1 |
| XX-FW88778H2.2 | ENm006 | X | 153121959 | 153123854 | -1 |
| YWHAH | ENm004 | 22 | 30665002 | 30678145 | 1 |
| Z69666.2 | ENm008 | 16 | 115737 | 117219 | 1 |
| Z84812.1 | ENm008 | 16 | 283 | 4091 | 1 |
| Z84812.2 | ENm008 | 16 | 4044 | 9453 | -1 |
| ZNF259 | ENm003 | 11 | 116153647 | 116163977 | -1 |
| ZNF687 | ENr231 | 1 | 148067168 | 148077455 | 1 |
| ZNF800 | ENm014 | 7 | 126580796 | 126665930 | -1 |
