# Supplementary material for: Complex Exon-Intron Marking by Histone Modifications Is Not Determined Solely by Nucleosome Distribution
Source: PLoS One. 2010 Aug 23;5(8):e12339. doi: 10.1371/journal.pone.0012339 (PMC2925886; doi:10.1371/journal.pone.0012339)
Supplement: Table S8 — Non-expressed genes in CD14+ monocytes across the ENCODE regions. Non-expressed genes were determined as described in Materials and Methods and this list reflects the bottom quartile of expression values obtained from Illumina BeadChip® expression studies. Gene ID/name is shown in the first column. The ENCODE region, chromosome co-ordinates [(NCBI human genome build 35 (hg17)] and direction of transcript/strand are also shown in the additional columns. (0.27 MB DOC) [file pone.0012339.s027.doc]

| **Gene ID** | **Region** | **Chr** | **Start** | **End** | **Strand** |
| --- | --- | --- | --- | --- | --- |
| AC000364.1 | ENm014 | 7 | 126314699 | 126324924 | -1 |
| AC002064.2 | ENm013 | 7 | 89519151 | 89585029 | 1 |
| AC004009.2 | ENm010 | 7 | 27225024 | 27225751 | 1 |
| AC004080.13 | ENm010 | 7 | 26941829 | 26965421 | -1 |
| AC004080.18 | ENm010 | 7 | 27052103 | 27054088 | -1 |
| AC005538.2 | ENr131 | 2 | 234556084 | 234559056 | -1 |
| AC005538.3 | ENr131 | 2 | 234556143 | 234558006 | -1 |
| AC005538.5 | ENr131 | 2 | 234636791 | 234646080 | -1 |
| AC006159.3 | ENm001 | 7 | 115804966 | 115848689 | -1 |
| AC006293.1 | ENm007 | 19 | 59912199 | 59916547 | 1 |
| AC006293.3 | ENm007 | 19 | 59913804 | 59916768 | 1 |
| AC006985.5 | ENr131 | 2 | 234466371 | 234524070 | 1 |
| AC008984.2 | ENm007 | 19 | 59511995 | 59540252 | -1 |
| AC008984.4 | ENm007 | 19 | 59523289 | 59524297 | -1 |
| AC009158.1 | ENr211 | 16 | 26236969 | 26253251 | 1 |
| AC009303.1 | ENr121 | 2 | 118469417 | 118470417 | 1 |
| AC009404.2 | ENr121 | 2 | 118307744 | 118315465 | 1 |
| AC009892.8 | ENm007 | 19 | 59851206 | 59852115 | 1 |
| AC009955.5 | ENr331 | 2 | 220240957 | 220261749 | -1 |
| AC011330.5 | ENr233 | 15 | 41743145 | 41763830 | -1 |
| AC011330.8 | ENr233 | 15 | 41778979 | 41797675 | -1 |
| AC012314.6 | ENm007 | 19 | 59308899 | 59309407 | -1 |
| AC023590.1 | ENr321 | 8 | 119363663 | 119377115 | 1 |
| AC051649.15 | ENm011 | 11 | 1866952 | 1868661 | -1 |
| AC053503.11 | ENr331 | 2 | 220172301 | 220207105 | -1 |
| AC073472.2 | ENm010 | 7 | 26795628 | 26796227 | -1 |
| AC080091.1 | ENr112 | 2 | 51648932 | 51650273 | 1 |
| AC087380.14 | ENm009 | 11 | 5496248 | 5502762 | -1 |
| AC092661.1 | ENr113 | 4 | 118707156 | 118969784 | 1 |
| AC106873.2 | ENm001 | 7 | 116206171 | 116206917 | -1 |
| AC113331.9 | ENm009 | 11 | 5083869 | 5085337 | -1 |
| AC113617.1 | ENr113 | 4 | 118639385 | 118651612 | 1 |
| ACSL6 | ENm002 | 5 | 131285415 | 131375770 | -1 |
| AF064858.7 | ENr133 | 21 | 39268226 | 39271571 | -1 |
| AF277315.12 | ENm006 | X | 153450769 | 153451661 | -1 |
| AP000279.69 | ENm005 | 21 | 33022298 | 33037305 | 1 |
| AP000282.3 | ENm005 | 21 | 33253067 | 33254745 | 1 |
| AP000290.7 | ENm005 | 21 | 33450809 | 33453287 | 1 |
| AP001092.5 | ENr332 | 11 | 64175344 | 64180360 | 1 |
| AP003774.4 | ENr332 | 11 | 63973123 | 63975703 | 1 |
| AP005273.1 | ENr332 | 11 | 64024902 | 64029435 | 1 |
| AP006216.5 | ENm003 | 11 | 116189131 | 116189930 | -1 |
| ASZ1 | ENm001 | 7 | 116597228 | 116662129 | -1 |
| ATP6AP1 | ENm006 | X | 153177832 | 153183640 | 1 |
| BIRC4 | ENr324 | X | 122719110 | 122773365 | 1 |
| BRWD1 | ENr133 | 21 | 39477973 | 39615356 | -1 |
| C20orf173 | ENr333 | 20 | 33578214 | 33580892 | -1 |
| C21orf55 | ENm005 | 21 | 33779708 | 33785898 | -1 |
| C21orf77 | ENm005 | 21 | 32866420 | 32870063 | -1 |
| C22orf24 | ENm004 | 22 | 30654062 | 30666059 | -1 |
| C22orf28 | ENm004 | 22 | 31108124 | 31132797 | -1 |
| CATSPER2 | ENr233 | 15 | 41707994 | 41747609 | -1 |
| CAV2 | ENm001 | 7 | 115522079 | 115742547 | 1 |
| CGN | ENr231 | 1 | 148296060 | 148324242 | 1 |
| CKMT1A | ENr233 | 15 | 41772377 | 41778713 | 1 |
| CKMT1B | ENr233 | 15 | 41672545 | 41678897 | 1 |
| CRAT | ENr232 | 9 | 128936644 | 128953023 | -1 |
| CTA-342B11.1 | ENm004 | 22 | 30683899 | 30690942 | 1 |
| CYP4A22 | SCL | 1 | 47315128 | 47327434 | 1 |
| DDX18 | ENr121 | 2 | 118288457 | 118306186 | 1 |
| EEF1A1 | ENr223 | 6 | 74282195 | 74288345 | -1 |
| EVX1 | ENm010 | 7 | 27055525 | 27060692 | 1 |
| FAM73B | ENr232 | 9 | 128878455 | 128913916 | 1 |
| FER1L4 | ENr333 | 20 | 33609922 | 33658899 | -1 |
| FRMD5 | ENr233 | 15 | 41950255 | 42003800 | -1 |
| GAB3 | ENm006 | X | 153467234 | 153543563 | -1 |
| GRM8 | ENm014 | 7 | 125672608 | 126487300 | -1 |
| H19 | ENm011 | 11 | 1972983 | 1979277 | -1 |
| H2AFB1 | ENm006 | X | 153676952 | 153677538 | 1 |
| HBQ1 | ENm008 | 16 | 170453 | 171181 | 1 |
| HOXA1 | ENm010 | 7 | 26905853 | 26908834 | -1 |
| HOXA11 | ENm010 | 7 | 26994017 | 26998083 | -1 |
| HOXA11S | ENm010 | 7 | 26998268 | 27002153 | 1 |
| HOXA13 | ENm010 | 7 | 27008263 | 27012966 | -1 |
| HOXA2 | ENm010 | 7 | 26913217 | 26915546 | -1 |
| HOXA3 | ENm010 | 7 | 26919044 | 26953068 | -1 |
| HOXA4 | ENm010 | 7 | 26941367 | 26943659 | -1 |
| IGF2 | ENm011 | 11 | 2106919 | 2138797 | -1 |
| IL3 | ENm002 | 5 | 131424122 | 131426797 | 1 |
| IL4 | ENm002 | 5 | 132037578 | 132046268 | 1 |
| INHA | ENr331 | 2 | 220259390 | 220265941 | 1 |
| KIR3DX1 | ENm007 | 19 | 59735790 | 59748866 | 1 |
| L1CAM | ENm006 | X | 152647817 | 152695525 | -1 |
| LILRB3 | ENm007 | 19 | 59411960 | 59419191 | -1 |
| MCF2L | ENr132 | 13 | 112604511 | 112802055 | 1 |
| MDFI | ENr334 | 6 | 41712599 | 41729963 | 1 |
| NCR2 | ENr334 | 6 | 41411372 | 41426604 | 1 |
| OLIG1 | ENm005 | 21 | 33364321 | 33366597 | 1 |
| OLIG2 | ENm005 | 21 | 33320024 | 33323375 | 1 |
| OR51A10P | ENm009 | 11 | 5446293 | 5447229 | -1 |
| OR51A5P | ENm009 | 11 | 4950750 | 4951691 | -1 |
| OR51A6P | ENm009 | 11 | 4867400 | 4868403 | -1 |
| OR51B2 | ENm009 | 11 | 5301166 | 5302104 | -1 |
| OR51B4 | ENm009 | 11 | 5278821 | 5279803 | -1 |
| OR51F1 | ENm009 | 11 | 4746786 | 4747745 | -1 |
| OR51I1 | ENm009 | 11 | 5418377 | 5419321 | -1 |
| OR51I2 | ENm009 | 11 | 5431296 | 5432234 | 1 |
| OR51K1P | ENm009 | 11 | 5408460 | 5409409 | -1 |
| OR51N1P | ENm009 | 11 | 4764561 | 4765512 | 1 |
| OR51P1P | ENm009 | 11 | 4992945 | 4993886 | 1 |
| OR51Q1 | ENm009 | 11 | 5400008 | 5400961 | 1 |
| OR52A1 | ENm009 | 11 | 5128816 | 5164189 | -1 |
| OR52B5P | ENm009 | 11 | 5538774 | 5539724 | 1 |
| OR52B6 | ENm009 | 11 | 5558684 | 5559691 | 1 |
| OR52D1 | ENm009 | 11 | 5466514 | 5467470 | 1 |
| OR52E2 | ENm009 | 11 | 5036457 | 5037434 | -1 |
| OR52E3P | ENm009 | 11 | 5070483 | 5071418 | 1 |
| OR52H2P | ENm009 | 11 | 5529469 | 5530370 | -1 |
| OR52J2P | ENm009 | 11 | 5014820 | 5015758 | 1 |
| OR52J3 | ENm009 | 11 | 5024333 | 5025268 | 1 |
| OR52P1P | ENm009 | 11 | 5704309 | 5705271 | 1 |
| PCDH15 | ENr114 | 10 | 55232538 | 55643815 | -1 |
| PDZK1IP1 | SCL | 1 | 47361286 | 47368737 | -1 |
| POGZ | ENr231 | 1 | 148188274 | 148245015 | -1 |
| RP11-247I13.8 | ENm004 | 22 | 30182205 | 30184745 | 1 |
| RP11-257K9.7 | ENr223 | 6 | 73989989 | 73992215 | -1 |
| RP1-128O3.5 | ENr323 | 6 | 108680112 | 108680298 | 1 |
| RP11-328M4.3 | ENr334 | 6 | 41578161 | 41595569 | 1 |
| RP11-344B5.4 | ENr232 | 9 | 129100188 | 129101680 | 1 |
| RP11-380M3.3 | ENr223 | 6 | 73821542 | 73822302 | 1 |
| RP11-398K22.7 | ENr223 | 6 | 74119507 | 74120740 | -1 |
| RP1-149A16.11 | ENm004 | 22 | 31033633 | 31036187 | 1 |
| RP1-149A16.17 | ENm004 | 22 | 31104328 | 31106173 | 1 |
| RP1-149A16.3 | ENm004 | 22 | 31097206 | 31104885 | 1 |
| RP1-149M18.4 | ENr334 | 6 | 41481536 | 41482278 | 1 |
| RP11-629E24.1 | ENr111 | 13 | 29575316 | 29581013 | -1 |
| RP1-180M12.1 | ENm004 | 22 | 30610885 | 30611975 | -1 |
| RP1-302D9.1 | ENm004 | 22 | 31825924 | 31826600 | -1 |
| SERPINB11 | ENr122 | 18 | 59465794 | 59542104 | 1 |
| SERPINB13 | ENr122 | 18 | 59412570 | 59422854 | 1 |
| SLC22A11 | ENr332 | 11 | 64079675 | 64096924 | 1 |
| SLC22A12 | ENr332 | 11 | 64114690 | 64126397 | 1 |
| SPAG4 | ENr333 | 20 | 33667229 | 33672386 | 1 |
| SPP2 | ENr131 | 2 | 234741324 | 234767779 | 1 |
| ST7OT1 | ENm001 | 7 | 116186452 | 116188340 | -1 |
| ST7OT2 | ENm001 | 7 | 116306078 | 116380486 | -1 |
| STRC | ENr233 | 15 | 41678889 | 41698291 | -1 |
| TH | ENm011 | 11 | 2141736 | 2149684 | -1 |
| TKTL1 | ENm006 | X | 153044872 | 153079548 | 1 |
| TRIM34 | ENm009 | 11 | 5597571 | 5622205 | 1 |
| TRIM5 | ENm009 | 11 | 5641364 | 5663201 | -1 |
| TRIM6-TRIM34 | ENm009 | 11 | 5574532 | 5622205 | 1 |
| U52112.12 | ENm006 | X | 152666975 | 152675292 | 1 |
| UGT1A11P | ENr131 | 2 | 234294200 | 234295048 | 1 |
| UGT1A2P | ENr131 | 2 | 234437861 | 234438726 | 1 |
| Z84723.1 | ENm008 | 16 | 12911 | 15124 | 1 |
| Z97634.3 | ENm008 | 16 | 376765 | 377235 | 1 |
| Z97634.5 | ENm008 | 16 | 372099 | 382962 | 1 |
