# Supplementary material for: Complex Exon-Intron Marking by Histone Modifications Is Not Determined Solely by Nucleosome Distribution
Source: PLoS One. 2010 Aug 23;5(8):e12339. doi: 10.1371/journal.pone.0012339 (PMC2925886; doi:10.1371/journal.pone.0012339)
Supplement: Supplementary file 10 [file pone.0012339.s020.doc]

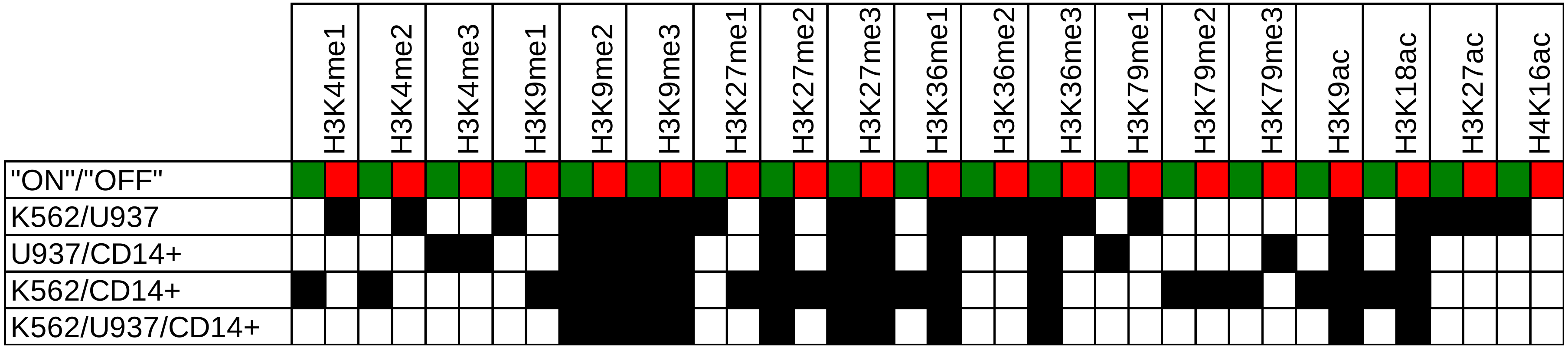
H3K4me1
H3K4me2
H3K4me3
H3K9me1
H3K9me2
H3K9me3
H3K27me1
H3K27me2
H3K27me3
H3K36me1
H3K36me2
H3K36me3
H3K79me1
H3K79me2
H3K79me3
H3K9ac
H3K18ac
H3K27ac
H4K16ac
"ON"/"OFF"
K562/U937
U937/CD14+
K562/CD14+
K562/U937/CD14+
